# Sex chromosome evolution mediated by a large inversion and a possible switch of the sex determination gene

**DOI:** 10.1186/s13059-026-04038-6

**Published:** 2026-03-19

**Authors:** Xiaomeng Mao, Nima Rafati, Christian Tellgren-Roth, Pär K. Ingvarsson, Sophie Karrenberg

**Affiliations:** 1 Department of Ecology and Genetics, Uppsala University, Norbyvägen 18 D, Uppsala , 752 36 Sweden; 2 National Bioinformatics Infrastructure Sweden, Science for Life Laboratory, Department of Medical Biochemistry and Microbiology, Uppsala University, BMC Husargatan 3, Uppsala, 752 37 Sweden; 3 National Bioinformatics Infrastructure Sweden, Science for Life Laboratory, Department of Immunology, Genetics and Pathology, Uppsala University, Rudbecklaboratoriet, 751 08 Sweden; 4 Department of Plant Biology, Swedish University of Agricultural Sciences, Box 7080, Uppsala, 750 07 Sweden

**Keywords:** Sex chromosome evolution, Inversion, Sex determination, Salicaceae

## Abstract

**Background:**

Sex chromosomes often evolve faster than autosomes and commonly degenerate after recombination arrest. However, the underlying evolutionary processes are under persistent debate. In particular, it is unclear whether or not recombination arrest generally evolves in a stepwise manner and how switches in sex determination genes contribute to sex chromosome evolution. Here, we investigate sex chromosome evolution in the dioecious plant genus *Salix*.

**Results:**

We identify Z- and W-regions (~ 8 Mb) on chromosome 15 of the dwarf willow *Salix herbacea* using a new haplotype-resolved assembly. The W-region harbours a large (5 Mb) embedded inversion. Analyses of synteny with other *Salix* species, sequence divergence between sex chromosomes and sequence degeneration suggest that this inversion recently incorporated pseudoautosomal sequence into the W-region, extending its length nearly three-fold. The W-region exclusively contains seven pairs of inverted partial repeats of the male essential floral identity gene *PISTILLATA*, suggesting a possible *PISTILLATA* suppression mechanism by interfering RNA in females*.* Such *PISTILLATA* pseudogenes are also found in other *Salix* species with ZW sex determination but not in those with XY sex determination.

**Conclusions:**

Our study provides rare and compelling support for the long-standing theory of inversions underlying stepwise recombination reduction and raises the hypothesis that the turnover of sex chromosomes in the Salicaceae family might be associated with a switch of the sex determination gene.

**Supplementary Information:**

The online version contains supplementary material available at 10.1186/s13059-026-04038-6.

## Background

Sex chromosomes have arisen from autosomes in many independent lineages but the underlying evolutionary processes are not well-understood. Sex chromosomes harbor sex determining genes that act either through male heterogamety with XX females and XY males, or through female heterogamety with ZW females and ZZ males. Chromosomes with sex-specific Y- and W-regions often experience progressive recombination arrest, followed by the accumulation of repetitive elements, sequence degeneration (i.e., Müller's ratchet), gene loss and a breakdown of synteny between sex chromosomes [1, 2]. This process may lead to microscopically visible differences between sex chromosomes (heteromorphy). Extensive recombination arrest is, however, not inevitable. On the one hand, smaller, sex-linked regions on homomorphic chromosomes can experience turnover — a change in the sex chromosome, the sex determining gene or even the sex determining system, as seen in frogs [3], fishes [4, 5] and in willows and poplars (Salicaceae) [6–8]. On the other hand, recent work shows that sex-linked regions can also remain stable over extended evolutionary time, for example, in ratite birds and sturgeons [9, 10]. These findings highlight the need to better understand the evolutionary processes underlying sex chromosome evolution.

Theoretical studies suggest at least four non-exclusive evolutionary drivers for the establishment of recombination arrest on sex chromosomes, but empirical evidence is currently scarce or controversial for all of them [11, 12]. These drivers are (1) sexually antagonistic selection, first described in the classic model by Rice [13], (2) regulatory evolution [14], (3) sheltering of deleterious mutations on the Y-or W-regions (recent update in [15]) and (4) neutral sequence divergence [16]. Theories on the first three drivers often involve (1) or require (2 and 3) recombination-suppressing inversions on the Y- or W- chromosome that capture mutations under selection and therefore predict a stepwise reduction in recombination between sex chromosomes. The fourth theory, neutral sequence divergence, in contrast, predicts a gradual decrease in recombination. Many empirical studies report discontinuous patterns of sequence divergence in X–Y or Z-W homologous genes that are assigned to evolutionary strata in accordance with theories predicting stepwise recombination reduction [12]. However, we currently lack approaches to distinguish the different drivers of recombination reduction on sex chromosomes empirically [11]. Evolutionary strata have been convincingly associated with fusions between established sex chromosomes and autosomes (neo-sex chromosomes) [17–19] but there is only very little direct evidence of inversions associated with evolutionary strata [12], for example, in humans [20] and sticklebacks [21]. Assessing the role of inversions for sex chromosome evolution remains a challenging endeavour, not only due to the need for haplotype-resolved assemblies of target and outgroup species, but also because sex chromosomes can degenerate to an extent that makes the detection of inversions impossible.

Sex determination in many animals involves an upstream sex-determining gene located on a sex chromosome that acts on downstream cascades of genes to result in either male or female development [22]. Interestingly, such upstream sex determining genes appear to change more often than the highly conserved downstream genes [22, 23]. It is unclear if a similar model applies to plants, where sex determining genes have thus far been identified in only a handful of species [24]. Two linked, sex-determining genes, a male promoter and a female suppressor, have been reported in asparagus [25] and in kiwifruit [26] in agreement with a long-standing hypothesis for the evolution of separate sexes (dioecy) from ancestors with female and male organs in each flower (hermaphroditism) [27]. Sex determination by a single gene, in contrast, has been found in persimmon [28] and in poplar [29] consistent with evolution of dioecy from species with male and female flowers on the same plant (monoecy). In both of these cases, Y-encoded small RNAs were shown to repress sex determining genes via methylation, *MeGI* and multicopy genes from the *ARABIDOPSIS RESPONSE REGULATOR 17* (*ARR17*)-like family, respectively, with downstream effects on the expression of conserved genes involved in floral identity [28–30]. Interestingly, genes related to the cytokinin signalling pathway, in particular response regulator genes, such as *RR* genes in poplar and gingko and *Shy girl* (homologous to *ARR24*) in kiwifruit, are overrepresented in candidate genes for plant sex determination [27].

The Salicaceae family contains about 450 species of shrubs and trees, almost all of which have separate sexes (dioecy) (e.g., [31]). Both ZW and XY sex determination have been reported within *Populus* (chromosome 14 or 19) and within *Salix* (chromosomes 7 or 15), suggesting numerous turnover events among dioecious species with otherwise high genome-wide synteny [7, 8]. In *Populus tremula* with XY sex determination (chromosome 19), CRISPR/Cas9-induced loss of function mutation of *ARR17* switches females to males [29]. Genetic network analysis revealed that *ARR17* is a dominant suppressor of maleness, downregulating B-class MADS-box genes involved in floral organ identity, such as *USUAL FLORAL ORGANS* (*UFO*) and *PISTILLATA* (*PI*) [30]. In *Populus* species with XY sex determination, inverted repeats of *ARR17* were detected on the Y-linked region, suggesting a possible silencing mechanism of *ARR17* by small interfering RNA in XY males [29]. In *P. alba* and *S. purpurea* with ZW sex determination systems, *ARR17* is present on the W chromosome and on autosomes but not on the Z-chromosome, but its role in ZW sex determination is currently unclear [8, 32]. Whereas sex-linked regions (SLR) described in *Populus* are small (0.2–1.7 Mb) and contain a common homologous gene, *ARR17* [8], the SLRs in *Salix* vary greatly in size, from 1.8 to 6.7 Mb, and typically contain many candidate sex determination genes [6, 7, 33, 34]. It thus remains unresolved whether there is a common sex determining mechanism in the sister genera *Populus* and *Salix*.

In this study, we analyse the evolution of sex chromosomes using a new haplotype-resolved assembly of the arctic-alpine dwarf shrub *Salix herbacea* and publicly available data from the Salicaceae family. We identified chromosome 15 as the sex chromosome with female-heterogametic (ZW) sex determination in *S. herbacea*. Our analyses suggest that a large (5 Mb) species-specific inversion on the W-chromosome recently incorporated pseudoautosomal sequence (PAR) into the sex-linked region, supporting a model of stepwise recombination suppression. We further propose a new candidate gene for sex determination in ZW *Salix* species: W-specific inverted partial repeats of the male essential gene *PISTILLATA* (*PI*) may act to suppress *PI* in females, superseding the role of the *ARR17* in sex determination.

## Results

### Genome assembly and annotation

Leveraging the long-read capabilities of PacBio HiFi sequencing and the high-resolution chromosome conformation capture provided by Hi-C, we assembled the genome of a female individual of *Salix herbacea* (Table 1, Additional file 3: Table S1). We generated the initial draft assembly using PacBio HiFi long reads. Both haplotypes were anchored by Hi-C physical mapping, which showed a well-organized pattern of contact interactions along the diagonals within pseudo-chromosomes (Additional file 1: Figs. S1-3). After manual curation, haplotype 1 and 2 comprised 328 Mb and 331 Mb, with more than 98% anchored into 19 pseudo-chromosomes (Table 1). BUSCO assessment revealed a high completeness of over 97% (Table 1, Additional file 3: Table S2). The accuracy and continuity of the genome assembly were further verified by nearly complete mapping coverage of genomic short reads (Table 1). We annotated 31,082 protein-coding genes on *S. herbacea* haplotype 1 and 30,945 on haplotype 2 (Table 1). 85% of the genes were functionally annotated in public databases [35]. Repetitive sequences comprised over 49% of both haplotype assemblies, including 21% retrotransposons (Additional file 3: Table S3).

**Table 1:** Statistics of genome assemblies of the dwarf willow *Salix herbacea*. The assemblies were generated using Pacbio HiFi sequencing and HiC reads on one female individual. The completeness was assessed using BUSCOs and the coverage of short-read sequencing of 10 individuals of each sex

	**Haplotype 1**	**Haplotype 2**
Assembly size (Mb)	327.80	330.99
Coverage of 19 pseudo-chromosomes (%)	98.85	98.24
Total scaffold number	85	63
Scaffold N_50_ (Mb)	16.66	17.16
GC content (%)	35.04	35.05
Repeat content (%)	49.01	49.33
Gene number	31,082	30,945
Complete BUSCOs (%)	97.5	98.3
Coverage of Illumina short reads (%)	99.88	99.88

### ZW sex-determination system on chromosome 15

**Fig. 1:**
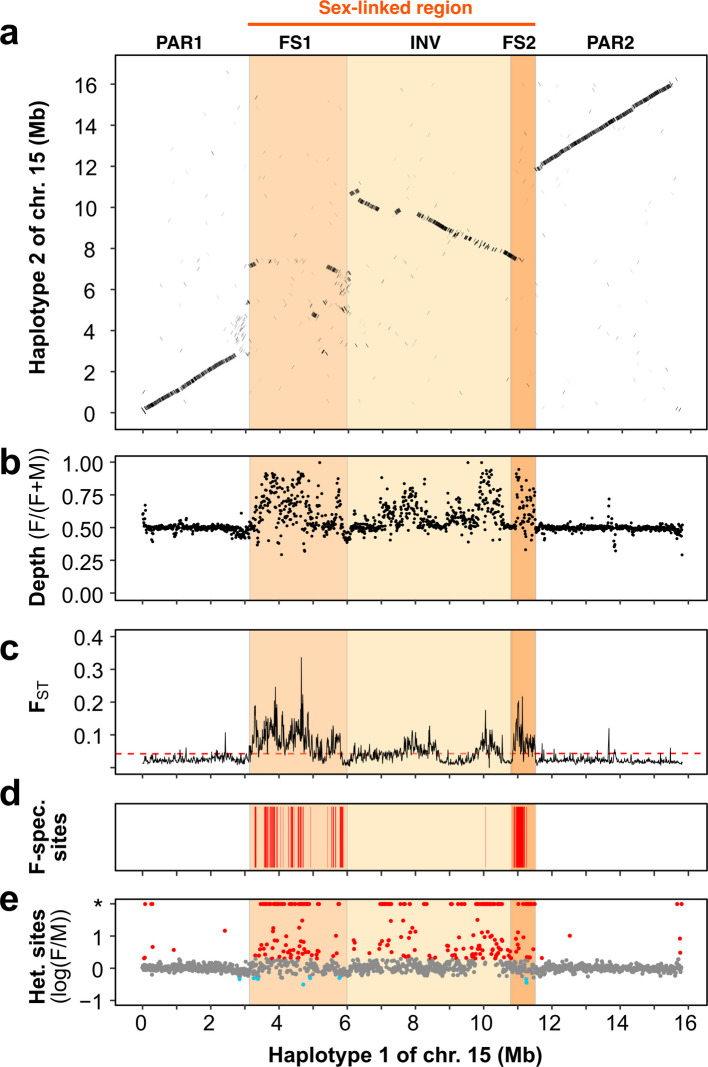
Sex-linked regions on chromosome 15 in the dwarf willow *Salix herbacea*. **a** Dot plot of an alignment of chromosome 15 between haplotype 1 and haplotype 2. **b** Sex differences in read depth in pooled sequences of 50 females (F) and 50 males (M), calculated as F/(F + M) in 10 Kb windows. **c** Genetic differentiation (F_ST_) between pools of 50 females and 50 males in 10 Kb windows with the genome-wide 99^th^ percentile indicated as a red dashed line. **d** Location of female-specific sites that were present in all 10 female individuals but absent from all 10 male individuals. **e** Ratio of the number of heterozygous sites in females (F) and in males (M) in 10 Kb windows as log (F/M); a twofold excess of female or male heterozygous sites is indicated in red and blue colour, respectively. An asterisk indicates windows showing heterozygous sites in females but not in males (0). The haplotype alignment, as well as read depth, F_ST_, and heterozygous sites for the both haplotypes and all chromosomes are shown in Additional file 1: Figs. S3-9

Analysis of read depth using short-read data of 10 females and 10 males as well as Pool-Seq data from 50 females and 50 males revealed regions with consistent and strong coverage differences between sexes on chromosome 15, encompassing more than 8 Mb (Fig. 1b, Additional file 1: Figs. S4-5). From 3.2 Mb to 11.6 Mb on haplotype 1, coverage was higher in females, whereas, in the corresponding region on haplotype 2 (from 3 to 11 Mb), coverage was higher in males, as expected for a sex-linked region in a ZW sex determination system (Additional file 1: Figs. S4-5). We also detected regions with coverage differences between sexes on chromosomes 4, 6, 7, 12, 13, 18, 19 both in pooled and individual data (Additional file 1: Figs. S4-5). However, these regions exhibited either only weak differences (chromosomes 4 and 7), mixed results on both haplotypes (chromosomes 6, 12, 18 and 19), or higher coverage in females on both haplotypes (chromosome 13).

The chromosome 15 region with coverage differences between sexes also had high between-sex genetic differentiation (F_ST_) as compared to autosomes, particularly at both ends of the region (3.2–6 Mb and 10.9–11.6 Mb on haplotype 1), where we detected exceptionally high levels of between-sex F_ST_ ​with values exceeding the genome-wide 99th percentile (Fig. 1c, Additional file 1: Figs. S6-7). We obtained similar F_ST_ results for chromosome 15 using individual re-sequencing and Pool-Seq data, and with mapping of reads to haplotype 1 and 2 (Additional file 1: Figs. S6-7). However, for the individual re-sequencing dataset, some regions with coverage difference between sexes on chromosomes 7 and 12 also exhibited weaker signals of high between-sex F_ST_. Those signals were not detected when comparing female and male pools with more individuals (Additional file 1: Figs. S6-7).

The chromosome 15 region with biased coverage and high between-sex F_ST_ was further associated with a striking excess of heterozygous sites in females compared to males (Fig. 1e, Additional file 1: Figs. S8-9). Integrating these findings, we thus find support for ZW sex-determination in *S. herbacea* on chromosome 15 with a large (8Mb) sex-linked region (SLR), where haplotype 1 corresponds to the female-specific W-haplotype and haplotype 2 to the Z-haplotype (Fig. 1).

### Species-specific structural variation between the sex chromosomes of *S. herbacea*: a large inversion within the W-SLR

**Fig. 2:**
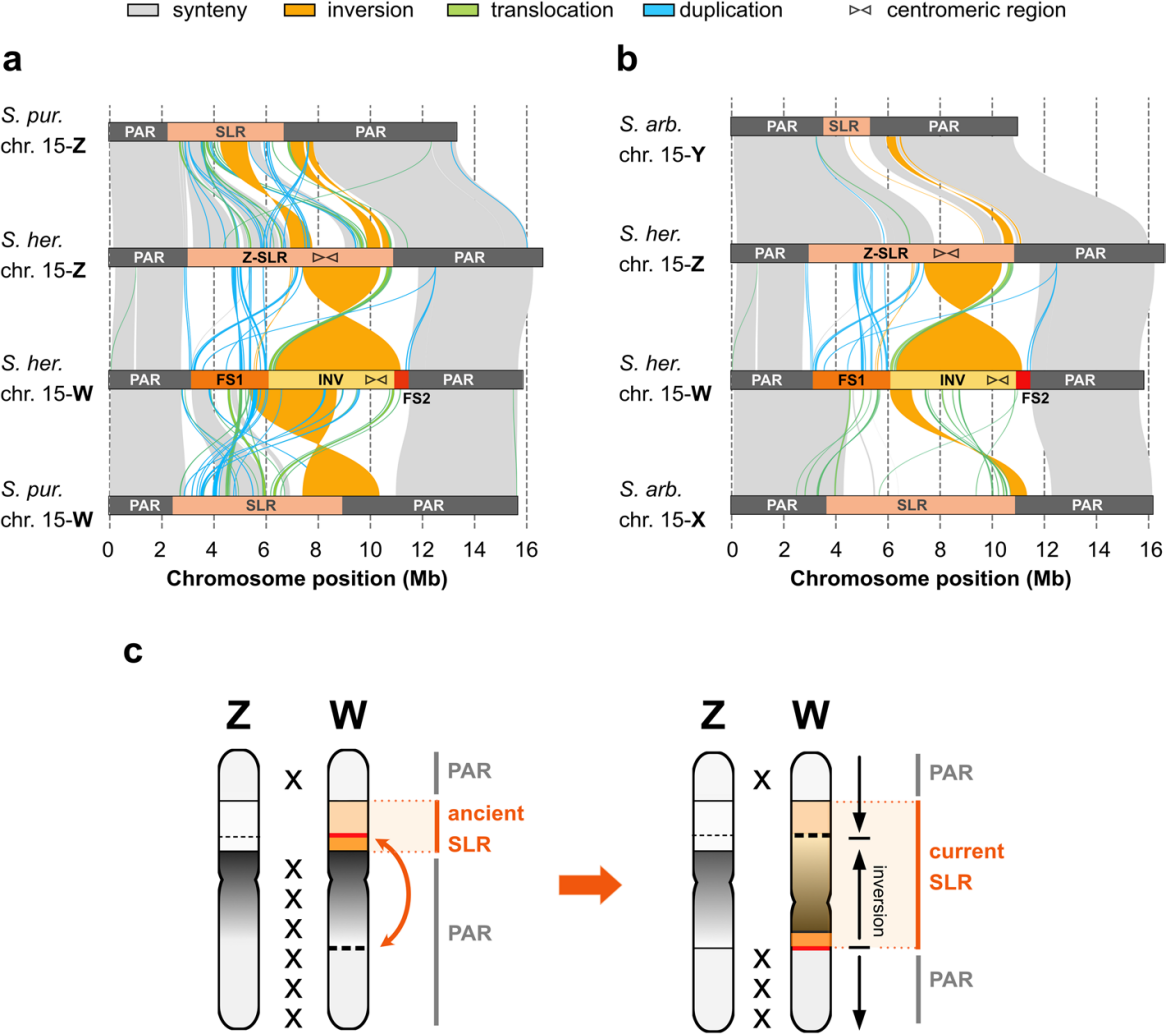
Structural variation across *Salix* species. Genomic alignment between Z and W chromosomes of *S. herbacea* (*S. her.*) with *S. purpurea* (*S. pur.,* ZW sex determination on chr. 15, (**a**) and *S. arbutifolia* (*S. arb.*, XY sex determination on chr. 15, (**b**) [6, 33]. Sex-linked regions of each haplotype are coloured in orange and PAR regions in grey or black. Centromeric regions of each haplotype are marked with inverted triangles. Inversions, translocations and duplications are shown in orange, green and blue, respectively. Grey lines show synteny alignments. **c** Illustration of inferences on the structural evolution of sex chromosomes in *S. herbacea*: step-wise recombination reduction is mediated by a large inversion that incorporates pseudoautosomal sequence into the sex-linked region

Z- and W-haplotypes had similar lengths of 16.6 and 15.8 Mb (Table 2). In an alignment of the two haplotypes, all chromosomes mapped along the diagonal, except for a fragmented gap (2.7–6.1 Mb on the W-haplotype) and an adjacent large inversion within the SLR of chromosome 15 (Fig. 1a, Additional file 1: Fig. S3). This region on the W-SLR (5.1 Mb, 6.1–11.2 Mb) is considerably larger than the corresponding region on the Z-SLR (3 Mb, 7.5–10.5 Mb). Putative centromeric regions, identified by their repeat patterns, were located within this inversion (Z-haplotype: 7.7–8.75 Mb; W-haplotype: 9.6–10.6 Mb, Fig. 2a). We can exclude that the large inversion is an artifact because the breakpoints of the inversion were covered by continuous PacBio HiFi long reads and Illumina short reads (Additional file 2: Fig. S10).

**Table 2:** Length of assembly and numbers, homology, origin and loss of genes on chromosome 15 of the dwarf willow *Salix herbacea*

Chr. 15 assembly/SLR	Length (Mb	No. genes	ZW-Homology		Origin of genes			Gene loss (prop.)	
			**ZW homologs**	**Z or W specific**	**Conserved**	**Ancestral**	*S. herbacea*** specific**	**Conserved genes**	**Ancestral genes**
H1 (W)	15.8	1288	1075	192	925	1145	143	0.15	0.18
H2 (Z)	16.6	1488	1115	263	1026	1250	238	0.06	0.11
W-SLR	8.4	426	300	109	209	350	76	0.23	0.26
Z-SLR	8	477	336	120	251	387	90	0.07	0.19

Given are length, gene numbers and gene loss, both for the entire haplotypes of chromosome 15 and for the sex-linked regions (Z-SLR, W-SLR)

Genes are divided into Z-W homologs (> 80% identity) and Z- or W- specific genes (< 60% identity), as well as according to their origin on chromosome 15 as conserved (detected on chr. 15 in three clades within the Salicaceae (clade 1: *S. purpurea* and *S. suchowensis* with ZW sex determination, clade 2: *S. arbutifolia* and *S. dunnii*, with XY sex determination, and clade 3: *Populus qiongdaoensis* and *P. trichocarpa*), ancestral (detected on chr. 15 in at least one of these six species, note that this includes conserved genes) and S*. herbacea*-specific (present on chr. 15 only in *S. herbacea*). Gene loss was estimated as the proportion of genes present divided by the joint number of conserved genes (1091 for chr. 15 and 272 for the SLR) or ancestral genes (1404 for chr. 15 and 476 for the SLR) across both haplotypes

Through a detailed whole-genome scan of coverage in 10 females and 10 males, we identified 700 female-specific regions (101,094 bp in length in total) on chromosome 15, present in all females and absent from all males. Female-specific sequences (INDEL) were detected almost exclusively in two regions at both ends of the proposed W-SLR (3.3–5.9 Mb and 10.9–11.3 Mb, Fig. 1d), corresponding to the two high-F_ST_ regions described above (Fig. 1c). We refer to these two regions with female-specific sequences as FS1 and FS2 (Fig. 1). While FS1 mapped outside of the inversion, FS2 was part of the inversion. We thus describe five regions of the W-chromosome: a first pseudoautosomal region (PAR1), FS1, the inversion (INV), FS2 and a second pseudoautosomal region (PAR2, Fig. 1). The W sex-linked region includes the regions FS1, INV and FS2 (Fig. 1).

A synteny analysis of the two chromosome 15 haplotypes of *S. herbacea* supports the inversion within the W-SLR as described above, as well as the overlap of FS2 with the inversion (Fig. 2a, b). The two other haplotype-resolved *Salix* genomes, *S. purpurea* from the same clade as *S. herbacea* (*Vetrix*), also with ZW sex determination, and *S. arbutifolia* from the *Salix* clade with XY sex determination, generally show very high synteny with *S. herbacea* across the genome (Additional file 2: Fig. S11) [6, 33]. On chromosome 15, synteny between the three species is high in the PAR regions of chromosome 15, but much reduced in the SLRs, indicating massive structural divergence between species in the SLRs (Fig. 2a, b). The Z-haplotypes of *S. herbacea*, *S. purpurea* and *S. arbutifolia* aligned well, while an alignment of W-haplotypes indicated that the large inversion was specific to the W-SLR of *S. herbacea* (Fig. 2a, b). About half of the large *S. herbacea* inversion on the W-SLR mapped to the PAR of *S. purpurea* and to a small region at the upstream (5’) end of the *S. purpurea* W-SLR, as well as to a small region within the PAR of *S. arbutifolia* (Fig. 2a, b). These findings suggest that the large W-specific inversion in *S. herbacea* incorporated part of the PAR region into the middle of the recombination-reduced W-SLR, splitting the ancestral W-SLR into two parts (FS1 and FS2, Fig. 2c).

Intriguingly, the FS1 of the W-SLR of *S. herbacea* exhibited crossed lines of synteny with the corresponding region of the *S. herbacea* Z-SLR, the W-haplotype of *S. purpurea*, and the X-haplotype of *S. arbutifolia* (Fig. 2a, b) suggesting an additional ancient, species-specific inversion event involving the FS1 of *S. herbacea*. The FS2 region of the *S. herbacea* W-SLR exhibited several translocated alignments (10.7–11.2 Mb) with the middle of the *S. purpurea* W-SLR (6.2–6.6 Mb) and the *S. arbutifolia* X-SLR (9.4–9.7 Mb) indicating that further species-specific re-arrangements formed this region.

### Repeat accumulation, gene loss and sequence degeneration on the sex chromosomes

**Fig. 3:**
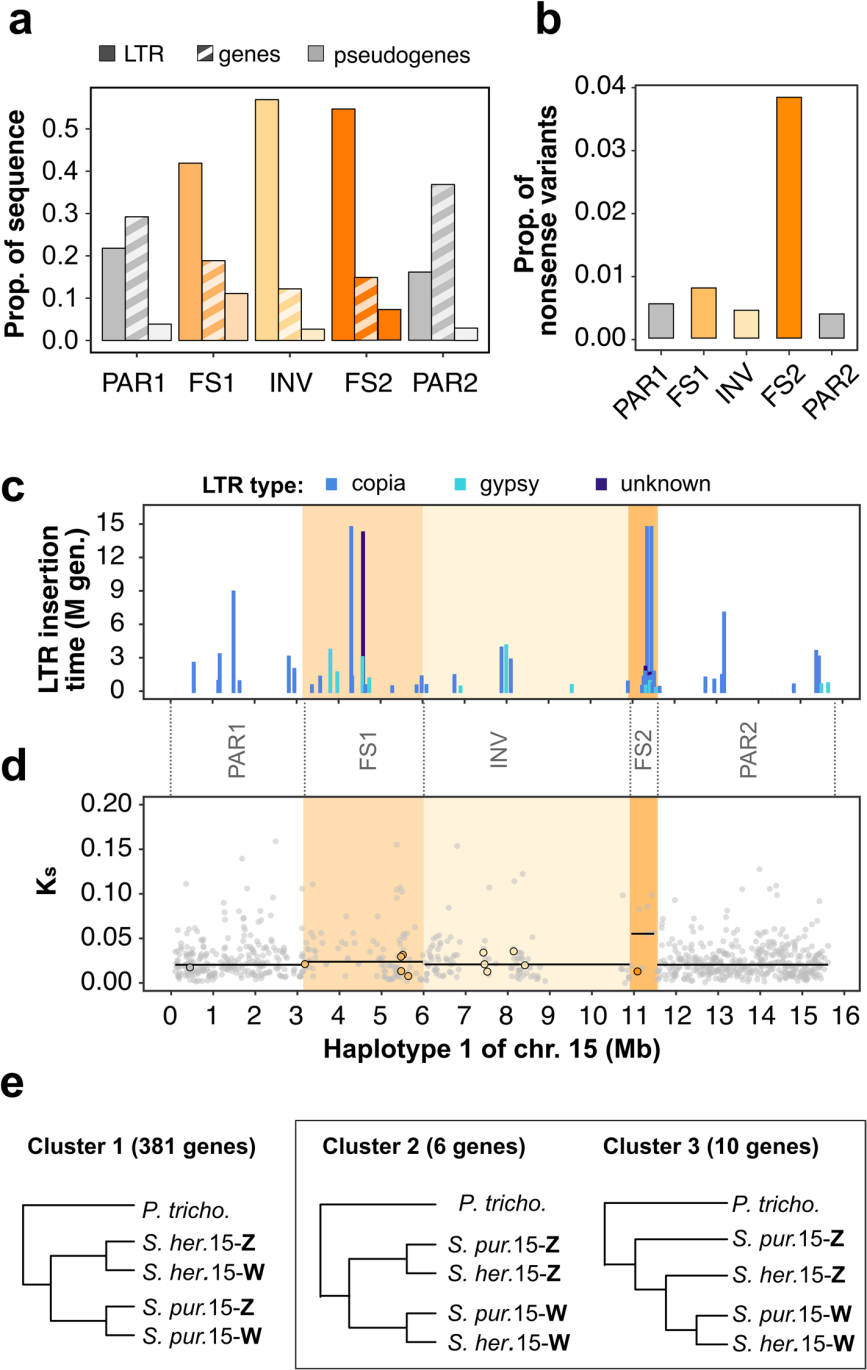
Repeat accumulation, sequence degeneration and divergence along the W-chromosome of the dwarf willow *Salix herbacea*. **a** Sequence proportions of long terminal repeats (LTRs), genes and pseudogenes (× 10) in five regions (two distal pseudoautosomal regions: PAR1, PAR2; two female-specific regions: FS1, FS2; and a central inversion region: INV). **b** Proportion of variants across the study population with predicted nonsense effect on gene function. **c** Insertion time estimates for LTRs in millions of generations (M gen.), assuming a mutation rate of 2.5 × 10^–9^ per generation, with colours distinguishing between Copia (green), Gypsy (red), and unknown LTR (purple) types. **d** Synonymous site divergence rates (K_s_) of Z-W homologous genes with median K_s_ indicated in each region. K_s_ values are shown within the range of 0–0.2 for clarity. Additional 5 genes with K_s_ > 0.2 are not shown. **e** Three primary consensus trees of single-copy orthologs (SCOs) for *S. herbacea* (Saher) and *S. purpurea* (Sapur) with *P. trichocarpa* as an outgroup. Genes supporting sex chromosome divergence before species divergence (clusters 2 and 3) are marked as black circles in d

Recombination arrest on sex chromosomes allows for the accumulation of repetitive sequences and for sequence degeneration, such as gene loss, pseudogenization and retention of mutations, over time [1]. We estimated the repeat content of the *S. herbacea* W-SLR to 80%, as compared to 40–50% in the PARs (Additional file 3: Table S5). LTR sequences, the most common repeat class, comprised up to 50% of the sequence in the W-SLR with an associated decrease in gene content, particularly in the INV region (Fig. 3a, Table 2, Additional file 3: Table S5, Additional file 2: Fig. S12 for other repeat classes). The proportion of the sequence constituting highly degenerated genes (pseudogenes) was much higher in FS1 and FS2 (0.011, 0.007) than in the inversion (INV, 0.003) and the two PAR regions (0.004 and 0.003) (Fig. 3a, Additional file 3: Table S5).

LTR insertion times along the W-chromosome ranged from over 14.6 million generations to less than 0.5 million generations ago (Fig. 3c). A clear clustering of LTR insertions, 1–2 million generations ago, occurred at the 5’ end of the inversion (~ 11.4 Mb) (Fig. 3c). This finding opens the possibility of an LTR driven inversion event; however, we cannot exclude that the inversion happened more recently.

Z- and W-haplotypes of chromosome 15 contained a total of 1488 and 1288 genes, more than 70% of which were homologous between haplotypes (Table 2). More than two thirds of the genes (69% and 72% for Z- and W-haplotypes) were also detected on chromosomes 15 in all three clades within the Salicaceae (clade 1: *S. purpurea* and *S. suchowensis* with ZW sex determination, clade 2: *S. arbutifolia* and *S. dunnii*, with XY sex determination, and clade 3: *Populus qiongdaoensis* and *P. trichocarpa*) and thus have a conserved location on this chromosome, whereas 84% (Z-haplotype) and 89% (W-haplotype) of the genes were annotated on the same chromosome in at least one of the six species and are considered as ancestral (Table 2). The remaining genes (16% and 11% on Z- and W-haplotypes) were present on chromosome 15 only in *S. herbacea*. Assuming that conserved and ancestral genes were initially present on both haplotypes, we detected considerable gene loss, particularly on the W-SLR. 26% of the ancestral and conserved genes were missing on the W-SLR, while the Z-SLR lost only 19% of ancestral and conserved genes; this pattern is even stronger when considering conserved genes only (Table 2).

We investigated degeneration in the coding sequences of genes on chromosome 15 by classifying polymorphisms in our study population (20 individuals) according to their potential impact on gene function (Fig. 3b). We detected 23,468 polymorphic loci within the coding regions of 1,030 genes. The SLR, as compared to the PARs, had slightly more missense polymorphisms that result in the transcription of different amino acids and may affect protein function (Additional file 2: Fig. S13). The proportion of polymorphisms with nonsense variants, however, was markedly raised in the SLR, including a strong increase to over 0.037 on the FS2 region on the W-SLR (Fig. 3b). Together, these results suggest a pronounced gene loss in the W-SLR of *S. herbacea* and stronger sequence degeneration in the female-specific regions of the W-SLR, particularly in FS2, as compared to the INV and PAR regions. The INV region differed from the PARs mainly by higher LTR content and reduced gene content (Table 2).

### Divergence of ZW-homologous genes on chromosome 15

We estimated sequence divergence between Z- and W- homologs in our assembly for 883 genes on chromosome 15, based on synonymous site divergence rates (K_s_) and the ratio between non-synonymous and synonymous divergence (K_a_/K_s_). Divergence was slightly higher in genes in the FS1 region and considerably elevated in genes on FS2, as compared to the INV region within the W-SLR and to the PARs at both ends of the sex chromosomes (Fig. 3d). K_a_/K_s_ exhibited a similar pattern along chromosome 15 (Additional file 2: Fig. S14) indicating that non-synonymous variants were retained more frequently in the sex-linked region, contributing to sequence degeneration. Both divergence and K_a_/K_s_ were clearly highest in the FS2 region, suggesting that FS2 might be the oldest region of the W-SLR.

**Fig. 4:**
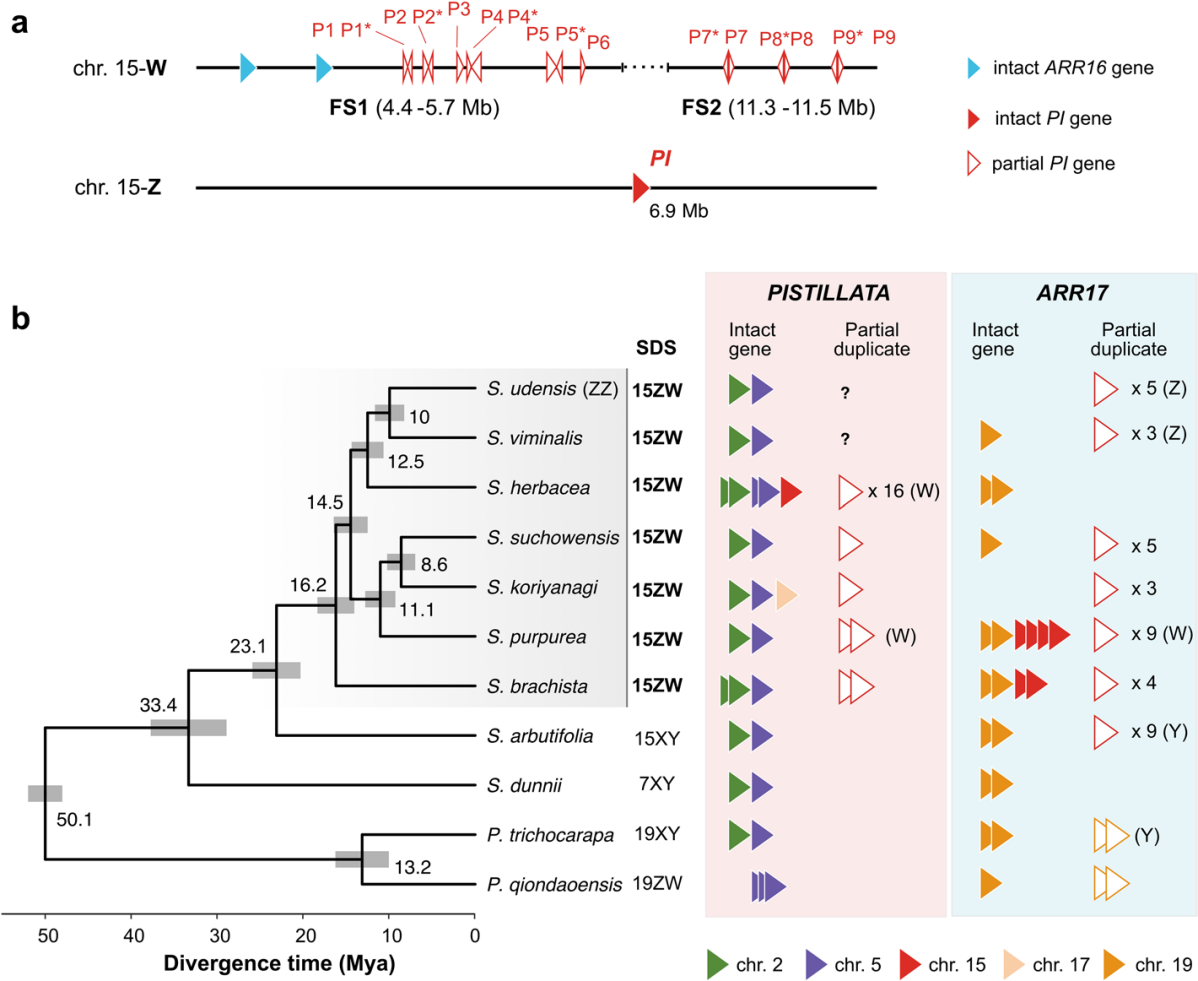
Candidate sex determination genes in *Salix* and *Populus* species*.* **a** Distribution of *PISTILLATA* (*PI*) and *ARR16* on both haplotypes of chromosome 15 in *Salix herbacea*. **b** Species phylogenetic tree and copy numbers of candidate sex-determination genes (*PI* and *ARR17*) in the genomes of nine *Salix* species and two *Populus* species. The chromosomal locations are depicted by distinct colours and complete and partial genes by full and empty triangles, respectively. References to the published assemblies used are listed in Additional file 3: Table S4

We examined phylogenetic trees of 409 ZW-homologous genes on chromosome 15 with single-copy orthologs in *S. purpurea* and *P. trichocarpa* (outgroup), and found that the great majority of the gene phylogenies followed the species phylogeny (Fig. 3e, for genomic resources used see Additional file 3: Table S4). In the phylogenies of 16 genes, Z- and W-haplotypes clustered across the two *Salix* species; 15 of these genes were in the W-SLR region (Fig. 3d, e). This indicates that the divergence of the Z- and W-SLR of *S. herbacea* occurred mainly before the lineage split between clades containing*S. herbacea* and *S. purpurea,* respectively, dated to 14.5 million years before present in our analysis (Fig. 4b).

### Putative functions of genes with sex-specific variation

We identified genes that are potentially associated with sexual function in two ways: first, we searched for genes annotated only on the W-SLR or only on the Z-SLR, and secondly, we assessed female-specific polymorphisms (INDEL or SNP) in Z-W homologous genes in our study population. In total, we detected 138 genes with sex-specific variants including 109 genes that were W-SLR specific and 29 additional genes with female-specific polymorphisms (Additional file 2: Fig. S15, Table 3, Additional file 3: Tables S6, S7). While W-SLR specific genes were detected along FS1, INV and FS2 regions, genes with female-specific polymorphisms were present only in the female-specific regions, including a cluster of 14 consecutive genes in FS2 (10.85–11.2 Mb, Additional file 2: Fig. S15, Additional file 3: Table S7). We further divided genes with sex-specific variants into conserved (33 genes, 24%), ancestral (63 genes, 46%) and *S. herbacea*-specific (42 genes, 30%), as described above (Additional file 3: Table S7). The ancestral and conserved genes contained homologs of *A. thaliana* genes related not only to floral development, such as a B-box type zinc finger protein (*BBX15*) [36] and *CRF9* [37], but also to male-function, such as the cytokinin response regulator *ARR24* [26] and *HIK*, *PAPS1*, *ENOLDL6*, *ARID*, *ARP4* that are involved in pollen and anther development [38–42] (Additional file 2: Fig. S15, Additional file 3: Table S7). *S. herbacea*-specific genes included genes homologous to two DEAD/H box RNA helicase-like genes in *A. thaliana* that are involved in female gametogenesis, *MEE29* and *NOF1* [43, 44], to *SCD1* and *SWEETIE* that are associated with inflorescence formation [45, 46]*,* and to *MiP1* that functions as a flowering activity switch [47, 48] (Additional file 2: Fig. S15, Table 3, Additional file 3: Table S7). We further examined whether female-specific polymorphisms were shared with *S. viminalis*, *S. purpurea*, or *S. suchowensis*. 13% of the sites with female-specific polymorphisms were shared with *S. viminalis* (which is the species most closely related to *S. herbacea* [31]) whereas only 0.2% of the polymorphic sites occurred in multiple species (Additional file 2: Fig. S15, Additional file 3: Table S7, Additional file 4: Dataset S1, Additional file 5: Dataset S2).

**Table 3:** Annotation of genes with sex-specific variation related to reproductive functions in the dwarf willow *Salix herbacea*

**Name**	**General description**	**Putative functions in reproductive processes in** *Arabidopsis thaliana*	**Gene ID**	**Location**	**Gene model**
*ARID*^p^	AT-rich interactive domain protein	**pollen tube growth**	g23838	W-INV	AT1G73885.1
*ARP4*^p^	actin-related protein 4	**male sterility due to defects in pollen and anther**	g23842, g23854	W-FS2	AT1G18450.1
*BBX15*^p^	B-box type zinc finger protein with CCT domain	regulates flowering time and abiotic stress	g23853	W-FS2	AT1G25440.1
*CP2*^p^	2OG and Fe(II)-dependent oxygenase superfamily protein	epigenetic repression of flowering genes	g23584	W-FS1	AT3G18210.1
*ENODL6*^p^	early nodulin-like protein 6	**seedling growth, pollen tube elongation, embryogenesis and cell proliferation**	g23836	W-INV	AT1G48940.1
GSTT3*^p^	encodes glutathione transferase	control of flowering time and cold response	g23844	W-FS2	AT5G41220.1
*HIK*^p^	encodes a kinesin HINKEL	**required for cytokinesis in pollen**	g23571	W-FS1	AT1G18370.1
MEE29*	maternal effect embryo arrest 29	female gametophyte development and function	g23522, g23843	W-FS2	AT2G35340.1
*NAC058*^p^	NAC domain containing protein 58	control of flowering time and cold response	g23453, g23454	W-FS1	AT3G18400.1
NOF1*^p^	U3 small nucleolar RNA-associated protein	female gametogenesis and embryo development	g23487, g23519, g23849	W-FS1, W-FS2	AT1G17690.1
*PAPS1*^p^	poly(A) polymerase 1	**mediates pollen maturation by regulating sperm cell differentiation**	g23583	W-FS1	AT1G17980.1
PI*	MADS domain transcription factor	**required for the specification of petal and stamen identities**	g23664	Z-SLR	AT5G20240.1
RR24*	response regulator 24	**expressed in floral buds, mature flowers, and pollen**	g23483, g23514	W-FS1	AT5G26594.1
SCD1*^p^	stomatal cytokinesis defective	seedling growth, root elongation and flower morphogenesis	g23476, g23478, g23484, g23510, g23515, g23477, g23479, g23485, g23516	W-FS1	AT1G49040.1

Annotated gene name, general description, putative function in reproductive processes are given together with Gene ID and location on the W-SLR (FS1, INV, FS2) or Z-SLR of *S. herbacea,* and gene model according to the TAIR database

Genes are listed in alphabetical order. ^p^, genes with female-specific polymorphisms in the study population; * genes annotated only on the W-SLR or Z-SLR assembly. Male-specific functions are highlighted in bold, whereas female-specific functions are underlined. For references, see methods; further genes and references are listed in Additional file 3: Tables S6, S7 and S8

The Z-SLR specific genes, that are expected in both ZZ males and ZW females, included 45 (37%) conserved, 34 (28%) ancestral and 42 (35%) *S. herbacea*-specific genes (Additional file 3: Table S8). Among the ancestral genes, we detected the well-known gene *PISTILLATA* (*PI*) which is involved in male organ identity in many species, including *Populus tremula* [27–30] and analysed in more detail below. For most of the remaining genes specific for the Z-SLR or W-SLR or with female-specific polymorphisms, the connection to floral development is unclear (Additional file 3: Tables S6, S8).

### Previously proposed sex determination gene *ARR17* not present on SLR

In *Populus* and some *Salix* species, the *ARABIDOPSIS RESPONSE REGULATOR 17* (*ARR17*) has been proposed as the single sex-determining gene [7, 29]. We did not detect any *ARR17* genes or partial duplicates on chromosome 15 in *S. herbacea*, but two *ARR16*-like genes were found in FS1 of the W-SLR (Fig. 4a) together with a homolog to *ARR5* on the Z-SLR with 65% sequence identity, which we thus classified as ZW-homologous genes. *ARR16* and *ARR17* are type-A cytokinin genes in the *RR* gene family [49]. Protein sequence identity between *ARR17* and *ARR16* and other *RR* genes was low, 68–71% between *ARR17* and *ARR16;* however, the functional consequences remain unknown (Additional file 6: Dataset S3). We searched for complete or partial homologs of *ARR16/17* in 12 other *Salix* and *Populus* species, using all exons or only the first exon. Two *ARR17*-like genes and one *ARR16*-like gene on chromosome 19 were ancestrally shared among *Salix* and *Populus*. Of the *Salix* species, only *S. purpurea* and *S. brachista*, both with female heterogamety (chr. 15 ZW), had *ARR17* genes on chromosome 15, while the other six *Salix* species with ZW sex determination lacked *ARR17* homologs on this chromosome (Fig. 4b). Partial duplicates of *ARR17* were found on chromosome 15 in 8 *Salix* species with various sex determination systems (on W, Z or Y haplotypes) but were completely missing from *S. herbacea* (chr. 15 ZW) and *S. dunnii* (chr. 7 XY, Fig. 4b). An additional *ARR16* gene on chromosome 15 W was only detected in *S. herbacea* and in the female genome assembly of *S. viminalis* (Additional file 3: Table S9).

### *PISTILLATA *(*PI*) is a new candidate sex determination gene

We found one homolog of an intact *PISTILLATA* (*PI*) gene on the Z-SLR of chromosome 15, and 16 partial duplicates of the first exon on the W-SLR, including 7 pairs of inverted repeats in the FS1 and FS2 regions and two unpaired partial duplicates (Fig. 4a). This suggests a possible mechanism of suppressing the male-essential *PI* by small interfering RNA mediated gene silencing [50]. We searched for complete or partial homologs of *PI* genes in 9 *Salix* and 2 *Populus* species by processing all exons or only the first exon. All species, including *S. herbacea,* shared conserved copies of *PI* on chromosomes 2 and 5 (Additional file 1: Fig. S16). In a phylogeny of *PI* genes, the full-length *PI* on the Z-SLR of *S. herbacea*, 6 pairs of inverted repeats and the unpaired partial duplicates clustered with the *PI* homologs on chromosome 5, while one pair of inverted repeats clustered with the *PI* homologs on chromosome 2 (Additional file 2: Fig. S16), indicating that inverted repeats originated from both chromosomes.

We detected different numbers of partial duplicates of *PI* on chromosome 15 in female assemblies of five other *Salix* species with female heterogamety on chromosome 15, but not in *S. viminalis*, possibly due to a poor assembly from short reads in that species (Fig. 4b). Inverted partial repeats of *PI* were also detected on chromosome 15 W of *S. purpurea* and in *S. brachista* (Fig. 4b, Additional file 3: Table S9). By contrast, no partial duplicates of *PI* were detected in species with XY sex determination and in the male (ZZ) assembly of *S. udensis* (Fig. 4b).

## Discussion

### A large inversion causes a three-fold extension of the sex-linked region

Using a haplotype-resolved genome assembly and population whole-genome re-sequencing data, we identified female heterogamety (ZW) on chromosome 15 as the sex determination mechanism of dwarf willow, *Salix herbacea*. The sex-linked region (SLR) comprised 8.4 Mb, 53% of chromosome 15, making it the largest reported SLR in the genus *Salix*. Y- or W-linked regions in other *Salix* species cover between 17 and 43% of the sex chromosome [6, 33], but only 2–10% in the closely related genus *Populus* [51–54]. The *S. herbacea* W-SLR contained a large (5 Mb) inversion not found in other *Salix* species. Two regions with a high incidence of female-specific sequences were detected, one at the 3’ end of the W-SLR (FS1, 2.6 Mb), adjacent to the inversion, and the other at the 5’ end of the W-SLR, as part of the inversion (FS2, 0.4 Mb). A synteny analysis with two other *Salix* species suggests that the inversion incorporated a pseudoautosomal region (PAR) into the SLR, extending its length nearly threefold. Two lines of evidence indicate that the inversion on the W-SLR likely is recent: First, synonymous sequence divergence (K_s_) between homologous genes on the two haplotypes of chromosome 15 was low in the INV region and the pseudoautosomal regions, and higher in the female-specific regions FS1 and FS2. Second, we found support for a recent burst of TE insertions close to the breakpoint of the inversion. Moreover, the inversion contained the centromere which may lead to accelerated recombination arrest in the SLR [55, 56]. Our data thus support the model of stepwise evolution of recombination suppression via inversions as a proximate mechanism [1, 11, 12], in contrast to studies in other *Salix* species [7, 33, 34]. While inversions are commonly found on sex chromosomes [18, 19, 55, 57], we report one of the few cases showing a clear link between a recent inversion and the extension of the recombination-suppressed region on sex chromosomes [12].

### Evolution of the sex-linked region

Plant sex chromosomes are often, but not always, described as young, with limited degeneration, small SLRs and frequent turnover [58]. This has also been suggested for several *Salix* and *Populus* species [7, 29]. The dwarf willow, *S. herbacea,* does not fit into this pattern. The INV region on the W-SLR is highly collinear with the corresponding region on the Z-SLR and exhibits a prominent accumulation of repetitive elements, likely representing the earliest stages of recombination arrest. FS1 and FS2, in contrast, have diverged to a large extent leading to breakdown of synteny between Z- and W-haplotypes in these regions. Synteny breakdown between sex chromosomes has also been found in other studies with haplotype-resolved assemblies and likely reflects the retention of re-arrangements after recombination arrest rather than difficulties with assembling repeat-rich regions [18, 55, 57]. The W-SLR of *S. herbacea* has lost 23% of the conserved and ancestral genes, and the female-specific regions (FS1, FS2) within the W-SLR have accumulated nonsense variants and pseudogenes, providing clear evidence of sequence degeneration. Sequence divergence in the remaining ZW-homologous genes reached K_s_ values of 0.05 in FS2, higher than in other *Salix* species [7, 33]. *Salix herbacea* had a similar degree of gene loss and divergence (K_s_) as reported for the younger sex-linked regions in *Humulus* and *Cannabis* [59], *Rumex hastatulus* [18] and *Silene latifolia* [55, 56], as well as in primates [57], sticklebacks [21] and birds [17]. Phylogenetic analyses of single-copy orthologs among the ZW homologous genes suggest that divergence between the sex chromosomes of *S. herbacea* occurred mostly after the split from *S. purpurea,* which has been dated to nearly 14–20 million years before the present, using a phylogeny with fossil calibration [31]. This is a comparatively long divergence time, considering that the heteromorphic sex chromosomes of the white campion (*Silene latifolia*) have evolved within the last 11 million years [55]. However, divergence time estimates are difficult to compare as they depend on mutation rates and generation times. This is an issue particularly for S*. herbacea* since it relies on both clonal and sexual reproduction and is very long-lived with maximum age estimates exceeding 2000 years [60]. Nonetheless, our data suggests that sex chromosome evolution in the Salicaceae family involves considerable degeneration over extended periods, in accordance with the classic predictions for sex chromosome evolution [1].

### Sex-linked region includes conserved and newly recruited genes with putative sex-specific functions

Genes that were specific for the W-SLR assembly or had fixed female-specific polymorphisms in our study population included homologs to *A. thaliana* genes involved not only in flower development and female gametogenesis, but also in male-specific functions, such as anther and pollen development. Interestingly, all male-function genes had a conserved location on chromosome 15 across six *Salix* and *Populus* species, including species with male heterogamy. This finding suggests that these genes may have been present in the currently sex-linked region before female heterogamety evolved. Genes with putative functions in flower development were either ancestral, that is, shared with at least one other *Salix* or *Populus* species, or *S. herbacea* specific. Genes with putative function in female gametogenesis, in contrast, were mainly *S. herbacea* specific, suggesting that they were recruited from autosomes to the W-SLR more recently, consistent with sexually antagonistic selection [11–13]. Conserved genes also included W-SLR specific homologs to the *A. thaliana DEAD* genes with functions related to female meiosis. This finding is fascinating as *Salix* species, including *S. herbacea*, frequently have female-biased sex ratios that may arise through genetic rather than ecological mechanisms [61, 62]. In theoretical studies, sex ratio selection and meiotic drive have been shown to lead to sex chromosome turnover [63], offering another possible explanation for the processes behind the evolution of sex chromosomes in *Salix*.

### Candidate sex determination mechanism: *ARR17* replaced by regulation of downstream *PISTILLATA* by inverted repeats

We detected a conspicuous pattern for the gene *PISTILLATA* (*PI*) in *S. herbacea. PI* is a conserved multicopy gene essential for the production of male flowers in many species, including *Populus tremula* [27–30]. Full-length sequences of *PI* were located on two autosomes (chromosomes 2 and 5) and on the Z-SLR. *PI* pseudogenes were present in the FS1 and FS2 regions of the W-SLR in the form of seven pairs of partial (exon 1) *PI* copies arranged as inverted tandem repeats. This peculiar arrangement suggests the formation of double-stranded RNA hairpin structures that can produce small interfering RNA (siRNA) that silence coding RNAs by sequence complementarity [64, 65]. Such a suppression mechanism is prevalent in angiosperms [66] and has been suggested as a sex-determination mechanism in persimmon [28] and *Populus* [29]. Suppression of stamen development by interfering RNA targeting *PI* has further been demonstrated experimentally in legumes [50]. This raises the possibility that hairpin-like duplicates of *PI* on the W chromosome silence *PI* on autosomes and Z chromosomes in females, suppressing the development of male organs; however, functional studies are needed to further explore this finding.

Interestingly, the upstream sex determination gene *ARR17* in *Populus*, also suppresses *PI,* leading to the development of females [27, 29, 30]. In XY males of *P. tremula, ARR17* is itself suppressed by small RNAs generated from partial duplicates of *ARR17* on the Y chromosome. This double suppression mechanism has also been suggested for *Salix* species with XY sex determination [6, 7], but *ARR17* involvement in sex determination remains unclear for *Salix* species with ZW sex determination [67, 68]. In *S. herbacea*, we detected *ARR17* only on chromosome 19 and partial duplicates were not found. We did find another *RR* gene, *ARR16,* on the W-SLR; however, this gene was also present on autosomes (chr. 19), and there is no evidence that *ARR16* has the same function as *ARR17* in *Populus,* given the strong sequence divergence between *ARR16* and *ARR17*. If the autosomal *ARR17* in *S. herbacea* had the same function as in *P. tremula* [29], all individuals would experience *PI* suppression and develop as females; this mechanism is therefore unlikely. Instead, our findings suggest that the role of *ARR17* in regulating the downstream *PI* is superseded by *PI* silencing mediated by *PI* pseudogenes on the W-SLR, which leads to a suppression of male development in ZW females only.

We detected autosomal *PI* copies on chromosomes 2 and 5 in all *Salix* and *Populus* species assessed. Inverted partial duplicates of *PI*, however, were only found in the clade of *Salix* species with ZW sex determination but not in those with XY sex determination. Partial *PI* duplicates were also reported recently for the diploid-tetraploid species complex *S. polyclona* [69], that is phylogenetically close to the diploid ZW species included in our study and also has female heterogamety [70]. In *S. herbacea*, *PI* sex determination could thus be ancestral. An increase in the number of partial duplicate pairs to seven could, however, be species-specific. Conversely, autosomal *ARR17* genes together with potentially male-determining partial *ARR17* copies on the Y chromosomes were common in *Salix* species with XY sex determination, whereas only some of the ZW *Salix* species had partial *ARR17* copies. This leads us to the tentative suggestion that a shift from *ARR17*-controlled sex determination to *PI*-controlled sex determination could be associated with the transition between XY sex determination and ZW sex determination [31].

## Conclusions

Our study provides one of the few examples of the proximate mechanisms producing large sex-linked regions: a large inversion (5 Mb) on the W-chromosome of the dwarf willow *Salix herbacea* recently incorporated pseudoautosomal sequence into the sex-linked region, resulting in stepwise recombination suppression. Our results further suggest the involvement of conserved genetic pathways in sex determination across the closely related genera *Salix* and *Populus*, but with differences in the regulation mechanisms. Given our data, it is unlikely that the sex determination gene proposed for *Populus, ARR17* [29], a regulator of the male essential gene *PISTILLATA (PI),* is involved in the sex determination of *Salix* species with ZW sex determination. Instead, we propose the down-regulation of *PI* in females by W-specific pseudogenes of *PI* as a new candidate mechanism for sex determination. Our findings align well with results in animals where evolutionary changes in regulators of sex determining pathways are common, while sex determining pathways themselves were more conserved [22].

## Methods

### Plant material

In a natural population of *Salix herbacea* near Jakobshorn, Switzerland (46.7720N, 9.8554E, 2535 m above sea level), we collected one female plant (*F005*) to be used for whole genome sequencing and sampled leaves from 60 female and 60 male individuals. We cultivated individual *F005* under controlled conditions and collected fresh tender leaves continuously to gain enough material for whole genome sequencing. Leaves were immediately frozen in liquid nitrogen, and stored at −80℃. The leaf material of the population collection was dried with silica gel (VWR/Avantor, Switzerland). For transcriptome sequencing, we collected five fresh tissues from two further individuals cultivated from seeds of the same populations: leaf, stem, root, female catkin and male catkin.

### HiFi and Hi-C library construction and sequencing

High molecular weight DNA for PacBio HiFi sequencing was extracted from individual F005 using a modified protocol by Workman et al. [71]. The sequencing library for PacBio HiFi sequencing was constructed using the SMRTbell Express Template Prep Kit 2.0 according to the manufacturer's instructions (PacBio, USA) and sequenced on a PacBio Sequel II system at Uppsala Genome Center, National Genomics Infrastructure, Sweden.

The nuclei of the same plant individual used for long-read sequencing (*F005*) were extracted from the lysed cells using gradient density centrifugation, and digested with restriction endonucleases. Hi-C library preparation was performed after DNA cross-linking, restriction endonuclease digestion (*Hindlll*), end-repairing, end-ligation, DNA capturing and purification. The Hi-C library was constructed and sequenced on the Illumina NovaSeq 6000 platform by Biomarker Technologies (BMK) GmbH (China).

### Transcriptome sequencing and short-read sequencing of pools and individuals

The total RNA of five tissues was isolated using the RNeasy Kit (Qiagen, Hilden, Germany) and subsequently pooled based on RNA quantity for library construction. Concentration and RNA integrity were accessed using the Bioanalyzer Agilent 2100 (Agilent Technologies, Böblingen, Germany). One mRNA library was prepared with polyA enrichment and sequenced on NovaSeq PE150 by Novogene (UK).

The genomic DNA of 60 female and 60 male individuals of one population was isolated using the DNeasy Plant Mini Kit (Qiagen, Hilden, Germany). DNA concentration was measured using Quant-iT PicoGreen dsDNA kit (Thermo Fisher Scientific, Waltham, MA, USA) and a Fluorescence Microplate Reader (ThermoFisher, Waltham, MA, USA); the quality of DNA was assessed using a Nanodrop Spectrophotometer (Thermo Fisher Scientific, Waltham, MA, USA). Female and male pools with 50 individuals each were prepared based on equimolar DNA concentrations to create two pooled DNA libraries. Additionally, 20 individual libraries were prepared separately for ten females and ten males. In total, 22 Illumina TruSeq PCR-free libraries were constructed using unique dual indexes, targeting an insert size of 350 bp. The sequencing was performed on NovaSeq 6000 by SNP&SEQ Technology Platform in Uppsala and National Genomics Infrastructure in Stockholm (Sweden).

### Chromosome-scale assembly with HiFi and Hi-C data

For the female individual *F005*, high-quality HiFi Circular Consensus Sequencing (CCS) reads were used for an initial contig assembly using HiFiasm v0.15.4 [72] with default settings. We then purged the redundant haplotypic duplications using purge_dups v1.2.5 [73].

We filtered Hi-C reads using Fastp v0.23.1 [74] to remove the adapters and low quality reads (–qualified_quality_phred 20, –length_required 100), trim reads in front and tail (–cut_front –cut_tail –cut_window_size 4 –cut_mean_quality 20) and correct mismatched base pairs. The filtered HiC reads were mapped to the initial assembly by Juicer v1.6 [75] to generate HiC map bins. We followed 3D-DNA pipelines [76] to scaffold the genome and eliminate misjoins with three iterative steps. The output assembly of each step was visualized and evaluated through Juicebox [77]. The assembly from the early step was retained as the candidate ‘mega-scaffold’ with the least fragments and errors in breaking contigs. We manually curated the assembly to correct mis-joins, order, and orientation. The revised assembly was polished, split, sealed and merged in the post-process by 3D-DNA [76].

We applied the same processes to both haplotypes. In the end, we manually modified the order and orientation of the chromosomes by aligning both haplotypes to keep them parallel. The chromosome assignment was done according to the 19 chromosomes of *Salix purpurea*.

The final assembly was evaluated by mapping the HiFi reads and Illumina paired-end reads (see below), using Minimap v2 [78] and BWA v0.7.17 [79], respectively. BUSCO v5.0.0 [80] was used to assess the completeness of the genome assembly.

### Genome annotation

We identified repetitive sequences in the genome assembly using RepeatMasker v4.1.5 [81] based on repeat sequences registered in Repbase [82], species-specific repeat datasets from *Populus tricocarpa* [83], de novo repeat libraries built with RepeatModler v2.0.3 [84] and LTR_retriever v3.0.1 [85]. To further annotate unknown repeats, we adopted four to six rounds of self-classification and the database of *Arabidopsis thaliana* [86] by Repclassifier v1.1 [84]. Tandem repeats were predicted using trf v4.10.0 under the recommended parameters [87].

Based on the repeat-masked genome, gene prediction was performed following automated pipelines using BRAKER v3.0.4 [88] with GeneMark-ETP and AUGUSTUS for three separate rounds: homology-based only [89], RNA-seq only [90, 91], and combining homology-based and RNA-seq [92–96]. Transcriptome sequences from five tissues of *S. herbacea* were mapped to the genome assembly using HISAT2 v2.2.1 [97] and SAMtools v1.17 [98]. For the homology-based prediction, protein sequences of six species (*Arabidopsis thaliana*, *Salix purpurea*, *S. brachista*, *S. dunnii*, *Populus deltoides*, *P. trichocarpa*; [33, 83, 99–102], see Additional file 3: Table S4) were aligned to the assembly using BLAST v2.16.0 [103]. High-quality alignments were selected and integrated using TSEBRA v1.1.2.4 [104]. The function of the predicted genes was annotated using three strategies: 1) based on the homologous genes using EGGNOG-MAPPER [35]; 2) based on domain similarities using InterProScan v5.62–94.0 [105]; 3) based on sequence identity with the gene database of *Arabidopsis thaliana* using BLAST v2.16.0 [103].

### Genotype calling of females and males

Whole-genome short reads for both pools and individuals were preprocessed with Fastp v0.23.4 [74] using the following parameter settings: -q 20 -u 20 -l 100 -y 30 -g 10—× 10 -p 20, and quality control was performed using FastQC v0.11.9 [106]. We mapped clean paired-end reads to our genome assembly using BWA v0.7.17 [79]. The mapping output was filtered and sorted according to coordinates using SAMtools v1.17 [107]. Duplicate reads from sample preparation, amplification and sequencing were detected and marked using PicardTools v2.27.5 [108]. Variants of individual samples were called and genotyped using GATK v4.1.4.1 [109] and split into SNPs and INDELs. We applied hard filtering pipelines to SNPs (QD < 2.0, QUAL < 30.0, SOR > 3, FS > 60, MQ < 40, MQRankSum < −12.5, ReadPosRankSum < −8, DP < 30) and INDELs (FS > 200, QD < 2, QUAL < 30, ReadPosRankSum < −20, DP < 30) separately. High-quality SNPs were filtered using VCFtools v0.1.16 [110] with the criteria: MAF > 0.1, minDP = 10, maxDP = 100, max-missing = 1. For the pooled samples, the variants were called using SAMtools v1.17 [107] and PoPoolation2 [111] were then used to call SNPs with counts of at least 10 for the alternate allele and a depth between 50 and 1,000 × at the position concerned. All SNPs with coverage lower than 10 or higher than 500 were discarded.

### Identification of sex-linked regions

To identify sex-linked regions in *S. herbacea*, we utilized depth-based, genetic differentiation-based and heterozygosity-based approaches. Here, a higher mapping depth in one sex indicates sex-specific sequences on one of the haplotypes, especially if combined with high heterozygosity in the same sex. High genetic differentiation between sexes is expected in or near sex-linked, recombination-suppressed regions [58].

We calculated mapping depth in 10 kb windows with Mosdepth v0.3.3 [112] using bases with mapping quality over 20. After normalization with the overall mean depth in each pool, we calculated the proportion of female reads in all reads as F/(F + M). The same approach was applied in the individually resequenced individuals (10 females and 10 males). We calculated genetic differentiation as weighted Weir and Cockerham’s F_ST_ between sexes in 10 kb windows with 10 kb steps for female and male pools, as well as for individuals using VCFtools v0.1.16 [110, 113]. The proportion of heterozygous sites in 10 kb windows of each re-sequenced individual was calculated and averaged within the sexes.

In addition to the window-based analyses above, we also conducted a genome-wide search for sex-specific regions (present in one sex but not in the other) at single base pair resolution. For this analysis, we used BEDtools v2.31.1 [96] and the individual re-sequencing data from 10 individuals of each sex. We identified these regions as absent from all 10 individuals of one sex and present in all individuals of the other sex. To account for mis-mapping in the short-read data, we scored sites with up to 5 reads, corresponding to the lowest 20 percent of the depth distribution as absent. Female-specific sites were strictly defined as those properly mapped in all ten females and which showed no proper mapping in any of the ten males. Conversely, male-specific sites were identified based on the opposite criteria.

### Structural variation on chr. 15 in *S. herbacea*

We identified putative centromeric regions of both haplotypes based on sequence specificity, tandem repeats (TRs), and long terminal repeat retrotransposons (LTRs) of the genome assembly using CentIER v3.0 [114].

To detect structural variation between sex chromosomes, we aligned the two haplotypes from the genome assembly using the MUMmer v3.23 pipeline [115]. The maximum gap between two adjacent matches in a cluster was set to 500 bp, and the minimum length of a cluster was 100 bp. We subsequently applied hard filtering and kept only unique alignments with identity > 89% and alignment length > 1000 bp.

### Synteny analysis with other *Salix* species

To gain insight into the evolution of structural variation, we performed pairwise genome alignments between the two haplotypes of *Salix herbacea* and two published *Salix* genomes (*S. purpurea* and *S. arbutifolia*; [6, 33], Additional file 3: Table S4). We used Minimap2 v2.24 [78] allowing for sequence divergence of 0.5% (asm5). For structural variation calling, the alignment results were processed to SyRI v1.6.4 [116] with parameters set to “–tdmaxolp 0.5 –hdrseq –cigar –maxsize 100,000”.

We focused on inversion blocks, filtering out the ambiguous alignments. We excluded blocks with inverted regions covering less than 10% of their length while retaining syntenic regions or structural rearrangements within these blocks. Syntenic blocks between multiple genome assemblies were identified and visualized using Plotsr v0.5.5 [117].

### Estimation of LTR insertion times

LTR_harvest and LTR_finder were used to predict full-length LTR retrotransposons in the genome assembly [118, 119]. The insertion time of LTRs on the W-chromosome was estimated based on the substitutions between 5’ and 3’ flanking LTR repeats, assuming a mutation rate of 2.5 × 10^–9^ per generation [120].

### Pseudogenization

Using the annotation described above, we estimated the number and sequence proportion of genes along the two PARs, the SLR of haplotype 1 of chromosome 15, as well as the proportion of LTRs and pseudogenes.

We annotated pseudogenes using a modified version of the pipelines of Xie et al. [121], including 1) masking of repetitive sequences and coding regions of the reference genome; 2) identification of intergenic regions with sequence identity to the annotated proteins of the same haplotype using tBLASTn v2.16.0 [103]; 3) filtering of matches with identity < 40%, match length < 30 amino acids; 4) linking adjacent regions within 50 bp distance on the chromosome when matched to the same protein; 5) stacking homologous genes on the same location of the chromosome with at least 80% sequence overlap. In this process, a unique chromosome region can match with multiple genes.

### Evolutionary conservation and gene loss

We identified ZW homologous genes under a strict threshold (e-value > 1e^−10^, identity > 80 and alignment/query length > 70%) using BLAST v2.16.0 [103]. Z-specific and W-specific genes without homologs on the other haplotype were determined by excluding homologous genes under a soft threshold (e-value > 1e^−5^, identity > 60, alignment/query length > 50%).

Conserved genes were defined as Z-W homologs with orthologs with all three clades: ZW *Salix* species (*S. purpurea*, *S. suchowensis*), XY *Salix* species (*S. arbutifolia*, *S. dunnii*) and *Populus* species (*P. qiongdaoensis* and *P. trichocarpa*, Additional file 3: Table S4) as detected by OrthoFinder v2.5.2 [122]. Ancestral genes were defined as Z-W homologs with orthologs in at least one of these six species. The proportion of conserved or ancestral genes lost on each haplotype was calculated as (1–the number of conserved orancestral genes on a haplotype divided by the joint number of conserved orancestral genes across both haplotypes).

### Degeneration in the coding-sequences

We concatenated SNPs and INDELs for the ten individuals of each sex, using conservatively filtered SNPs with the following parameters in VCFtools v0.1.16 [110]: minDP = 10, maxDP = 100, MAF = 0.1, max-missing = 0.8, minQ = 30. We categorized the effect of these variants within coding regions into three groups using SnpEff v5.2 [123]: 1) nonsense; the variant is expected to change a codon into a stop codon; 2) missense; the variant may change protein effectiveness; 3) synonymous; the variant is not expected to change the protein.

### Divergence between Z- and W-haplotypes

We estimated synonymous site divergence rates (K_s_) and and the ratio between nonsynonymous and synonymous divergence rates (K_a_/K_s_) of homologous genes between the Z- and W-haplotypes based on the YN method [124] using KaKs_caculator v2.0 [125]. Protein-coding sequences of ZW homologous genes were extracted using amino acid alignments with ParaAT [126]. To ensure accurate alignments, ambiguous sites and gaps were removed, sequences with alignment coverage below 60% were excluded and only aligned nucleotide regions longer than 300 bp were retained.

To reconstruct phylogenetic relationships among Z-W homologous genes we identified single-copy orthologs in *S. herbacea*, *S. purpurea* and *P. trichocarpa* using OrthoFinder v2.5.2 [122]. A maximum likelihood tree for each gene was built using IQ-TREE v2.2.2 [127] with the best model and partition scheme selected by ModelFinder [128]. A topological distance matrix between phylogenetic trees was generated in R with the APE package [129] in R v4.4.0 [130] with *P. trichocarpa* as an outgroup. We performed hierarchical cluster analysis with the UPGMA method and generated consensus trees for gene trees bootstrap support for nodes > 80%.

To infer the phylogenetic position of *S. herbacea*, we downloaded eight published *Salix* genomes (*S. purpurea*, *S. udensis* (ZZ), *S. suchowensis*, *S. koriyanagi*, *S. brachista*, *S. viminalis*, *S. dunnii* and *S. arbutifolia*; [6, 33, 100, 101, 131–133]) and two *Populus* genomes (*P. trichocarpa*, *P. qiongdaoensis*; [51, 83], Additional file 3: Table S4). Gene trees were constructed using the pipeline described above. Genes from chromosome 7, 15 and 19 that have been identified as sex chromosomes were excluded. A species tree was inferred from gene trees using ASTRAL [134]. We estimated divergence times among different species using MCMCtree in the PAML v4.10.7 package [135] under the parameters “clock = 2, model = 4, BDparas = 1 1 0.1, kappa_gamma = 6 2, alpha_gamma = 1 1, rgene_gamma = 10 25 1, sigma2_gamma = 1 10”. We used fossil calibration information as 48–52 million years for the separation of *Salix* and *Populus* clades [136], and at least 23 million years for the formation of the subgenus *Vetrix* in the late Oligocene [137].

### Genes present only on the W-SLR or only on the Z-SLR

We analysed whether the W- or Z-specific genes detected above were completely lost or degenerated in the other haplotype. We identified gene residues (pseudogenes) retained on the W-haplotype using Z-specific genes as the query after masking out coding and repetitive sequences. The partial copies were determined as sequences with sequence identities with the conserved exon > 80%. We applied the same method to pseudogenes on Z-haplotype.

### Genes with female-specific polymorphisms

We annotated the female-specific regions identified above using our gene annotation of the reference genome. Regions without hits were scanned for pseudogenization using the pseudogene pipeline described above. Putative functions of these genes were inferred from previous studies in plants [36–42, 44–47, 49, 138–156]. Then, we searched for fixed female-specific single-nucleotide variants (SNPs) that were consistently heterozygous in all ten females but homozygous in all ten males. To reduce the effects of duplication and multiple alignments in the sex-linked region (SLR), individuals were scored conservatively as heterozygous when the proportion of reads of the minor allele was greater than 0.3 (the ratio of minor allele and major allele > 0.43). We further restricted the identification of female-specific single-nucleotide variants (ZW in females and ZZ in males) to biallelic loci, where males were homozygous for one of the two alleles found in females. We annotated these loci using the repeat and gene annotations described above. We further evaluated the impact of the fixed female-specific single-nucleotide variants on gene function using SnpEff v5.2 [123].

### Evolutionary history of genes with sex-specific variation

For candidate genes identified in *S. herbacea*, we searched for the homologous genes in 6 genomes of *Salix* and *Populus* (*S. purpurea*, *S. suchowensis*, *S. arbutifolia*, *S. dunnii*, *P. qiongdaoensis* and *P. trichocarpa*) using BLASTp v2.16.0 [103]. We generated multiple synteny plots for genes using MCScan (Python version) under JCVI [157, 158].

To identify whether female-specific sites (INDELs) and female-specific single-nucleotide variants (SNPs) were also present in other *Salix* species, we mapped the Illumina reads of both sexes of *S. purpurea*, *S. suchowensis*, *S. viminalis* (for data sources see Additional file 3: Table S4) to the *S. herbacea* reference genome using BWA v0.7.17 [79]. The mapping output and genotype calling were filtered following the process described above.

### Candidate sex determination genes

To investigate the previously identified candidate sex determination genes, *ARR17* [29]*,* in *Populus* and *Salix*, we used BLASTp v2.16.0 [103] searches for intact homologous genes in *S. herbacea* and the other eight *Salix* and two *Populus* genome assemblies (for data sources see Additional file 3: Table S4) using *ARR17* (Potri.019G133600) as the query. To detect partial duplicates, *ARR17* segmental sequences and five complete exon nucleotide sequences from Hu et al. [68] were searched in all genome assemblies using BLASTN v2.16.0 [103] with “-evalue 1e-5 -word_size 8”. Genes with sequence identity over 80% in all exons were considered complete genes and sequences matching only on the first exon were considered partial duplicates. The same workflow was applied to the candidate gene (*PI*) proposed in this study. The first exon of all complete and partial duplicate copies was aligned by MUSCLE v5.1 [159]. Phylogenies of *PI* sequences were constructed using maximum likelihood analyses in IQ-TREE v2.2.2 [127] with the best model and partition scheme selected by ModelFinder [128].

## Supplementary Information

Additional file 1: Figures S1-9.

Additional file 2: Figures S10-16.

Additional file 3: Tables S1-9.

Additional file 4: Dataset S1. Summary of female-specific single-nucleotide variants on the W-haplotype of *Salix herbacea*.

Additional file 5: Dataset S2. Summary of female-specific sites on the W-haplotype of *Salix herbacea*.

Additional file 6: Dataset S3. Blast result of *ARR17* gene in 10 genome assemblies of *Salix* species.

## Data Availability

Data supporting the findings of this work are available within the paper and associated Supplementary Information files. The whole-genome assembly project for Salix herbacea is deposited in GenBank under the BioProject accessions PRJNA1199913. The genome assembly and annotation are also available on Zenodo [160]. All raw reads of *Salix herbacea* generated in this study are deposited in the NCBI database under BioProject accession PRJNA1196844. Computer code and pipelines needed to reproduce the analysis and figures in this article are available both on GitHub [161] and Zenodo [162].

## References

[1] Bachtrog D. Y-chromosome evolution: emerging insights into processes of Y-chromosome degeneration. Nat Rev Genet. 2013;14:113–24. 23329112 10.1038/nrg3366 PMC4120474

[2] Beukeboom L, Perrin N. The evolution of sex determination. London, England: Oxford University Press; 2014.

[3] Jeffries DL, Lavanchy G, Sermier R, Sredl MJ, Miura I, Borzée A, et al. A rapid rate of sex-chromosome turnover and non-random transitions in true frogs. Nat Commun. 2018;9:4088. 30291233 10.1038/s41467-018-06517-2 PMC6173717

[4] El Taher A, Ronco F, Matschiner M, Salzburger W, Böhne A. Dynamics of sex chromosome evolution in a rapid radiation of cichlid fishes. Sci Adv. 2021;7:eabe8215. 34516923 10.1126/sciadv.abe8215 PMC8442896

[5] Myosho T, Otake H, Masuyama H, Matsuda M, Kuroki Y, Fujiyama A, et al. Tracing the emergence of a novel sex-determining gene in medaka. Genetics. 2012;191:163–70. 22367037 10.1534/genetics.111.137497 PMC3338257

[6] Wang Y, Gong G-N, Wang Y, Zhang R-G, Hörandl E, Zhang Z-X, et al. Gap-free X and Y chromosome assemblies of *Salix arbutifolia* reveal an evolutionary change from male to female heterogamety in willows, without a change in the position of the sex-determining locus. New Phytol. 2024. 10.1111/nph.19744 38581199

[7] Wang D, Li Y, Li M, Yang W, Ma X, Zhang L, et al. Repeated turnovers keep sex chromosomes young in willows. Genome Biol. 2022;23:200. 36151581 10.1186/s13059-022-02769-w PMC9502649

[8] Yang W, Wang D, Li Y, Zhang Z, Tong S, Li M, et al. A general model to explain repeated turnovers of sex determination in the Salicaceae. Mol Biol Evol. 2021;38:968–80. 33027519 10.1093/molbev/msaa261 PMC7947767

[9] Yazdi HP, Ellegren H. Old but not (so) degenerated--slow evolution of largely homomorphic sex chromosomes in ratites. Mol Biol Evol. 2014;31:1444–53. 24618361 10.1093/molbev/msu101

[10] Kuhl H, Guiguen Y, Höhne C, Kreuz E, Du K, Klopp C, et al. A 180 Myr-old female-specific genome region in sturgeon reveals the oldest known vertebrate sex determining system with undifferentiated sex chromosomes. Philos Trans R Soc Lond B Biol Sci. 2021;376:20200089. 34247507 10.1098/rstb.2020.0089 PMC8273502

[11] Saunders PA, Muyle A. Sex chromosome evolution: hallmarks and question marks. Mol Biol Evol. 2024;41:msae218. 39417444 10.1093/molbev/msae218 PMC11542634

[12] Jay P, Jeffries D, Hartmann FE, Véber A, Giraud T. Why do sex chromosomes progressively lose recombination? Trends Genet. 2024;40:564–79. 38677904 10.1016/j.tig.2024.03.005

[13] Rice WR. The accumulation of sexually antagonistic genes as a selective agent promoting the evolution of reduced recombination between primitive sex chromosomes. Evolution. 1987;41:911–4. 28564364 10.1111/j.1558-5646.1987.tb05864.x

[14] Lenormand T, Roze D. Y recombination arrest and degeneration in the absence of sexual dimorphism. Science. 2022;375:663–6. 35143289 10.1126/science.abj1813

[15] Olito C, Ponnikas S, Hansson B, Abbott JK. Consequences of partially recessive deleterious genetic variation for the evolution of inversions suppressing recombination between sex chromosomes1. Evolution. 2024;78:1499–510. 38853722 10.1093/evolut/qpae060 PMC12372689

[16] Jeffries DL, Gerchen JF, Scharmann M, Pannell JR. A neutral model for the loss of recombination on sex chromosomes. Philos Trans R Soc Lond B Biol Sci. 2021;376:20200096. 34247504 10.1098/rstb.2020.0096 PMC8273504

[17] Sigeman H, Downing PA, Zhang H, Hansson B. The rate of W chromosome degeneration across multiple avian neo-sex chromosomes. Sci Rep. 2024;14:16548. 39020011 10.1038/s41598-024-66470-7 PMC11255319

[18] Sacchi B, Humphries Z, Kružlicová J, Bodláková M, Pyne C, Choudhury BI, et al. Phased assembly of Neo-sex chromosomes reveals extensive Y degeneration and rapid genome evolution in *Rumex hastatulus*. Mol Biol Evol. 2024;41:msae074. 38606901 10.1093/molbev/msae074 PMC11057207

[19] Xu L, Ren Y, Wu J, Cui T, Dong R, Huang C, et al. Evolution and expression patterns of the neo-sex chromosomes of the crested ibis. Nat Commun. 2024;15:1670. 38395916 10.1038/s41467-024-46052-x PMC10891136

[20] Lemaitre C, Braga MDV, Gautier C, Sagot M-F, Tannier E, Marais GAB. Footprints of inversions at present and past pseudoautosomal boundaries in human sex chromosomes. Genome Biol Evol. 2009;1:56–66. 20333177 10.1093/gbe/evp006 PMC2817401

[21] Peichel CL, McCann SR, Ross JA, Naftaly AFS, Urton JR, Cech JN, et al. Assembly of the threespine stickleback Y chromosome reveals convergent signatures of sex chromosome evolution. Genome Biol. 2020;21:177. 32684159 10.1186/s13059-020-02097-x PMC7368989

[22] Zhu Z, Younas L, Zhou Q. Evolution and regulation of animal sex chromosomes. Nat Rev Genet. 2024;26:59–74. 10.1038/s41576-024-00757-3 39026082

[23] Wilkins AS. Moving up the hierarchy: a hypothesis on the evolution of a genetic sex determination pathway. BioEssays. 1995;17:71–7. 7702596 10.1002/bies.950170113

[24] Feng G, Sanderson BJ, Keefover-Ring K, Liu J, Ma T, Yin T, et al. Pathways to sex determination in plants: how many roads lead to Rome? Curr Opin Plant Biol. 2020;54:61–8. 32106015 10.1016/j.pbi.2020.01.004

[25] Harkess A, Huang K, van der Hulst R, Tissen B, Caplan JL, Koppula A, et al. Sex determination by two Y-linked genes in garden asparagus. Plant Cell. 2020;32:1790–6. 32220850 10.1105/tpc.19.00859 PMC7268802

[26] Akagi T, Pilkington SM, Varkonyi-Gasic E, Henry IM, Sugano SS, Sonoda M, et al. Two Y-chromosome-encoded genes determine sex in kiwifruit. Nat Plants. 2019;5:801–9. 31383971 10.1038/s41477-019-0489-6

[27] Leite Montalvão AP, Kersten B, Fladung M, Müller NA. The diversity and dynamics of sex determination in dioecious plants. Front Plant Sci. 2020;11:580488. 33519840 10.3389/fpls.2020.580488 PMC7843427

[28] Akagi T, Henry IM, Tao R, Comai L. Plant genetics. A Y-chromosome-encoded small RNA acts as a sex determinant in persimmons. Science. 2014;346:646–50. 25359977 10.1126/science.1257225

[29] Müller NA, Kersten B, Leite Montalvão AP, Mähler N, Bernhardsson C, Bräutigam K, et al. A single gene underlies the dynamic evolution of poplar sex determination. Nat Plants. 2020;6:630–7. 32483326 10.1038/s41477-020-0672-9

[30] Leite Montalvão AP, Kersten B, Kim G, Fladung M, Müller NA. ARR17 controls dioecy in *Populus* by repressing B-class MADS-box gene expression. Philos Trans R Soc Lond B Biol Sci. 2022;377:20210217. 35306887 10.1098/rstb.2021.0217 PMC8935312

[31] Ogutcen E, de Lima FP, Wagner ND, Marinček P, Vir Leong J, Aubona G, et al. Phylogenetic insights into the Salicaceae: The evolution of willows and beyond. Mol Phylogenet Evol. 2024;199:108161. 39079595 10.1016/j.ympev.2024.108161

[32] Hyden B, Carper DL, Abraham PE, Yuan G, Yao T, Baumgart L, et al. Functional analysis of *Salix purpurea* genes support roles for ARR17 and GATA15 as master regulators of sex determination. Plant Direct. 2023;7:e3546. 38028649 10.1002/pld3.546 PMC10651977

[33] Zhou R, Macaya-Sanz D, Carlson CH, Schmutz J, Jenkins JW, Kudrna D, et al. A willow sex chromosome reveals convergent evolution of complex palindromic repeats. Genome Biol. 2020;21:38. 32059685 10.1186/s13059-020-1952-4 PMC7023750

[34] Almeida P, Proux-Wera E, Churcher A, Soler L, Dainat J, Pucholt P, et al. Genome assembly of the basket willow, *Salix viminalis*, reveals earliest stages of sex chromosome expansion. BMC Biol. 2020;18:78. 32605573 10.1186/s12915-020-00808-1 PMC7329446

[35] Cantalapiedra CP, Hernández-Plaza A, Letunic I, Bork P, Huerta-Cepas J. EggNOG-mapper v2: Functional annotation, orthology assignments, and domain prediction at the metagenomic scale. bioRxiv. bioRxiv; 2021. 10.1093/molbev/msab293 PMC8662613 34597405

[36] Yang Y, Ma C, Xu Y, Wei Q, Imtiaz M, Lan H, et al. A zinc finger protein regulates flowering time and abiotic stress tolerance in chrysanthemum by modulating gibberellin biosynthesis. Plant Cell. 2014;26:2038–54. 24858937 10.1105/tpc.114.124867 PMC4079367

[37] Swinka C, Hellmann E, Zwack P, Banda R, Rashotte AM, Heyl A. Cytokinin response factor 9 represses cytokinin responses in flower development. Int J Mol Sci. 2023;24:4380. 36901811 10.3390/ijms24054380 PMC10002603

[38] Kandasamy MK, Deal RB, McKinney EC, Meagher RB. Silencing the nuclear actin-related protein AtARP4 in *Arabidopsis* has multiple effects on plant development, including early flowering and delayed floral senescence: role of AtARP4 in plant development. Plant J. 2005;41:845–58. 15743449 10.1111/j.1365-313X.2005.02345.x

[39] Zhou D, Chen C, Jin Z, Chen J, Lin S, Lyu T, et al. Transcript profiling analysis and ncRNAs’ identification of male-sterile systems of *Brassica campestris* reveal new insights into the mechanism underlying anther and pollen development. Front Plant Sci. 2022;13:806865. 35211139 10.3389/fpls.2022.806865 PMC8861278

[40] Ramming A, Kappel C, Kanaoka MM, Higashiyama T, Lenhard M. Poly(A) polymerase 1 contributes to competence acquisition of pollen tubes growing through the style in *Arabidopsis thaliana*. Plant J. 2023;114:651–67. 36811355 10.1111/tpj.16162

[41] Mashiguchi K, Asami T, Suzuki Y. Genome-wide identification, structure and expression studies, and mutant collection of 22 early nodulin-like protein genes in *Arabidopsis*. Biosci Biotechnol Biochem. 2009;73:2452–9. 19897921 10.1271/bbb.90407

[42] Xia C, Wang Y-J, Liang Y, Niu Q-K, Tan X-Y, Chu L-C, et al. The ARID-HMG DNA-binding protein AtHMGB15 is required for pollen tube growth in *Arabidopsis thaliana*. Plant J. 2014;79:741–56. 24923357 10.1111/tpj.12582

[43] Pagnussat GC, Yu H-J, Ngo QA, Rajani S, Mayalagu S, Johnson CS, et al. Genetic and molecular identification of genes required for female gametophyte development and function in *Arabidopsis*. Development. 2005;132:603–14. 15634699 10.1242/dev.01595

[44] Harscoët E, Dubreucq B, Palauqui J-C, Lepiniec L. NOF1 encodes an *Arabidopsis* protein involved in the control of rRNA expression. PLoS ONE. 2010;5:e12829. 20877469 10.1371/journal.pone.0012829 PMC2942902

[45] Falbel TG, Koch LM, Nadeau JA, Segui-Simarro JM, Sack FD, Bednarek SY. SCD1 is required for cytokinesis and polarized cell expansion in *Arabidopsis thaliana*. Development. 2003;130:4011–24. 12874123 10.1242/dev.00619

[46] Veyres N, Danon A, Aono M, Galliot S, Karibasappa YB, Diet A, et al. The *Arabidopsis* sweetie mutant is affected in carbohydrate metabolism and defective in the control of growth, development and senescence. Plant J. 2008;55:665–86. 18452589 10.1111/j.1365-313X.2008.03541.x

[47] Graeff M, Straub D, Eguen T, Dolde U, Rodrigues V, Brandt R, et al. MicroProtein-mediated recruitment of CONSTANS into a TOPLESS trimeric complex represses flowering in *Arabidopsis*. PLoS Genet. 2016;12:e1005959. 27015278 10.1371/journal.pgen.1005959 PMC4807768

[48] Atanasov V, Schumacher J, Muiño JM, Larasati C, Wang L, Kaufmann K, et al. *Arabidopsis* BBX14 is involved in high light acclimation and seedling development. Plant J. 2024;118:141–58. 38128030 10.1111/tpj.16597

[49] D’Agostino IB, Deruère J, Kieber JJ. Characterization of the response of the *Arabidopsis* response regulator gene family to cytokinin. Plant Physiol. 2000;124:1706–17. 11115887 10.1104/pp.124.4.1706 PMC59868

[50] Benlloch R, Roque E, Ferrándiz C, Cosson V, Caballero T, Penmetsa RV, et al. Analysis of B function in legumes: PISTILLATA proteins do not require the PI motif for floral organ development in *Medicago truncatula*. Plant J. 2009;60:102–11. 19500303 10.1111/j.1365-313X.2009.03939.x

[51] Li Y, Wang D, Wang W, Yang W, Gao J, Zhang W, et al. A chromosome-level *Populus qiongdaoensis* genome assembly provides insights into tropical adaptation and a cryptic turnover of sex determination. Mol Ecol. 2023;32:1366–80. 35712997 10.1111/mec.16566

[52] Kersten B, Pakull B, Groppe K, Lueneburg J, Fladung M. The sex-linked region in *Populus tremuloides* turesson 141 corresponds to a pericentromeric region of about two million base pairs on *P. trichocarpa* chromosome 19. Plant Biol. 2014;16:411–8. 23710995 10.1111/plb.12048

[53] Zhang S, Wu Z, Ma D, Zhai J, Han X, Jiang Z, et al. Chromosome-scale assemblies of the male and female *Populus euphratica* genomes reveal the molecular basis of sex determination and sexual dimorphism. Commun Biol. 2022;5:1186. 36333427 10.1038/s42003-022-04145-7 PMC9636151

[54] Geraldes A, Hefer CA, Capron A, Kolosova N, Martinez‐Nuñez F, Soolanayakanahally RY, et al. Recent Y chromosome divergence despite ancient origin of dioecy in poplars (*Populus*). Mol Ecol. 2015;24:3243–56. 25728270 10.1111/mec.13126

[55] Moraga C, Branco C, Rougemont Q, Jedlička P, Mendoza-Galindo E, Veltsos P, et al. The *Silene latifolia* genome and its giant Y chromosome. Science. 2025;387:630–6. 39913565 10.1126/science.adj7430 PMC11890086

[56] Akagi T, Fujita N, Shirasawa K, Tanaka H, Nagaki K, Masuda K, et al. Rapid and dynamic evolution of a giant Y chromosome in *Silene latifolia*. Science. 2025;387:637–43. 39913598 10.1126/science.adk9074

[57] Zhou Y, Zhan X, Jin J, Zhou L, Bergman J, Li X, et al. Eighty million years of rapid evolution of the primate Y chromosome. Nat Ecol Evol. 2023;7:1114–30. 37268856 10.1038/s41559-022-01974-x

[58] Renner SS, Müller NA. Plant sex chromosomes defy evolutionary models of expanding recombination suppression and genetic degeneration. Nat Plants. 2021;7:392–402. 33782581 10.1038/s41477-021-00884-3

[59] Prentout D, Stajner N, Cerenak A, Tricou T, Brochier-Armanet C, Jakse J, et al. Plant genera *Cannabis* and *Humulus* share the same pair of well-differentiated sex chromosomes. New Phytol. 2021;231:1599–611. 33978992 10.1111/nph.17456

[60] Centenaro G, Petraglia A, Carbognani M, Piotti A, Hudek C, Büntgen U, et al. The oldest known clones of *Salix herbacea* growing in the Northern Apennines, Italy are at least 2000 years old. Am J Bot. 2023:e16243. 10.1002/ajb2.16243 37755870

[61] Mao X, Cortés AJ, Rixen C, Karrenberg S. Female‐biased population sex ratios caused by genetic rather than ecological mechanisms in dwarf willow (*Salix herbacea* L.). J Ecol. 2024;112:1731–42.

[62] Myers-Smith IH, Hik DS. Uniform female-biased sex ratios in alpine willows. Am J Bot. 2012;99:1243–8. 22763353 10.3732/ajb.1200107

[63] Scott MF, Osmond MM, Otto SP. Haploid selection, sex ratio bias, and transitions between sex-determining systems. PLoS Biol. 2018;16:e2005609. 29940019 10.1371/journal.pbio.2005609 PMC6042799

[64] Borges F, Martienssen RA. The expanding world of small RNAs in plants. Nat Rev Mol Cell Biol. 2015;16:727–41. 26530390 10.1038/nrm4085 PMC4948178

[65] Slotkin RK, Freeling M, Lisch D. Heritable transposon silencing initiated by a naturally occurring transposon inverted duplication. Nat Genet. 2005;37:641–4. 15908951 10.1038/ng1576

[66] Erdmann RM, Picard CL. RNA-directed DNA methylation. PLoS Genet. 2020;16:e1009034. 33031395 10.1371/journal.pgen.1009034 PMC7544125

[67] Hyden B, Carlson CH, Gouker FE, Schmutz J, Barry K, Lipzen A, et al. Integrative genomics reveals paths to sex dimorphism in *Salix purpurea* L. Hortic Res. 2021;8:170. 34333534 10.1038/s41438-021-00606-y PMC8325687

[68] Hu N, Sanderson BJ, Guo M, Feng G, Gambhir D, Hale H, et al. Evolution of a ZW sex chromosome system in willows. Nat Commun. 2023;14:7144. 37932261 10.1038/s41467-023-42880-5 PMC10628195

[69] Wang Y, Xue Z, Zhang R-G, Horandl E, Wang X-R, Mank JE, et al. A novel downstream factor in willows replaces the ancestral sex determining gene. bioRxiv. 2024. p. 2024.10.14.618180.

[70] Gulyaev S, Cai X-J, Guo F-Y, Kikuchi S, Applequist WL, Zhang Z-X, et al. The phylogeny of *Salix* revealed by whole genome re-sequencing suggests different sex-determination systems in major groups of the genus. Ann Bot. 2022;129:485–98. 35134824 10.1093/aob/mcac012 PMC8944726

[71] Workman R, Fedak R, Kilburn D, Hao S, Liu K, Timp W. High molecular weight DNA extraction from recalcitrant plant species for third generation sequencing. Protocols.io. 2019.

[72] Cheng H, Concepcion GT, Feng X, Zhang H, Li H. Haplotype-resolved de novo assembly using phased assembly graphs with hifiasm. Nat Methods. 2021;18:170–5. 33526886 10.1038/s41592-020-01056-5 PMC7961889

[73] Guan D, McCarthy SA, Wood J, Howe K, Wang Y, Durbin R. Identifying and removing haplotypic duplication in primary genome assemblies. Bioinformatics. 2020;36:2896–8. 31971576 10.1093/bioinformatics/btaa025 PMC7203741

[74] Chen S. Ultrafast one-pass FASTQ data preprocessing, quality control, and deduplication using fastp. Imeta. 2023;2:e107. 38868435 10.1002/imt2.107 PMC10989850

[75] Durand NC, Shamim MS, Machol I, Rao SSP, Huntley MH, Lander ES, et al. Juicer provides a one-click system for analyzing loop-resolution Hi-C experiments. Cell Syst. 2016;3:95–8. 27467249 10.1016/j.cels.2016.07.002 PMC5846465

[76] Dudchenko O, Batra SS, Omer AD, Nyquist SK, Hoeger M, Durand NC, et al. De novo assembly of the *Aedes aegypti* genome using Hi-C yields chromosome-length scaffolds. Science. 2017;356:92–5. 28336562 10.1126/science.aal3327 PMC5635820

[77] Robinson JT, Turner D, Durand NC, Thorvaldsdóttir H, Mesirov JP, Aiden EL. Juicebox.js provides a cloud-based visualization system for Hi-C data. Cell Syst. 2018;6:256-8.e1. 29428417 10.1016/j.cels.2018.01.001 PMC6047755

[78] Li H. New strategies to improve minimap2 alignment accuracy. Bioinformatics. 2021;37:4572–4. 34623391 10.1093/bioinformatics/btab705 PMC8652018

[79] Li H, Durbin R. Fast and accurate short read alignment with Burrows-Wheeler transform. Bioinformatics. 2009;25:1754–60. 19451168 10.1093/bioinformatics/btp324 PMC2705234

[80] Manni M, Berkeley MR, Seppey M, Simão FA, Zdobnov EM. BUSCO update: novel and streamlined workflows along with broader and deeper phylogenetic coverage for scoring of eukaryotic, prokaryotic, and viral genomes. Mol Biol Evol. 2021;38:4647–54. 34320186 10.1093/molbev/msab199 PMC8476166

[81] Tarailo-Graovac M, Chen N. Using RepeatMasker to identify repetitive elements in genomic sequences. Curr Protoc Bioinformatics. 2009;Chapter 4:4.10.1–4.10.14. 10.1002/0471250953.bi0410s25 19274634

[82] Bao W, Kojima KK, Kohany O. Repbase update, a database of repetitive elements in eukaryotic genomes. Mob DNA. 2015;6:11. 26045719 10.1186/s13100-015-0041-9 PMC4455052

[83] Tuskan GA, Difazio S, Jansson S, Bohlmann J, Grigoriev I, Hellsten U, et al. The genome of black cottonwood, *Populus trichocarpa* (Torr & Gray). Science. 2006;313:1596–604. 16973872 10.1126/science.1128691

[84] Flynn JM, Hubley R, Goubert C, Rosen J, Clark AG, Feschotte C, et al. RepeatModeler2 for automated genomic discovery of transposable element families. Proc Natl Acad Sci U S A. 2020;117:9451–7. 32300014 10.1073/pnas.1921046117 PMC7196820

[85] Ou S, Jiang N. LTR_retriever: a highly accurate and sensitive program for identification of long terminal repeat retrotransposons. Plant Physiol. 2018;176:1410–22. 29233850 10.1104/pp.17.01310 PMC5813529

[86] Quesneville H. Twenty years of transposable element analysis in the *Arabidopsis thaliana* genome. Mob DNA. 2020;11:28. 32742313 10.1186/s13100-020-00223-x PMC7385966

[87] Benson G. Tandem repeats finder: a program to analyze DNA sequences. Nucleic Acids Res. 1999;27:573–80. 9862982 10.1093/nar/27.2.573 PMC148217

[88] Stanke M, Diekhans M, Baertsch R, Haussler D. Using native and syntenically mapped cDNA alignments to improve *de novo* gene finding. Bioinformatics. 2008;24:637–44. 18218656 10.1093/bioinformatics/btn013

[89] Brůna T, Hoff KJ, Lomsadze A, Stanke M, Borodovsky M. BRAKER2: automatic eukaryotic genome annotation with GeneMark-EP+ and AUGUSTUS supported by a protein database. NAR Genom Bioinform. 2021;3:lqaa108. 33575650 10.1093/nargab/lqaa108 PMC7787252

[90] Hoff KJ, Lange S, Lomsadze A, Borodovsky M, Stanke M. BRAKER1: unsupervised RNA-seq-based genome annotation with GeneMark-ET and Augustus. Bioinformatics. 2016;32:767–9. 26559507 10.1093/bioinformatics/btv661 PMC6078167

[91] Lomsadze A, Burns PD, Borodovsky M. Integration of mapped RNA-Seq reads into automatic training of eukaryotic gene finding algorithm. Nucleic Acids Res. 2014;42:e119. 24990371 10.1093/nar/gku557 PMC4150757

[92] Gabriel L, Brůna T, Hoff KJ, Ebel M, Lomsadze A, Borodovsky M, et al. BRAKER3: Fully automated genome annotation using RNA-seq and protein evidence with GeneMark-ETP, AUGUSTUS and TSEBRA. bioRxiv. 2024. 10.1101/gr.278090.123 PMC11216308 38866550

[93] Bruna T, Lomsadze A, Borodovsky M. A new gene finding tool GeneMark-ETP significantly improves the accuracy of automatic annotation of large eukaryotic genomes. bioRxiv. 2024. 10.1101/gr.278373.123 PMC11216313 38866548

[94] Kovaka S, Zimin AV, Pertea GM, Razaghi R, Salzberg SL, Pertea M. Transcriptome assembly from long-read RNA-seq alignments with StringTie2. Genome Biol. 2019;20:278. 31842956 10.1186/s13059-019-1910-1 PMC6912988

[95] Pertea G, Pertea M. GFF utilities: GffRead and GffCompare. F1000Res. 2020. 10.12688/f1000research.23297.1. 32489650 10.12688/f1000research.23297.1 PMC7222033

[96] Quinlan AR. BEDTools: the Swiss-army tool for genome feature analysis. Curr Protoc Bioinformatics. 2014;47:11.12.1-34. 25199790 10.1002/0471250953.bi1112s47 PMC4213956

[97] Kim D, Paggi JM, Park C, Bennett C, Salzberg SL. Graph-based genome alignment and genotyping with HISAT2 and HISAT-genotype. Nat Biotechnol. 2019;37:907–15. 31375807 10.1038/s41587-019-0201-4 PMC7605509

[98] Li H, Handsaker B, Wysoker A, Fennell T, Ruan J, Homer N, et al. The sequence alignment/map format and SAMtools. Bioinformatics. 2009;25:2078–9. 19505943 10.1093/bioinformatics/btp352 PMC2723002

[99] Cheng C, Krishnakumar V, Chan AP, Thibaud‐Nissen F, Schobel S, Town CD. Araport11: a complete reannotation of the *Arabidopsis thaliana* reference genome. Plant J. 2017;89:789–804. 27862469 10.1111/tpj.13415

[100] Chen J-H, Huang Y, Brachi B, Yun Q-Z, Zhang W, Lu W, et al. Genome-wide analysis of cushion willow provides insights into alpine plant divergence in a biodiversity hotspot. Nat Commun. 2019;10:5230. 31745089 10.1038/s41467-019-13128-y PMC6864086

[101] He L, Jia K, Zhang R, Wang Y, Shi T, Li Z, et al. Chromosome-scale assembly of the genome of *Salix dunnii* reveals a male-heterogametic sex determination system on chromosome 7. Mol Ecol Resour. 2021;21:1966–82. 33609314 10.1111/1755-0998.13362 PMC8359994

[102] Xue L, Wu H, Chen Y, Li X, Hou J, Lu J, et al. Evidences for a role of two Y-specific genes in sex determination in *Populus deltoides*. Nat Commun. 2020;11:5893. 33208755 10.1038/s41467-020-19559-2 PMC7674411

[103] Camacho C, Coulouris G, Avagyan V, Ma N, Papadopoulos J, Bealer K, et al. BLAST+: architecture and applications. BMC Bioinformatics. 2009;10:421. 20003500 10.1186/1471-2105-10-421 PMC2803857

[104] Gabriel L, Hoff KJ, Brůna T, Borodovsky M, Stanke M. TSEBRA: transcript selector for BRAKER. BMC Bioinformatics. 2021;22:566. 34823473 10.1186/s12859-021-04482-0 PMC8620231

[105] Jones P, Binns D, Chang H-Y, Fraser M, Li W, McAnulla C, et al. InterProScan 5: genome-scale protein function classification. Bioinformatics. 2014;30:1236–40. 24451626 10.1093/bioinformatics/btu031 PMC3998142

[106] Andrews S. FastQC: A quality control tool for high throughput sequence data. 2023. Available from: https://www.bioinformatics.babraham.ac.uk/projects/fastqc/.

[107] Li H. A statistical framework for SNP calling, mutation discovery, association mapping and population genetical parameter estimation from sequencing data. Bioinformatics. 2011;27:2987–93. 21903627 10.1093/bioinformatics/btr509 PMC3198575

[108] Broad Institute. Picard toolkit. Broad Institute; 2019. Available from: https://broadinstitute.github.io/picard/.

[109] Poplin R, Ruano-Rubio V, DePristo MA, Fennell TJ, Carneiro MO, Van der Auwera GA, et al. Scaling accurate genetic variant discovery to tens of thousands of samples. bioRxiv. 2018. p. 201178.

[110] Danecek P, Auton A, Abecasis G, Albers CA, Banks E, DePristo MA, et al. The variant call format and VCFtools. Bioinformatics. 2011;27:2156–8. 21653522 10.1093/bioinformatics/btr330 PMC3137218

[111] Kofler R, Pandey RV, Schlötterer C. PoPoolation2: identifying differentiation between populations using sequencing of pooled DNA samples (Pool-Seq). Bioinformatics. 2011;27:3435–6. 22025480 10.1093/bioinformatics/btr589 PMC3232374

[112] Pedersen BS, Quinlan AR. Mosdepth: quick coverage calculation for genomes and exomes. Bioinformatics. 2018;34:867–8. 29096012 10.1093/bioinformatics/btx699 PMC6030888

[113] Weir BS, Cockerham CC. Estimating f-statistics for the analysis of population structure. Evolution. 1984;38:1358–70. 28563791 10.1111/j.1558-5646.1984.tb05657.x

[114] Xu D, Yang J, Wen H, Feng W, Zhang X, Hui X, et al. CentIER: accurate centromere identification for plant genomes. Plant Communications. 2024;5:101046. 39118326 10.1016/j.xplc.2024.101046 PMC11573919

[115] Kurtz S, Phillippy A, Delcher AL, Smoot M, Shumway M, Antonescu C, et al. Versatile and open software for comparing large genomes. Genome Biol. 2004;5:R12. 14759262 10.1186/gb-2004-5-2-r12 PMC395750

[116] Goel M, Sun H, Jiao W-B, Schneeberger K. SyRI: finding genomic rearrangements and local sequence differences from whole-genome assemblies. Genome Biol. 2019;20:277. 31842948 10.1186/s13059-019-1911-0 PMC6913012

[117] Goel M, Schneeberger K. plotsr: visualizing structural similarities and rearrangements between multiple genomes. Bioinformatics. 2022;38:2922–6. 35561173 10.1093/bioinformatics/btac196 PMC9113368

[118] Ellinghaus D, Kurtz S, Willhoeft U. LTRharvest, an efficient and flexible software for *de novo* detection of LTR retrotransposons. BMC Bioinformatics. 2008;9. 10.1186/1471-2105-9-18 PMC2253517 18194517

[119] Xu Z, Wang H. LTR_FINDER: an efficient tool for the prediction of full-length LTR retrotransposons. Nucleic Acids Res. 2007;35:W265–8. 17485477 10.1093/nar/gkm286 PMC1933203

[120] Ingvarsson PK. Multilocus patterns of nucleotide polymorphism and the demographic history of *Populus tremula*. Genetics. 2008;180:329–40. 18716330 10.1534/genetics.108.090431 PMC2535685

[121] Xie J, Li Y, Liu X, Zhao Y, Li B, Ingvarsson PK, et al. Evolutionary origins of pseudogenes and their association with regulatory sequences in plants. Plant Cell. 2019;31:563–78. 30760562 10.1105/tpc.18.00601 PMC6482637

[122] Emms DM, Kelly S. OrthoFinder: solving fundamental biases in whole genome comparisons dramatically improves orthogroup inference accuracy. Genome Biol. 2015;16:157. 26243257 10.1186/s13059-015-0721-2 PMC4531804

[123] Cingolani P, Platts A, Wang LL, Coon M, Nguyen T, Wang L, et al. A program for annotating and predicting the effects of single nucleotide polymorphisms, SnpEff. Fly. 2012;6:80–92. 22728672 10.4161/fly.19695 PMC3679285

[124] Yang Z, Nielsen R. Estimating synonymous and nonsynonymous substitution rates under realistic evolutionary models. Mol Biol Evol. 2000;17:32–43. 10666704 10.1093/oxfordjournals.molbev.a026236

[125] Wang D, Zhang Y, Zhang Z, Zhu J, Yu J. KaKs_Calculator 2.0: a toolkit incorporating gamma-series methods and sliding window strategies. Genomics Proteomics Bioinformatics. 2010;8:77–80. 20451164 10.1016/S1672-0229(10)60008-3 PMC5054116

[126] Zhang Z, Xiao J, Wu J, Zhang H, Liu G, Wang X, et al. ParaAT: a parallel tool for constructing multiple protein-coding DNA alignments. Biochem Biophys Res Commun. 2012;419:779–81. 22390928 10.1016/j.bbrc.2012.02.101

[127] Minh BQ, Schmidt HA, Chernomor O, Schrempf D, Woodhams MD, Von HA, et al. IQ-TREE 2: new models and efficient methods for phylogenetic inference in the genomic era. Mol Biol Evol. 2020;37:1530–4. 32011700 10.1093/molbev/msaa015 PMC7182206

[128] Kalyaanamoorthy S, Minh BQ, Wong TKF, von Haeseler A, Jermiin LS. Modelfinder: fast model selection for accurate phylogenetic estimates. Nat Methods. 2017;14:587–9. 28481363 10.1038/nmeth.4285 PMC5453245

[129] Paradis E, Claude J, Strimmer K. APE: analyses of phylogenetics and evolution in R language. Bioinformatics. 2004;20:289–90. 14734327 10.1093/bioinformatics/btg412

[130] R Core Team. R: A language and environment for statistical computing. Vienna, Austria: R Foundation for Statistical Computing; 2024. Available from: https://www.R-project.org/.

[131] Hyden B, Feng K, Yates TB, Jawdy S, Cereghino C, Smart LB, et al. *De Novo* assembly and annotation of 11 diverse shrub Willow (*Salix*) genomes reveals novel gene organization in sex-linked regions. Int J Mol Sci. 2023;24. 10.3390/ijms24032904 PMC9917877 36769224

[132] Wei S, Yang Y, Yin T. The chromosome-scale assembly of the willow genome provides insight into Salicaceae genome evolution. Hortic Res. 2020;7:45. 32257231 10.1038/s41438-020-0268-6 PMC7109076

[133] Pucholt P, Wright AE, Conze LL, Mank JE, Berlin S. Recent sex chromosome divergence despite ancient dioecy in the willow *Salix viminalis*. Mol Biol Evol. 2017;34:1991–2001. 28453634 10.1093/molbev/msx144 PMC5850815

[134] Zhang C, Rabiee M, Sayyari E, Mirarab S. ASTRAL-III: polynomial time species tree reconstruction from partially resolved gene trees. BMC Bioinformatics. 2018;19:153. 29745866 10.1186/s12859-018-2129-y PMC5998893

[135] Yang Z. PAML 4: phylogenetic analysis by maximum likelihood. Mol Biol Evol. 2007;24:1586–91. 17483113 10.1093/molbev/msm088

[136] Manchester SR, Dilcher DL, Tidwell WD. Interconnected reproductive and vegetative remains of *Populus* (Salicaceae) from the middle Eocene Green River formation, northeastern Utah. Am J Bot. 1986;73:156–60. 30139119 10.1002/j.1537-2197.1986.tb09691.x

[137] Boucher LD, Manchester SR, Judd WS. An extinct genus of Salicaceae based on twigs with attached flowers, fruits, and foliage from the Eocene Green River Formation of Utah and Colorado, USA. Am J Bot. 2003;90:1389–99. 21659238 10.3732/ajb.90.9.1389

[138] Jing Y, Lin R. The VQ motif-containing protein family of plant-specific transcriptional regulators. Plant Physiol. 2015;169:371–8. 26220951 10.1104/pp.15.00788 PMC4577417

[139] Zhang H, Zeng Y, Seo J, Kim Y-J, Kim ST, Kwon S-W. Global identification and characterization of C2 domain-containing proteins associated with abiotic stress response in rice (*Oryza sativa* L.). Int J Mol Sci. 2022;23:2221. 35216337 10.3390/ijms23042221 PMC8875736

[140] Yoo SY, Kim Y, Kim SY, Lee JS, Ahn JH. Control of flowering time and cold response by a NAC-domain protein in *Arabidopsis*. PLoS ONE. 2007;2:e642. 17653269 10.1371/journal.pone.0000642 PMC1920552

[141] Liu D, Gong Q, Ma Y, Li P, Li J, Yang S, et al. cpSecA, a thylakoid protein translocase subunit, is essential for photosynthetic development in *Arabidopsis*. J Exp Bot. 2010;61:1655–69. 20194926 10.1093/jxb/erq033

[142] Bruno L, Chiappetta A, Muzzalupo I, Gagliardi C, Iaria D, Bruno A, et al. Role of geranylgeranyl reductase gene in organ development and stress response in olive (*Olea europaea*) plants. Funct Plant Biol. 2009;36:370–81. 32688654 10.1071/FP08219

[143] Mateo-Bonmatí E, Esteve-Bruna D, Juan-Vicente L, Nadi R, Candela H, Lozano FM, et al. INCURVATA11 and CUPULIFORMIS2 are redundant genes that encode epigenetic machinery components in *Arabidopsis*. Plant Cell. 2018;30:1596–616. 29915151 10.1105/tpc.18.00300 PMC6096603

[144] El-Azaz J, Cánovas FM, Ávila C, la Torre FD. The arogenate dehydratase ADT2 is essential for seed development in *Arabidopsis*. Plant Cell Physiol. 2018;59:2409–20. 30289532 10.1093/pcp/pcy200

[145] Yang DH, Kwak KJ, Kim MK, Park SJ, Yang K-Y, Kang H. Expression of *Arabidopsis* glycine-rich RNA-binding protein AtGRP2 or AtGRP7 improves grain yield of rice (*Oryza sativa*) under drought stress conditions. Plant Sci. 2014;214:106–12. 24268168 10.1016/j.plantsci.2013.10.006

[146] Ahn H-K, Yoon J-T, Choi I, Kim S, Lee H-S, Pai H-S. Functional characterization of chaperonin containing T-complex polypeptide-1 and its conserved and novel substrates in *Arabidopsis*. J Exp Bot. 2019;70:2741–57. 30825377 10.1093/jxb/erz099 PMC6506772

[147] Bonaventure G, Salas JJ, Pollard MR, Ohlrogge JB. Disruption of the FATB gene in *Arabidopsis* demonstrates an essential role of saturated fatty acids in plant growth. Plant Cell. 2003;15:1020–33. 12671095 10.1105/tpc.008946 PMC152346

[148] Anders N, Wilkinson MD, Lovegrove A, Freeman J, Tryfona T, Pellny TK, et al. Glycosyl transferases in family 61 mediate arabinofuranosyl transfer onto xylan in grasses. Proc Natl Acad Sci U S A. 2012;109:989–93. 22215597 10.1073/pnas.1115858109 PMC3271882

[149] Wei K, Han P. Comparative functional genomics of the TPR gene family in *Arabidopsis*, rice and maize. Mol Breed. 2017;37:1–18.28127252

[150] Gong Z, Dong C-H, Lee H, Zhu J, Xiong L, Gong D, et al. A DEAD box RNA helicase is essential for mRNA export and important for development and stress responses in *Arabidopsis*. Plant Cell. 2005;17:256–67. 15598798 10.1105/tpc.104.027557 PMC544503

[151] Havecker ER, Gao X, Voytas DF. The diversity of LTR retrotransposons. Genome Biol. 2004;5:225. 15186483 10.1186/gb-2004-5-6-225 PMC463057

[152] Zeiner A, Colina FJ, Citterico M, Wrzaczek M. Cysteine-rich receptor-like protein kinases: their evolution, structure, and roles in stress response and development. J Exp Bot. 2023;74:4910–27. 37345909 10.1093/jxb/erad236

[153] Ali MA, Abbas A, Azeem F, Shahzadi M, Bohlmann H. The *Arabidopsis* GPI-anchored LTPg5 encoded by At3g22600 has a role in resistance against a diverse range of pathogens. Int J Mol Sci. 2020;21:1774. 32150834 10.3390/ijms21051774 PMC7084707

[154] Li C, Krishnan S, Zhang M, Hu D, Meng D, Riedelsberger J, et al. Alternative splicing underpins the ALMT9 transporter function for vacuolar Malic acid accumulation in apple. Adv Sci. 2024;11:e2310159. 10.1002/advs.202310159 PMC11165477 38514904

[155] Soares A, Ribeiro Carlton SM, Simões I. Atypical and nucellin-like aspartic proteases: emerging players in plant developmental processes and stress responses. J Exp Bot. 2019;70:2059–76. 30715463 10.1093/jxb/erz034

[156] Binmöller L, Volkert C, Kiefer C, Zühl L, Slawinska MW, Loreth A, et al. Differential expression and evolutionary diversification of RNA helicases in *Boechera* sexual and apomictic reproduction. J Exp Bot. 2024;75:2451–69. 38263359 10.1093/jxb/erae026

[157] Tang H, Krishnakumar V, Zeng X, Xu Z, Taranto A, Lomas JS, et al. JCVI: A versatile toolkit for comparative genomics analysis. Imeta. 2024;3:e211. 39135687 10.1002/imt2.211 PMC11316928

[158] Wang Y, Tang H, Debarry JD, Tan X, Li J, Wang X, et al. MCScanX: a toolkit for detection and evolutionary analysis of gene synteny and collinearity. Nucleic Acids Res. 2012;40:e49. 22217600 10.1093/nar/gkr1293 PMC3326336

[159] Edgar RC. Muscle: a multiple sequence alignment method with reduced time and space complexity. BMC Bioinformatics. 2004;5:113. 15318951 10.1186/1471-2105-5-113 PMC517706

[160] Mao X, Rafati N, Tellgren-Roth C, Ingvarsson P, Karrenberg S. Data for “Sex chromosome evolution mediated by a large inversion and a possible switch of the sex determination gene”. Zenodo; 2025. Available from: 10.5281/zenodo.18851941. 10.1186/s13059-026-04038-6 PMC13064059 41857659

[161] Mao X, Rafati N, Tellgren-Roth C, Ingvarsson P, Karrenberg S. Code for “Sex chromosome evolution mediated by a large inversion and a possible switch of the sex determination gene” (v1.0.0). GitHub; 2025. Available from: https://github.com/miaoxm2/Salix-SexChr.Genome. 10.1186/s13059-026-04038-6 PMC13064059 41857659

[162] Mao X, Rafati N, Tellgren-Roth C, Ingvarsson P, Karrenberg S. Code for “Sex chromosome evolution mediated by a large inversion and a possible switch of the sex determination gene” (v1.0.0). Zenodo; 2025. Available from: 10.5281/ZENODO.17276729. 10.1186/s13059-026-04038-6 PMC13064059 41857659

