# Supplementary material for: Sex chromosome evolution mediated by a large inversion and a possible switch of the sex determination gene
Source: Genome Biol. 2026 Mar 19;27:120. doi: 10.1186/s13059-026-04038-6 (PMC13064059; doi:10.1186/s13059-026-04038-6)
Supplement: Supplementary file 1 — Additional file 1: Figures S1-9. [file 13059_2026_4038_MOESM1_ESM.pdf]

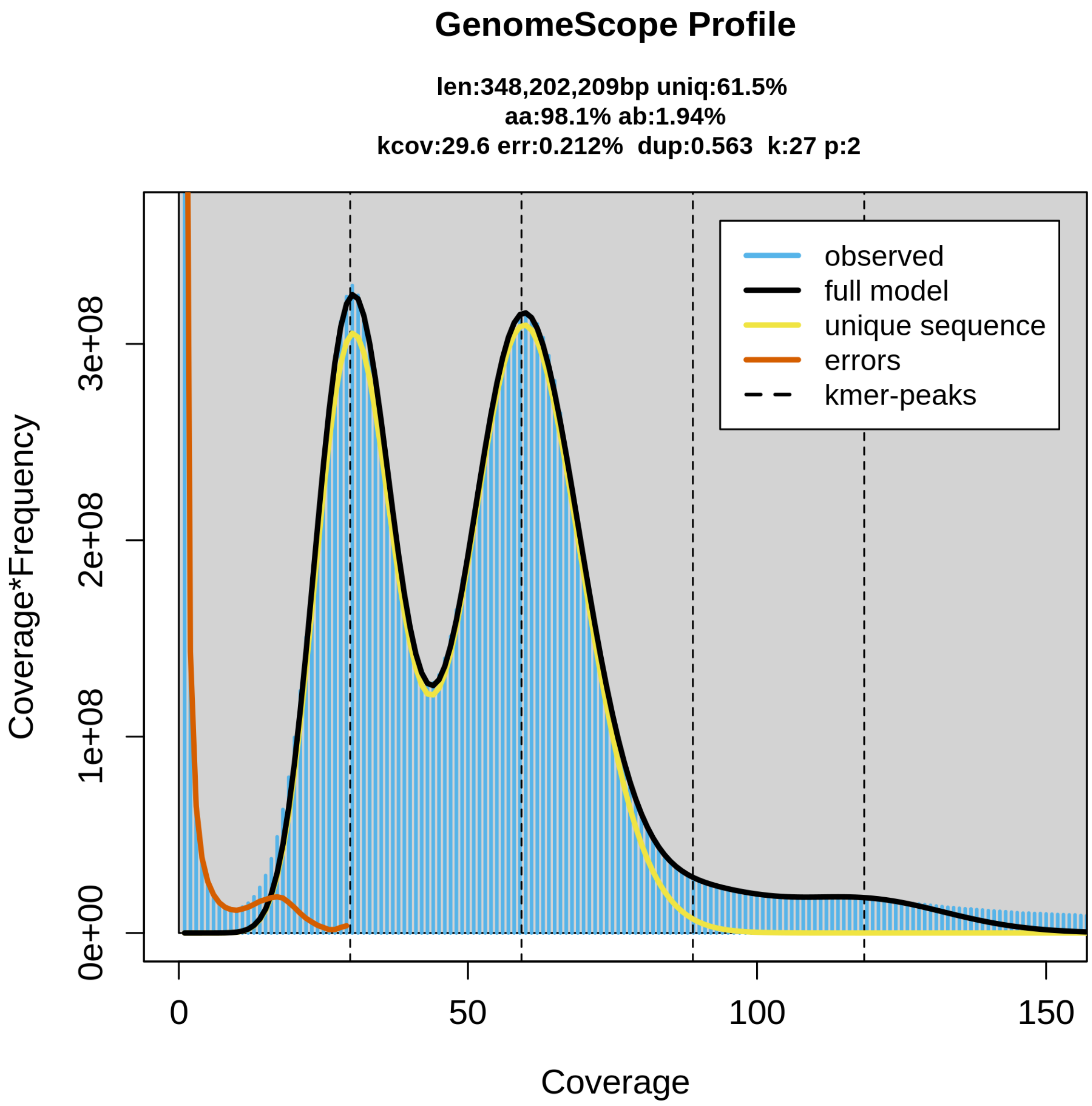

Supplementary Figure S1. The 27-mer distribution of the PacBio HiFi reads of *Salix herbacea*.

a. haplotype 1 of *Salix herbacea*

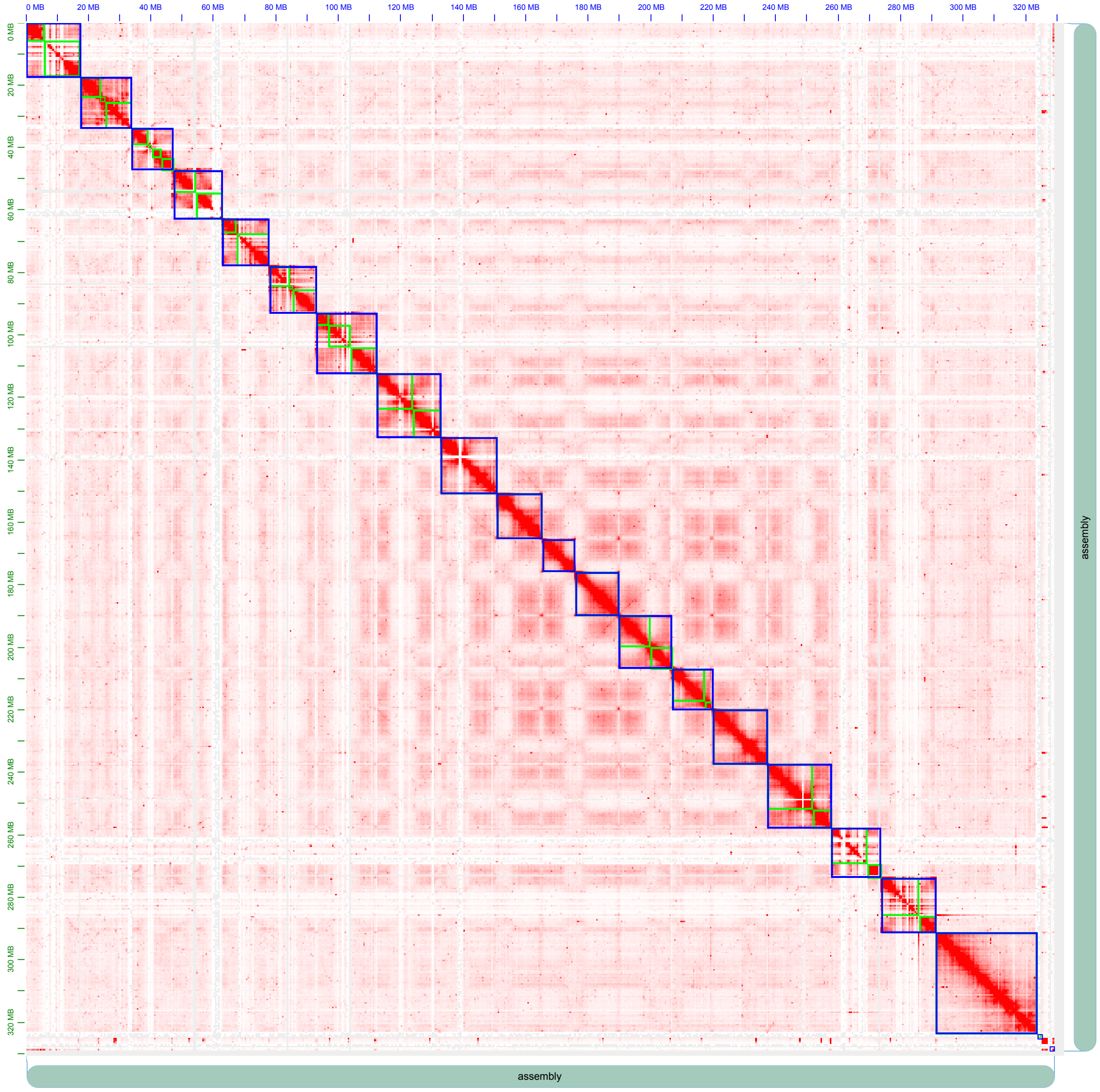

b. haplotype 2 of *Salix herbacea*

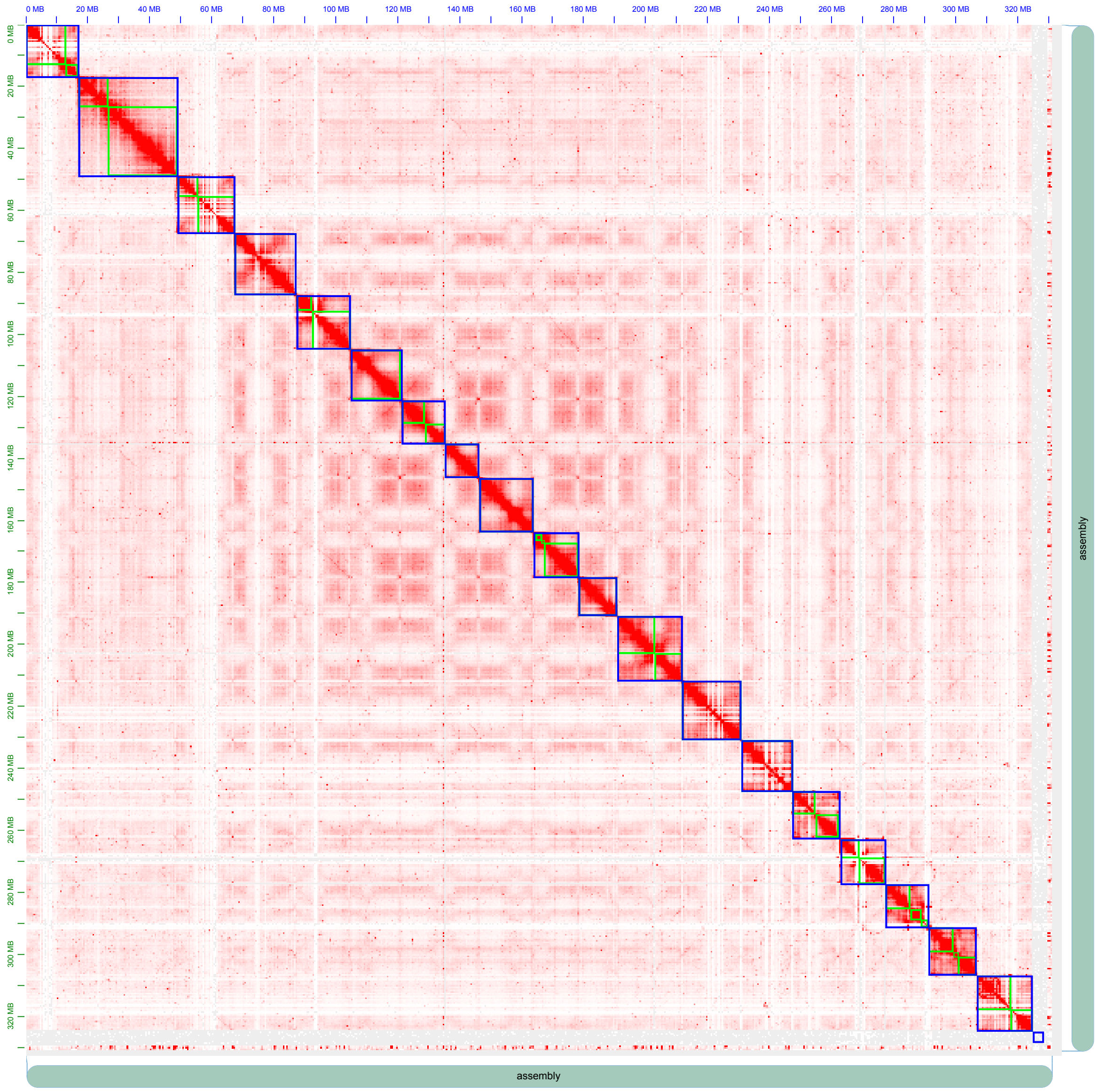

Figure S2. Genome-wide analysis of chromatin interactions at 1-Mb resolution in two haplotypes (a,b) of *Salix herbacea*.

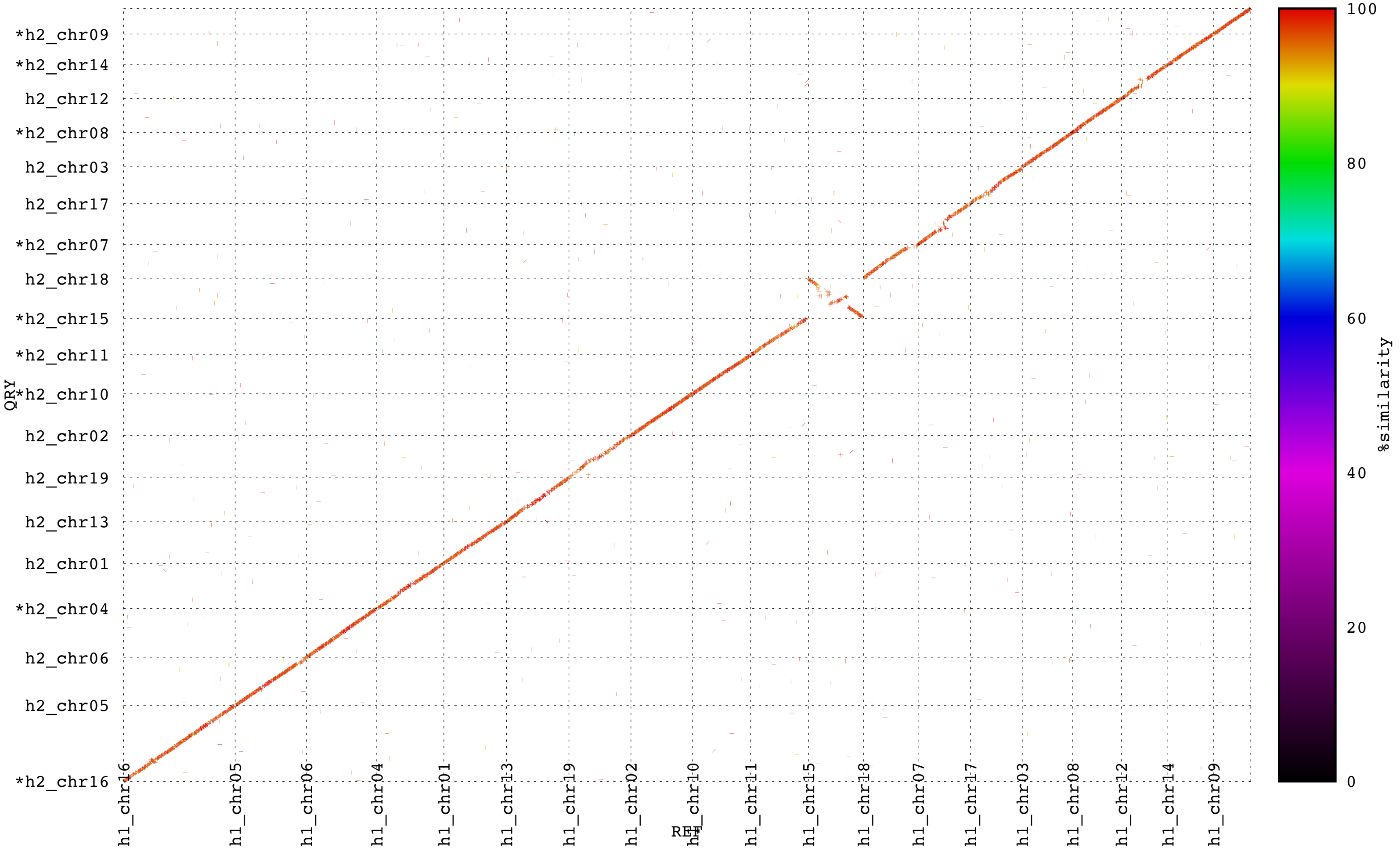

Figure S3. Alignment of the two haplotypes in a PacBio HiFi genome assembly of *Salix herbacea***.**

a. Read depth proportion of females in 10 female (F) and 10 male (M) individuals for haplotype 1

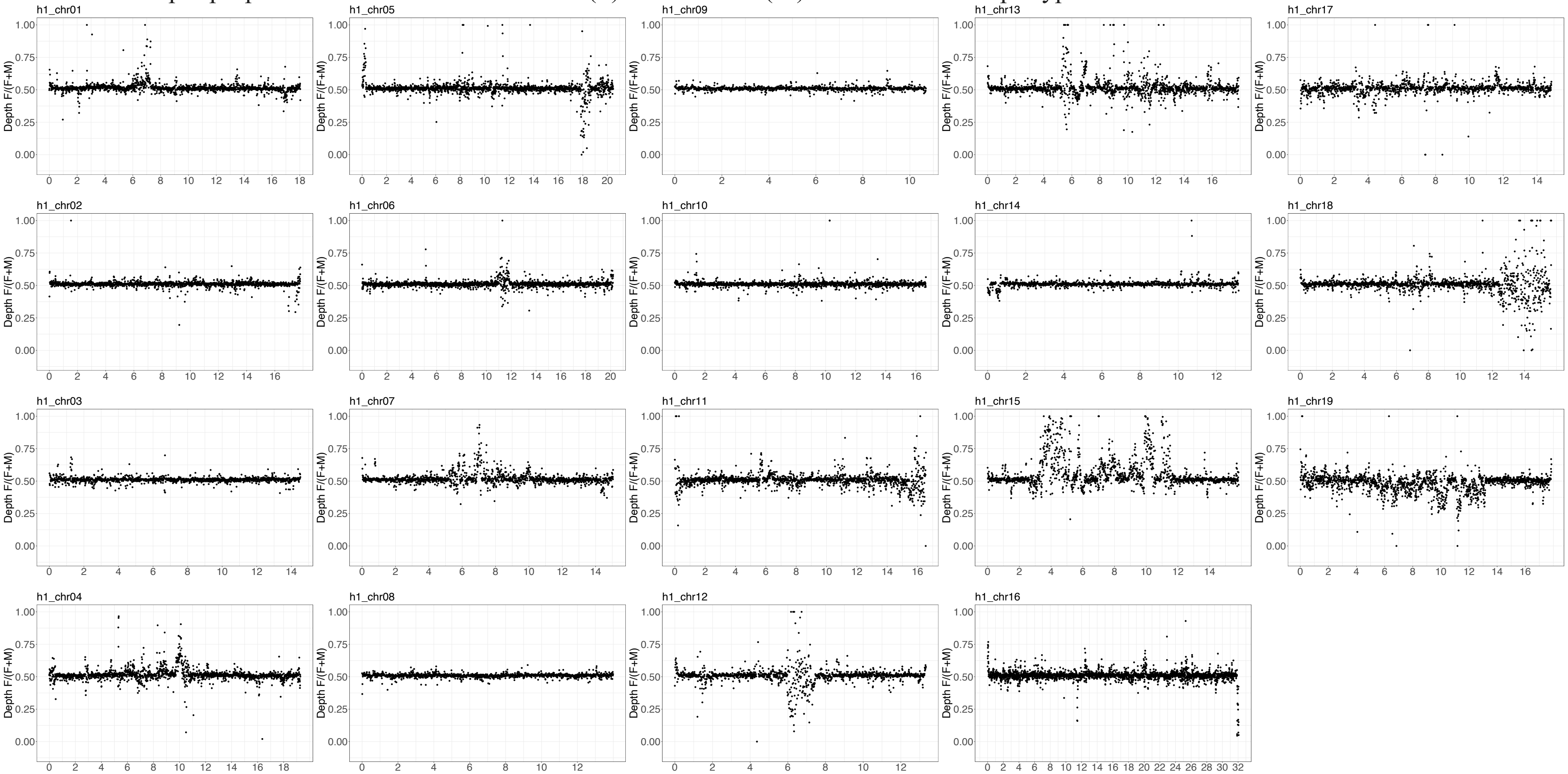

b. Read depth proportion of females in in pooled sequences of 50 females (F) and 50 males (M) for haplotype 1.

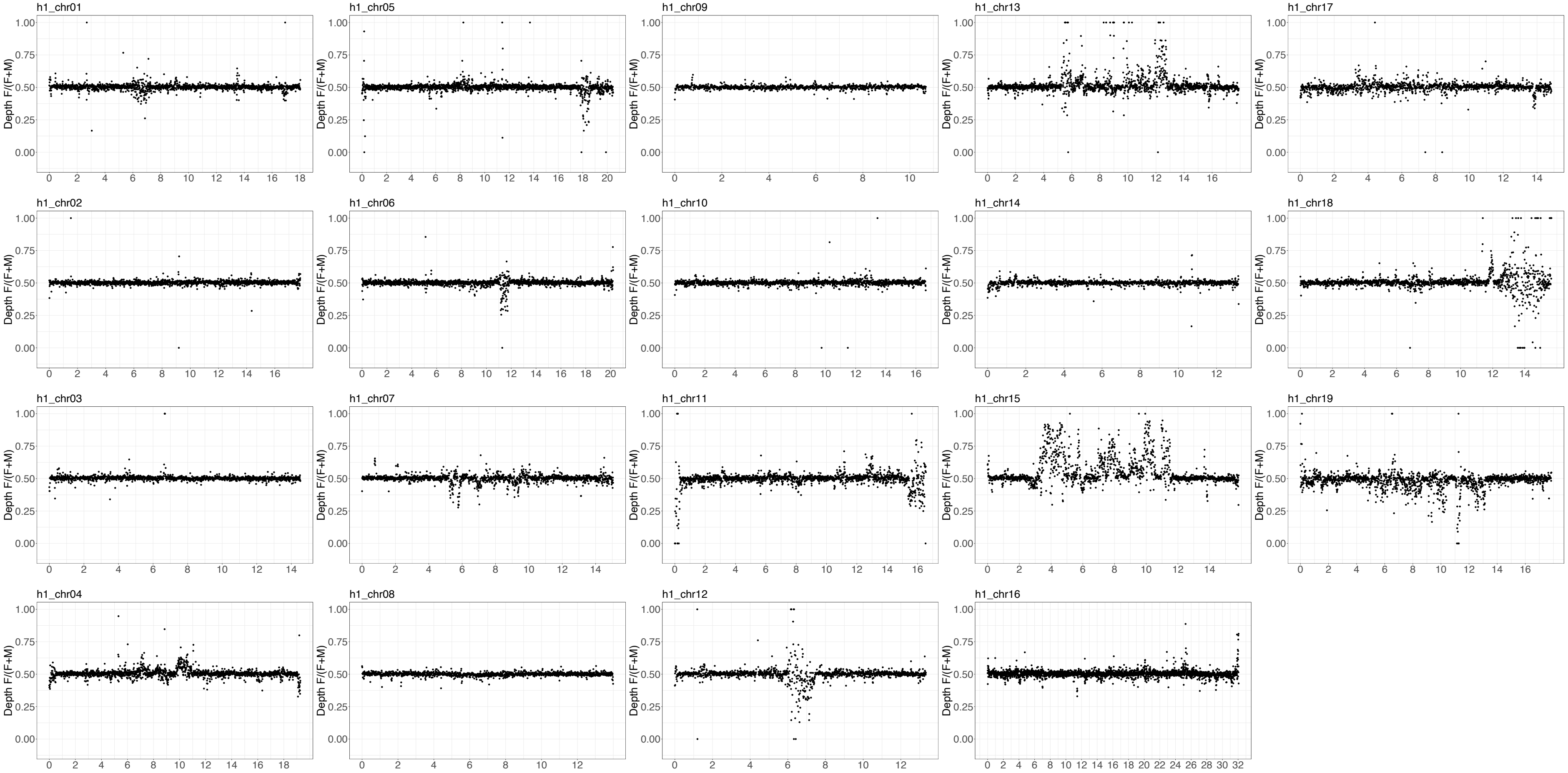

Figure S4. Read depth proportion of females in individual sequences of 10 females and 10 males **(a)** and pooled sequences of 50 females and 50 males **(b)** of *Salix herbacea*, calculated as F/(F+M) in 10 Kb windows mapped to **haplotype 1**.

a. Read depth proportion of females in 10 female (F) and 10 male (M) individuals for haplotype 2

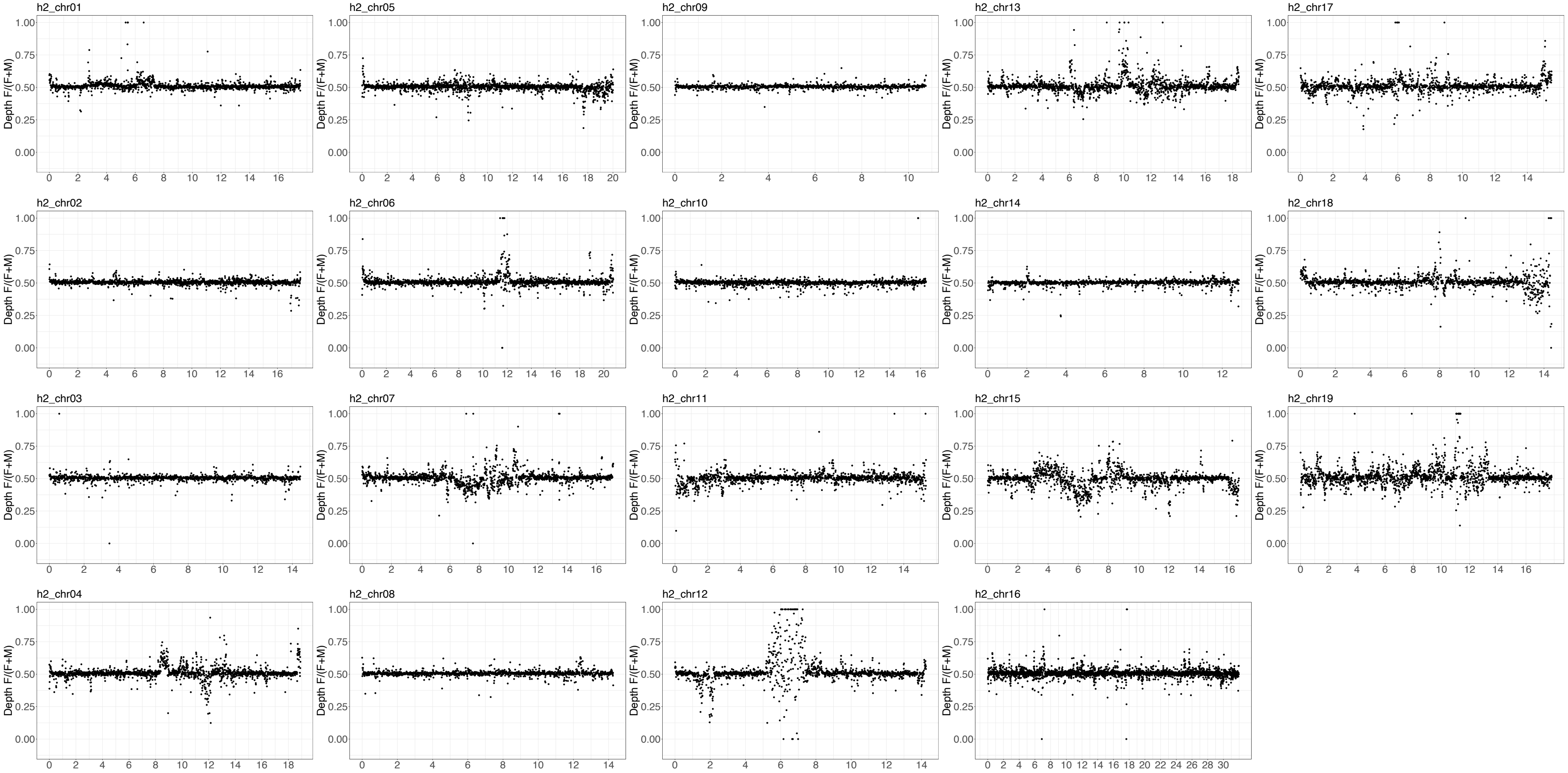

b. Read depth proportion of females in in pooled sequences of 50 females (F) and 50 males (M) for haplotype 2.

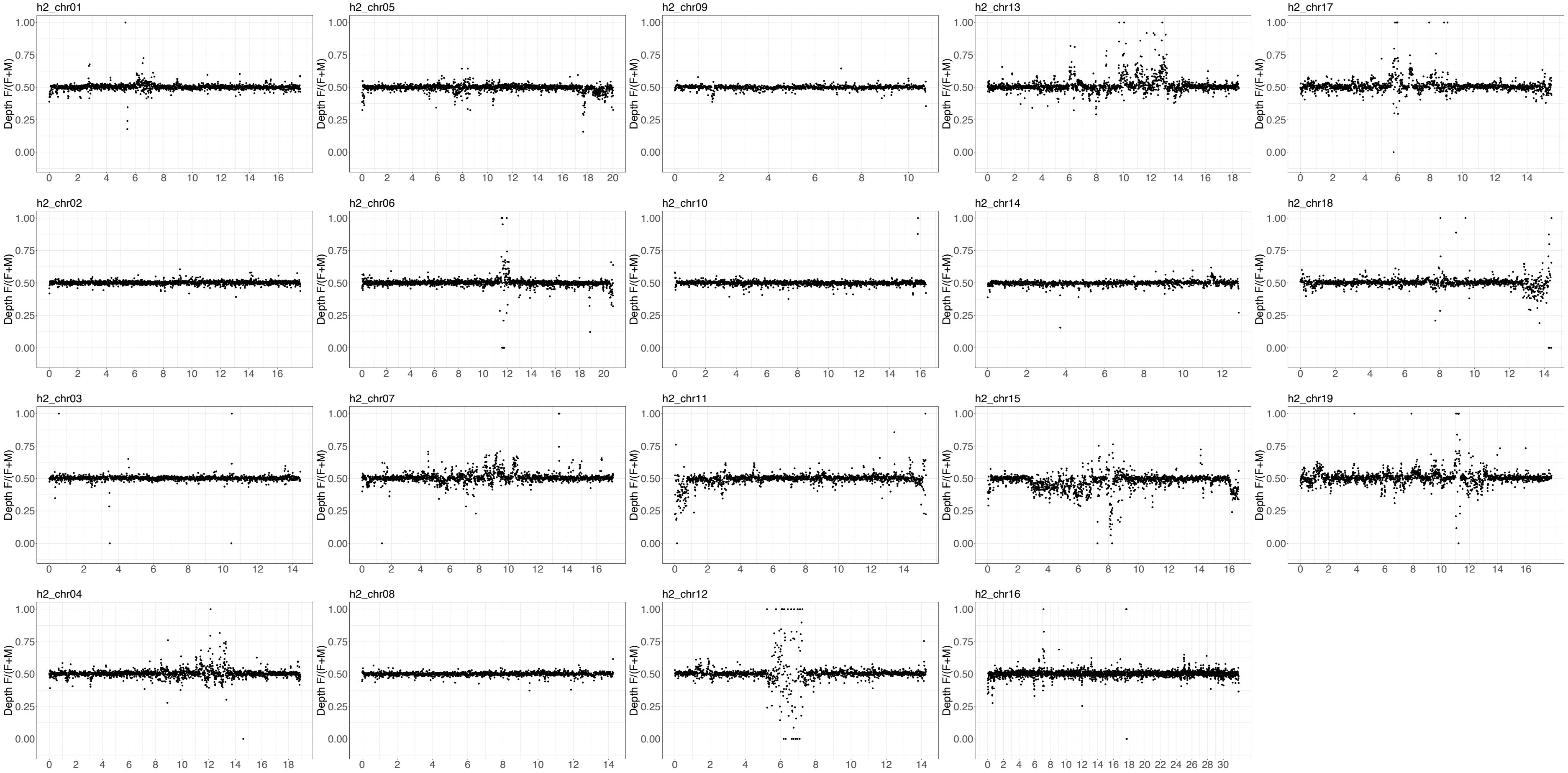

Figure S5. Read depth proportion of females in individual sequences of 10 females and 10 males **(a)** and pooled sequences of 50 females and 50 melas **(b)** of *Salix herbacea*, calculated as F/(F+M) in 10 Kb windows mapped to **haplotype 2**.

a. Genetic differentiation (F_{ST}) between 10 female (F) and 10 male (M) individuals mapped to haplotype 1

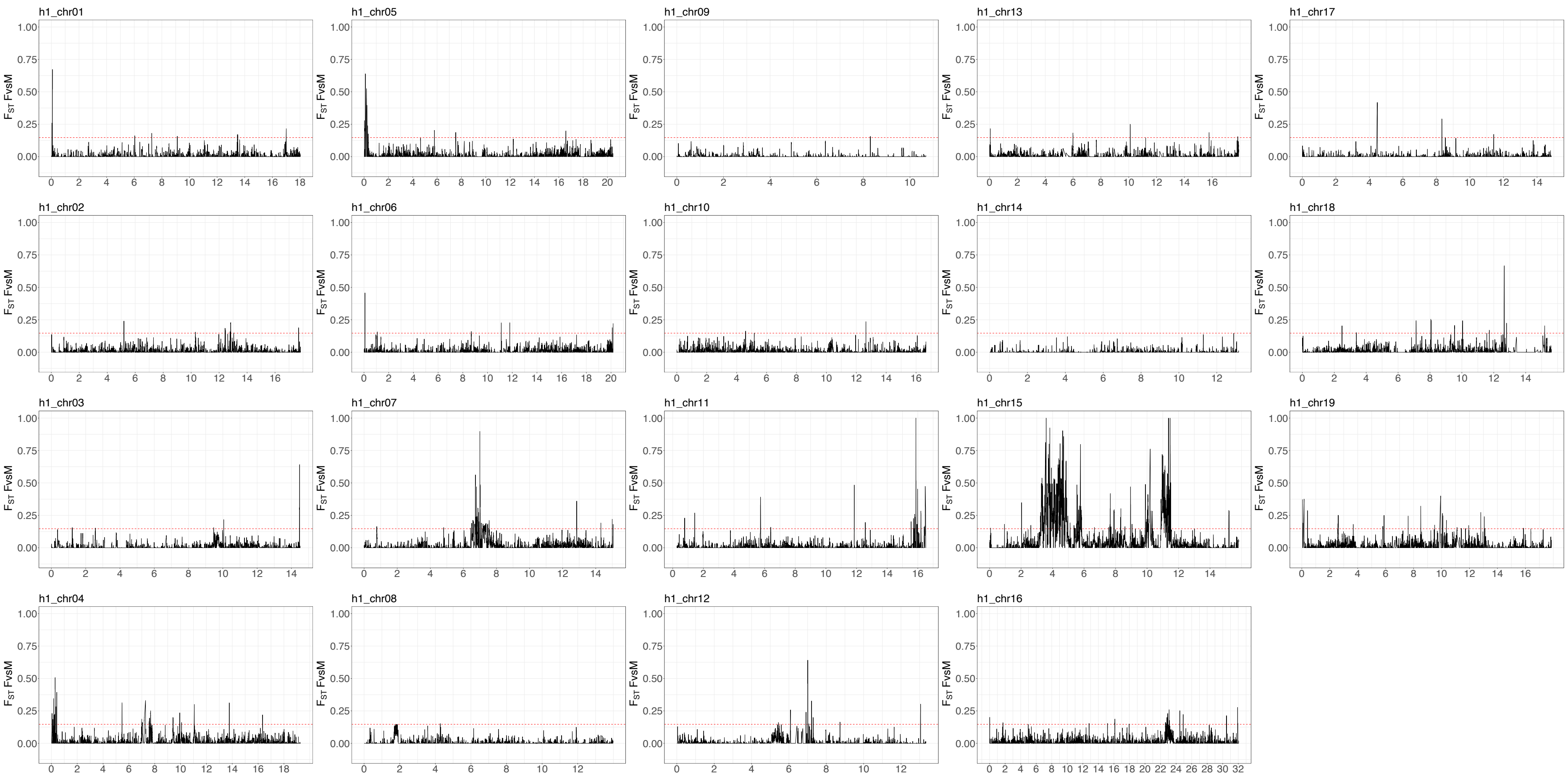

b. Genetic differentiation (F_{ST}) between pools of 50 females and 50 males mapped to haplotype 1

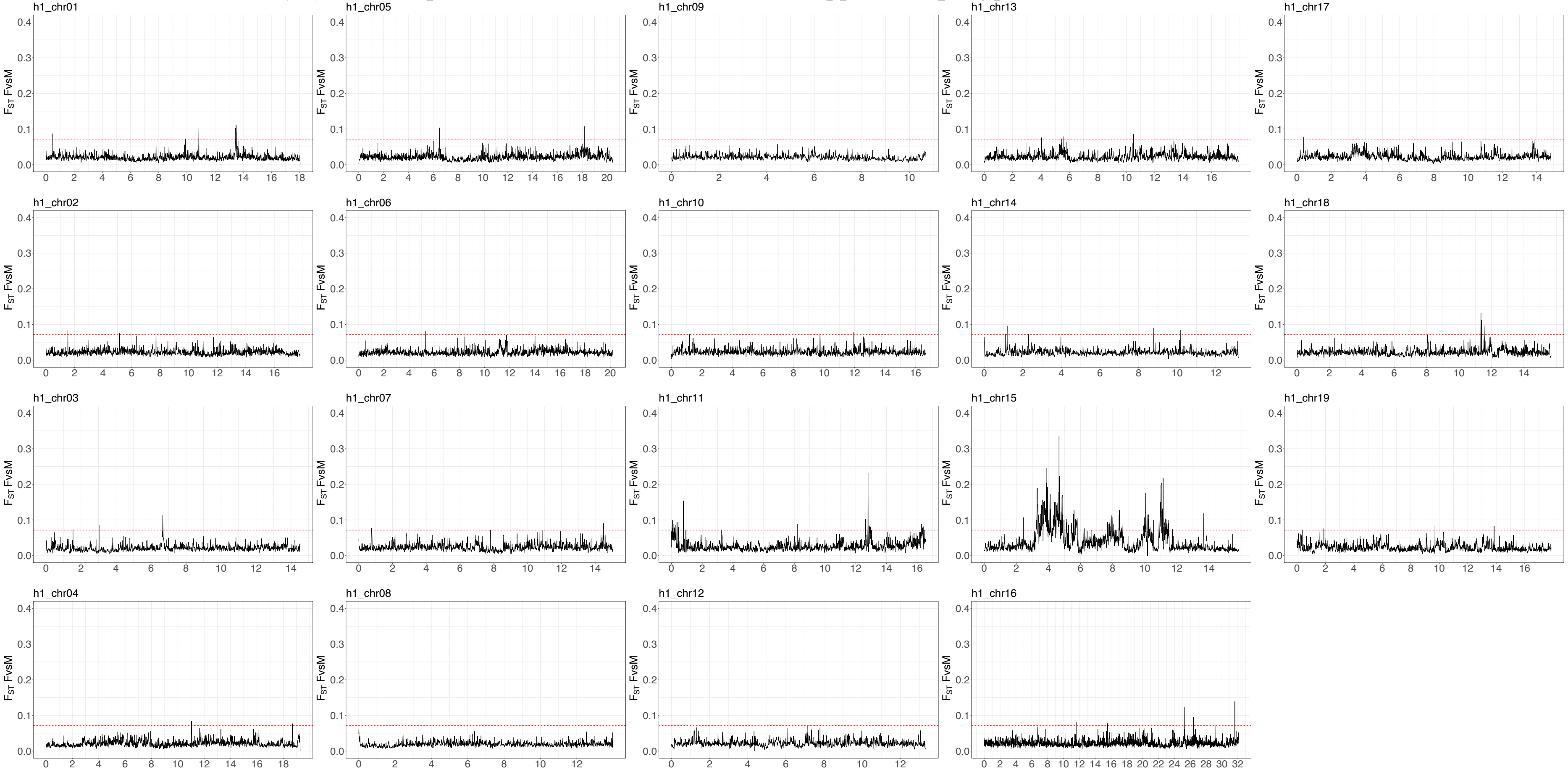

Figure S6. Between-sex genetic differentiation (F_{ST}) in *Salix herbacea* in 10 Kb windows mapped to **haplotype 1**: **(a)** F_{ST} between individual sequences of 10 females and 10 males and **(b)** F_{ST} between pools of 50 females and 50 males. The genome-wide 99th percentile indicated as a red dashed line

a. Genetic differentiation (F_{ST}) between 10 female (F) and 10 male (M) individuals mapped to haplotype 2

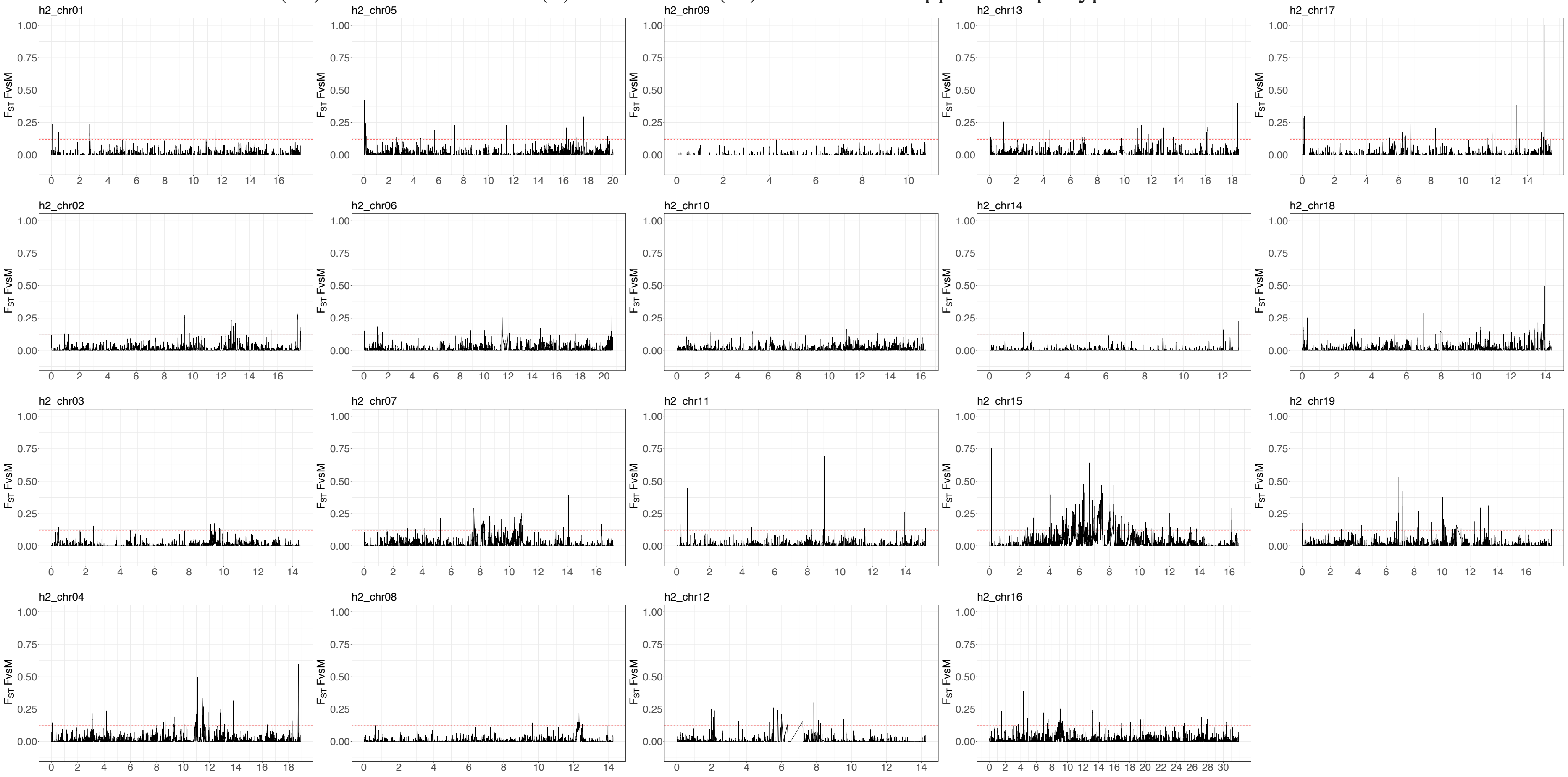

b. Genetic differentiation (F_{ST}) between pools of 50 females and 50 males mapped to haplotype 2

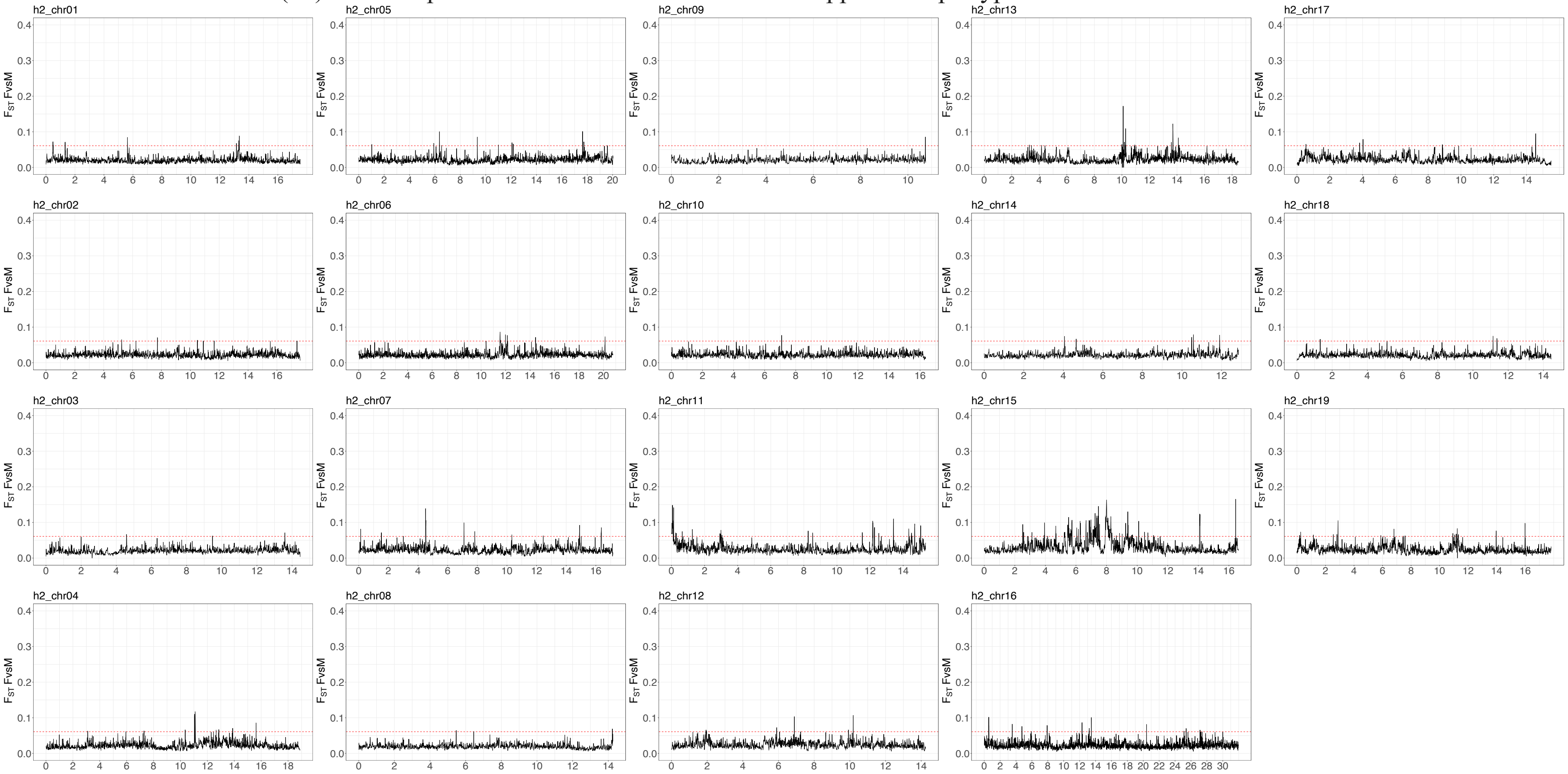

Figure S7. Between-sex genetic differentiation (F_{ST}) in *Salix herbacea* in 10 Kb windows mapped to **haplotype 2**: **(a)** F_{ST} between individual sequences of 10 females and 10 males and **(b)** F_{ST} between pools of 50 females and 50 males. The genome-wide 99th percentile indicated as a red dashed line.

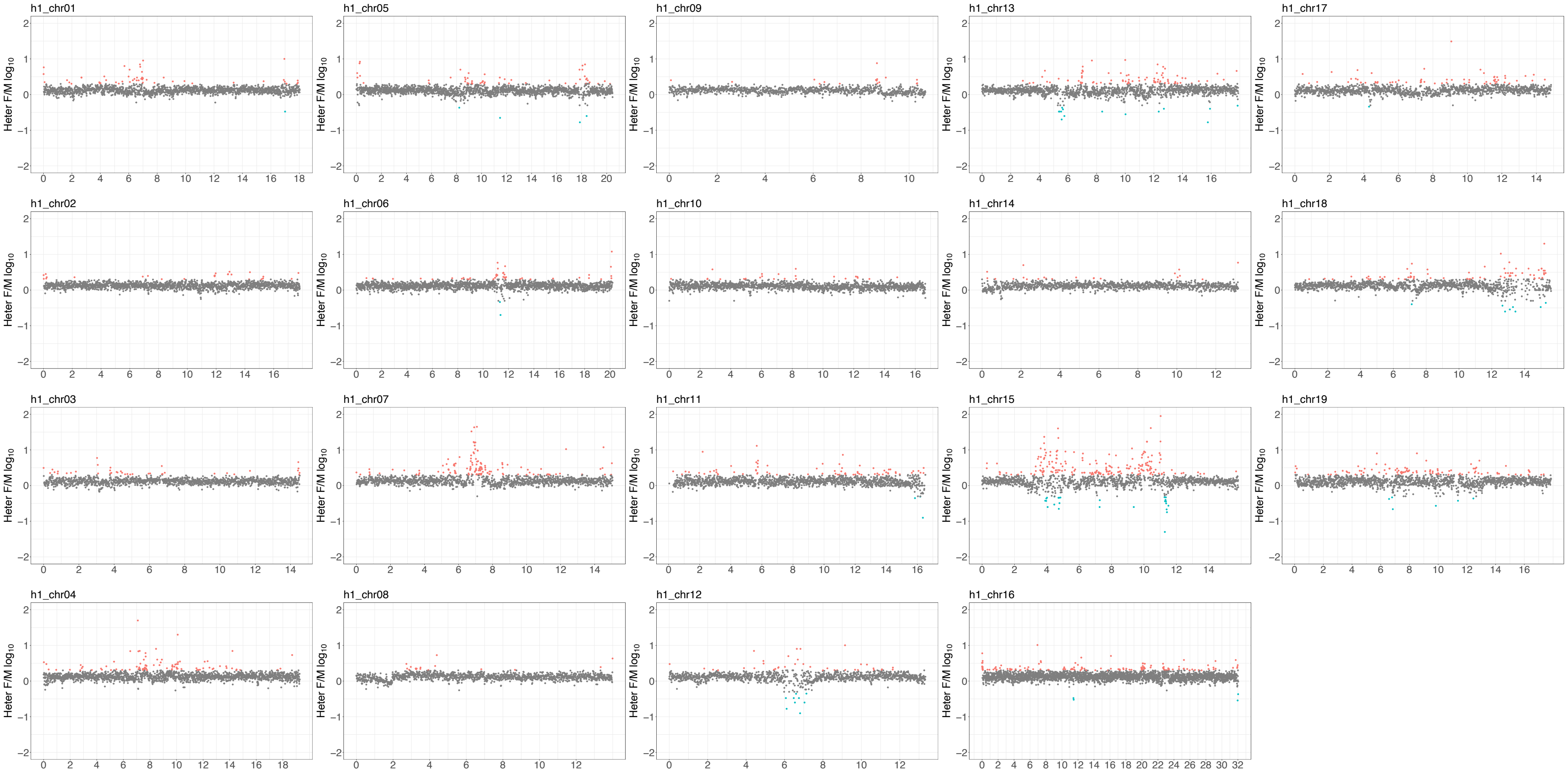

Figure S8. Ratio of the number of heterozygous sites between 10 females (F) and 10 males (M) in 10 Kb windows (F/ M), mapped to **haplotype 1**; a twofold excess of female or male heterozygous sites is indicated in red and blue colour, respectively.

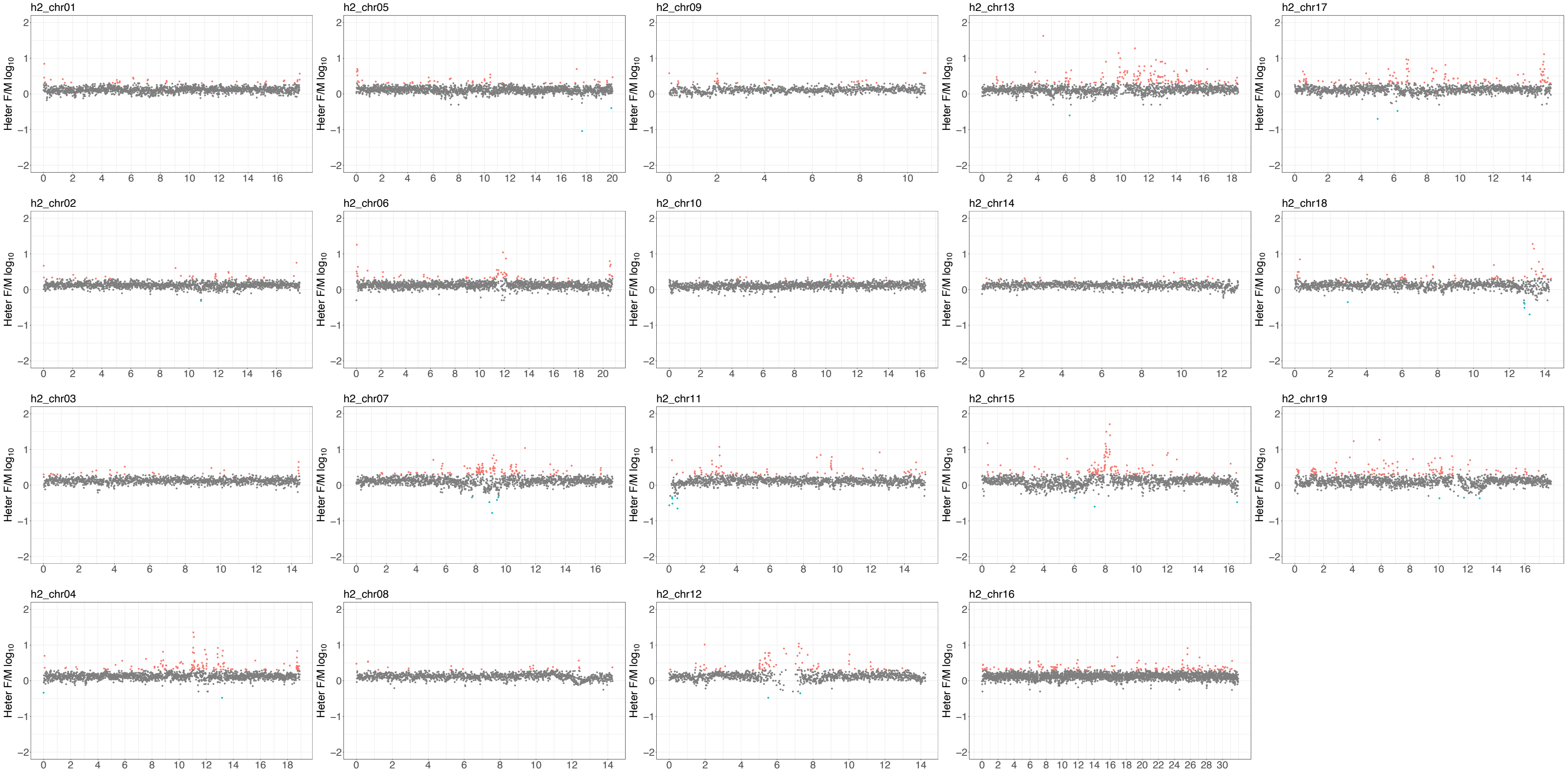

Figure S9. Ratio of the number of heterozygous sites between 10 females (F) and 10 males (M) of *Salix herbacea* in 10 Kb windows (F/ M), mapped to **haplotype 2**; a twofold excess of female or male heterozygous sites is indicated in red and blue colour, respectively.
