# Supplementary material for: Sex chromosome evolution mediated by a large inversion and a possible switch of the sex determination gene
Source: Genome Biol. 2026 Mar 19;27:120. doi: 10.1186/s13059-026-04038-6 (PMC13064059; doi:10.1186/s13059-026-04038-6)
Supplement: Supplementary file 2 — Additional file 2: Figures S10-16. [file 13059_2026_4038_MOESM2_ESM.pdf]

a. 3’ breakpoint

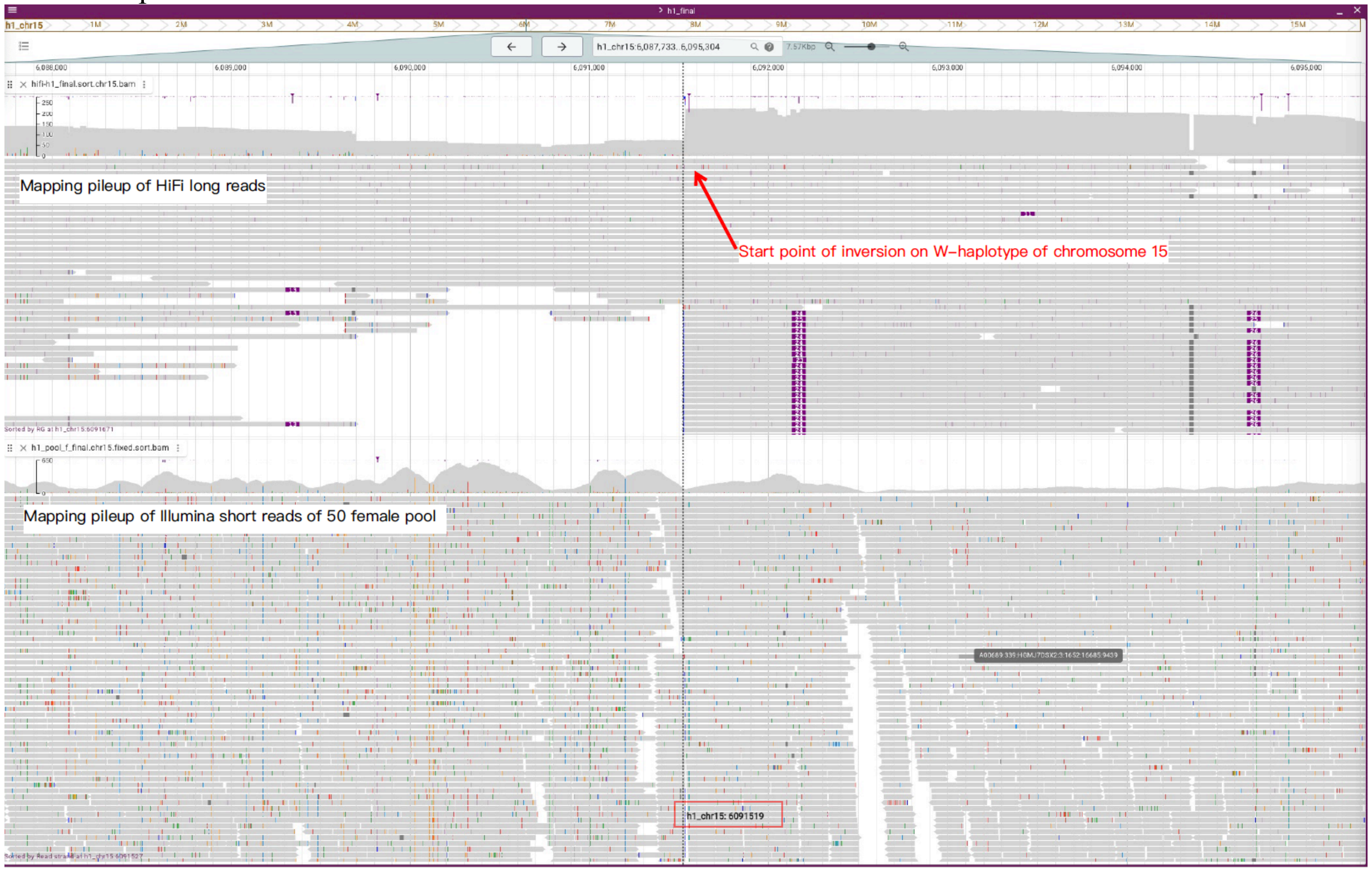

b. 5’ breakpoint

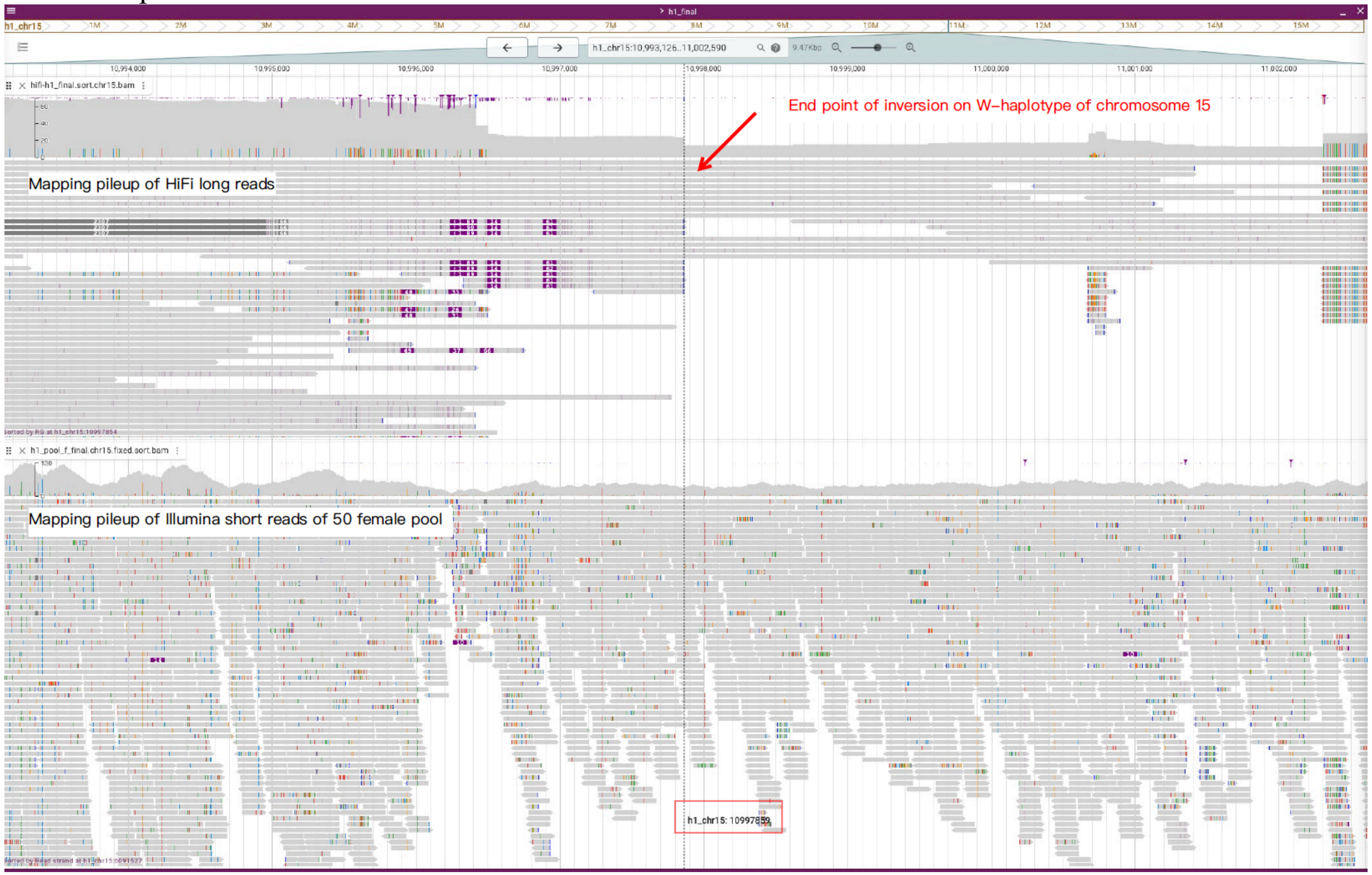

Figure S10. Mapping HiFi long reads and Illumina short reads to the 3' breakpoint (a) and the 5´breakpoint (b) of an inversion on haplotype 1 of chromosome 15 of *Salix herbacea*.

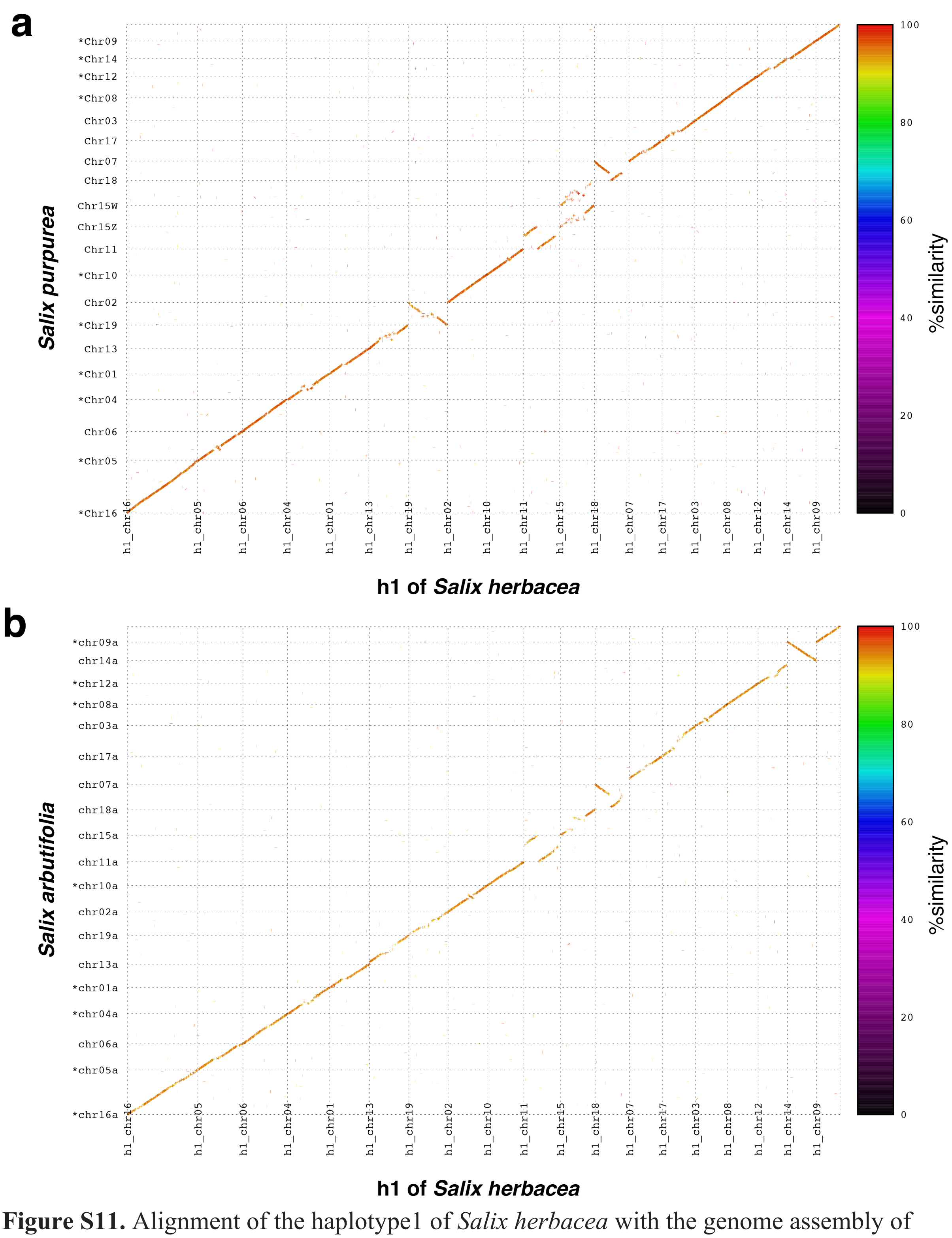

Figure S11. Alignment of the haplotype1 of *Salix herbacea* with the genome assembly of *Salix purpurea* (**a**) and *Salix arbutifolia* (**b**).

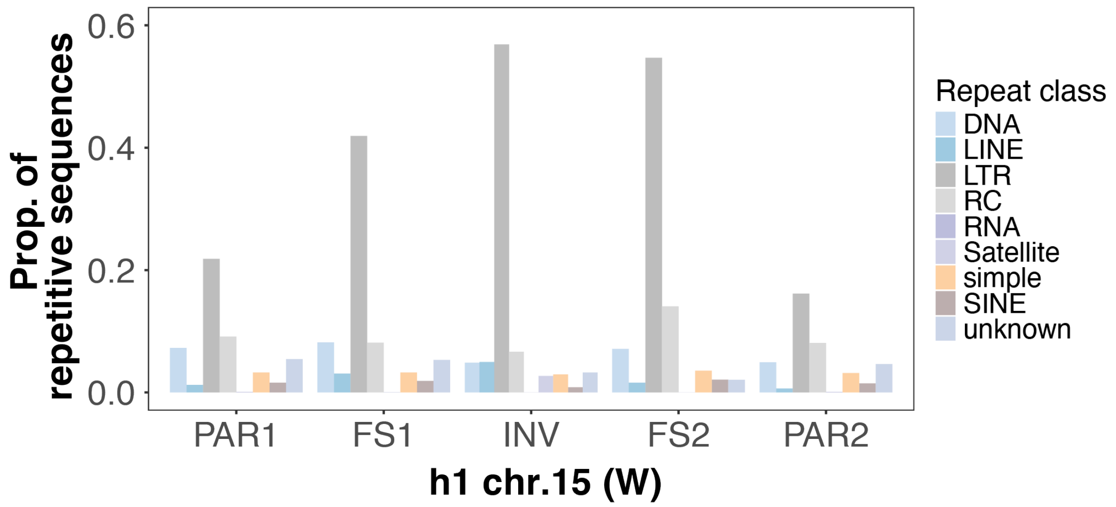

Figure S12. Proportion of repetitive sequences in five regions of the W-haploype of *Salix herbacea* (two distal pseudoautosomal regions : PAR1, PAR2; two-female specific regions: FS1, FS2; and a central inversion region: INV) for the*Salix herbacea* genome assembly.

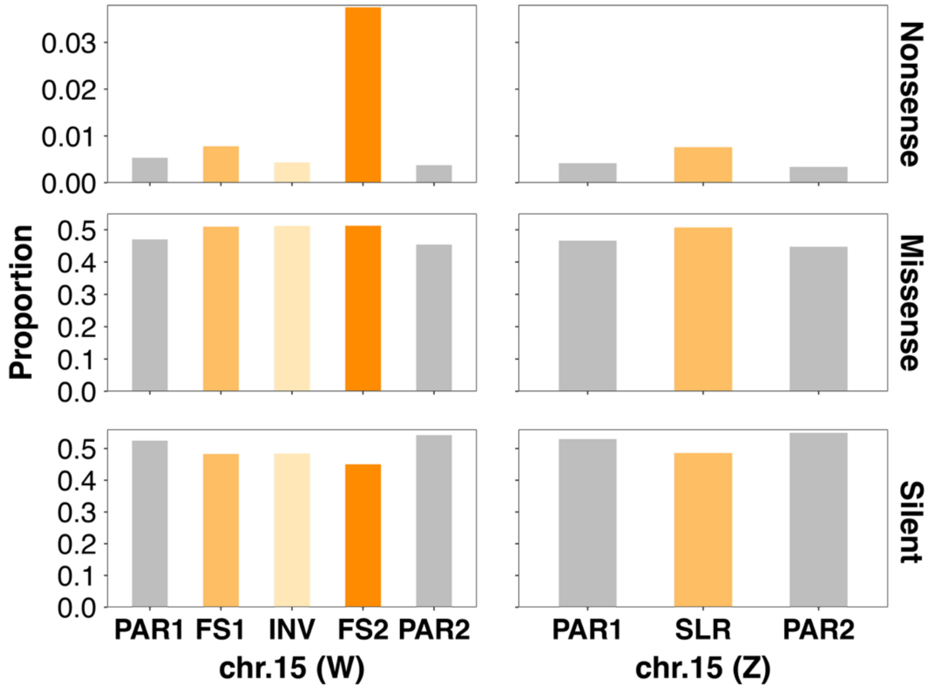

Figure S13. Proportion of polymorphisms across the study population with predicted silenet, missense and nonsense effects on gene function, across five regions (two distal pseudoautosomal regions : PAR1, PAR2; two-female specific regions: FS1, FS2; and a central inversion region: INV) of W-haplotye and two regions of Z-haplotype of *Salix herbacea*.

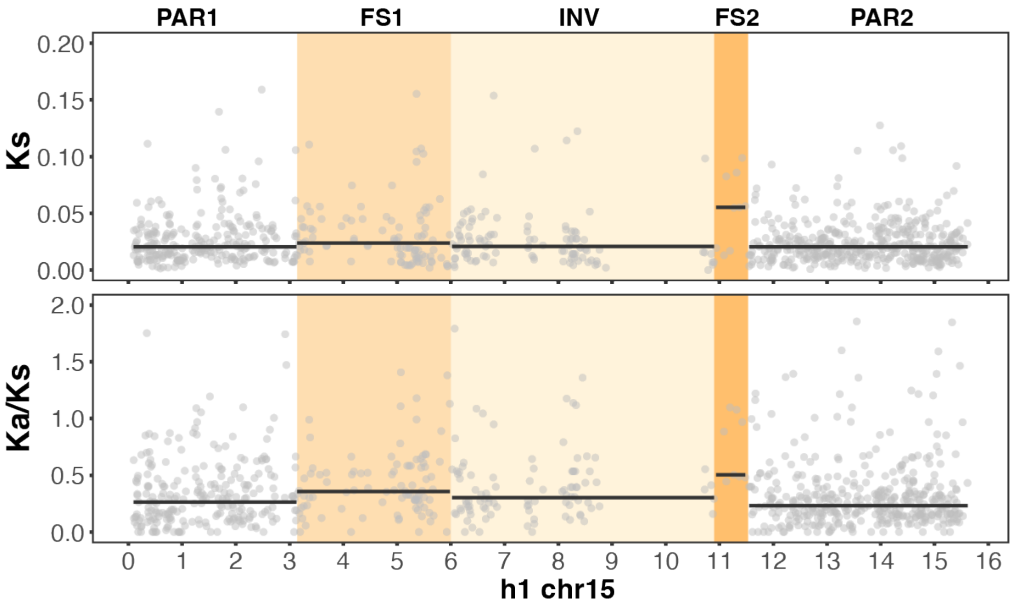

Figure S14. Synonymous site divergence rates (K_s) and the ratio between non-synonymous and synonymous divergence (K_a/K_s) of Z-W homologous genes in *Salix herbacea* with median indicated in each region. K_s values are shown within the range of 0–0.2 for clarity, K_a/K_s values are shown within the range of 0-2. Additional 5 genes with $K_s > 0.2$ and 54 genes with $K_a/K_s > 2$ are not shown.

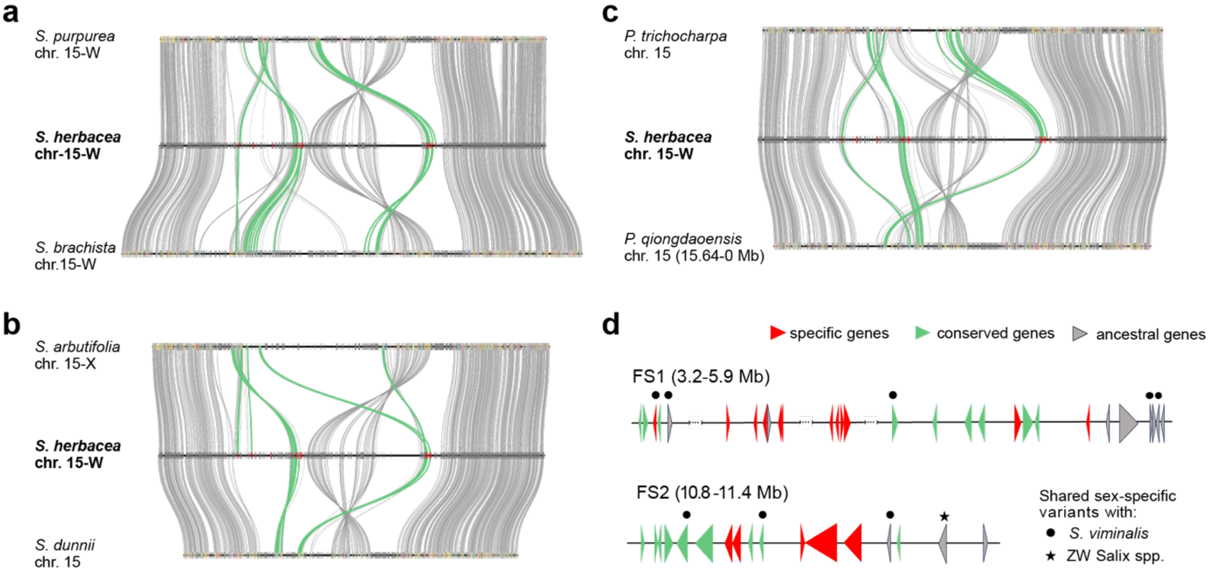

Figure S15. Gene synteny on chromosome 15 within the Salicaceae family. a. Synteny of *Salix herbacea* with other *Salix* species with 15ZW sex determination (*S. purpurea*, *S. brachista*), **b.** with *Salix* species with 15XY and 7XY sex-determining systems (*S. arbutifolia*, *S. dunnii*), **c.** with *Populus* species with 19XY and 19ZW sex-determining systems (*P. trichocarpa*, *P. qiongdaoensis*). Red ticks indicate genes with female-specific polymorphisms in *S. herbacea*, homologous genes among which are highlighted by green lines. **d.** Schematic representation of genes with female-specific polymorphisms in FS1 and FS2 of chromosome 15 of *S. herbacea*. Red triangles S. *herbacea* specific genes (present on chr. 15 only in *S. herbacea*), while green triangles represent their origin on chromosome 15 as conserved (detected on chr. 15 in each of three clades: ZW *Salix* species (*S. purpurea*, *S. suchowensis*, a), XY *Salix* species (*S. arbutifolia*, *S. dunnii*, b) and *Populus* species (*P. qiongdaoensis* and *P. trichocarpa*, c), and grey triangles indicate ancestral genes (detected on chr. 15 in at least one of these six species). Genes that have shared sex-specific variation with *S. viminalis* or with multiple species with a ZW sex-determination system are marked by circles or stars, respectively.

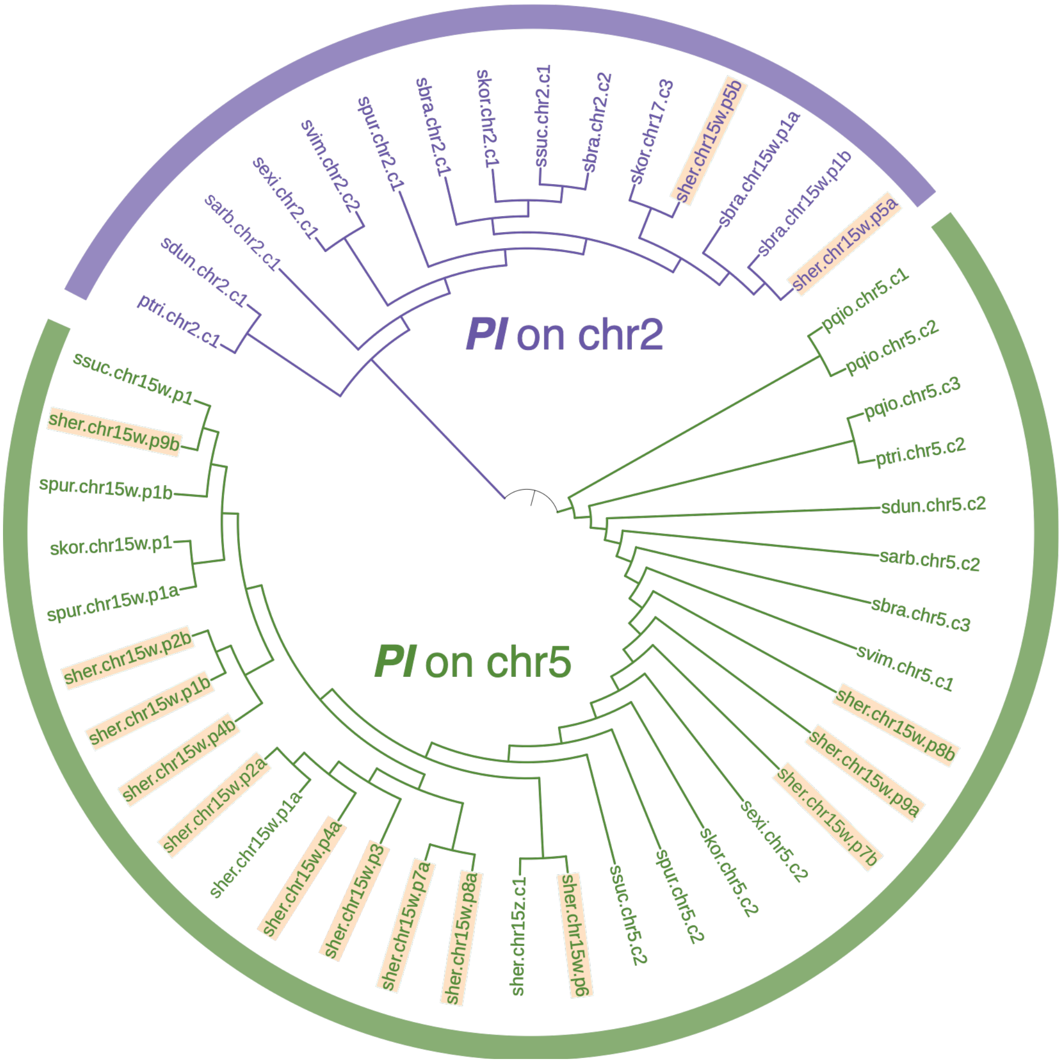

Figure S16. Phylogenetic tree of intact and partial PI genes in Salicaceae based on first exon sequences, with genes from chromosomes 2 and 5 shown in purple and green, respectively. Partial copies from *S. herbacea* are highlighted with a light yellow background.
