# Supplementary material for: Sex chromosome evolution mediated by a large inversion and a possible switch of the sex determination gene
Source: Genome Biol. 2026 Mar 19;27:120. doi: 10.1186/s13059-026-04038-6 (PMC13064059; doi:10.1186/s13059-026-04038-6)
Supplement: Supplementary file 3 — Additional file 3: Tables S1-9. [file 13059_2026_4038_MOESM3_ESM.pdf]

Supplementary Information

Table S1. Summary of raw reads generated for *Salix herbacea*.

Sample	before filtering			after filtering			duplication rate %	Q30 bases %	read coverage on haplotype 1
	Number of reads (x10^6)	Data size (Gb)	Average length (bp)	Number of reads (x10^6)	Data size (Gb)	Average length (bp)			
HiFi	NA	NA	NA	1.46	21.89	14944	NA	NA	67
HiC	308.69	46.3	150	306.76	45.82	149	6.33	96.53	140
Transcriptome	207.48	31.1	150	201.05	28.76	142	NA	NA	88
Female pool	294.42	44.46	151	292.36	43.61	149	1.68	93.05	133
Male pool	237.62	35.88	151	234.56	34.99	149	1.62	92.66	107
ind.f05	294.42	44.46	151	292.36	43.61	149	1.68	92.39	133
ind.f16	90.9	13.73	151	89.43	13.3	149	1.4	92.41	41
ind.f40	80.58	12.17	151	79.51	11.82	149	1.47	92.91	36
ind.f06	97.93	14.79	151	96.73	14.41	148	21.69	93.32	44
ind.f08	152.26	22.99	151	150.58	22.47	149	24.48	93.64	69
ind.f09	120.39	18.18	151	119.09	17.75	149	21.31	92.78	54
ind.f11	116.79	17.63	151	115.45	17.17	148	23.76	93.22	52
ind.f22	114.89	17.35	151	113.29	16.91	149	21.25	93.08	52
ind.f32	104.41	15.77	151	103.16	15.36	148	21.78	93.24	47
ind.f36	98.71	14.91	151	97.43	14.58	149	20.86	93.15	44
ind.m05	147.93	22.34	151	146.6	21.9	149	1.42	92.81	67
ind.m16	85.3	12.88	151	84.47	12.6	149	1.4	92.94	38
ind.m40	89.21	13.47	151	88.13	13.2	149	1.49	92.82	40
ind.m06	105.75	15.97	151	104.44	15.63	149	22.13	93.38	48
ind.m08	93.08	14.06	151	92.02	13.8	149	22.36	93.47	42
ind.m09	108.66	16.41	151	107.25	16.04	149	20.62	92.25	49
ind.m11	107.73	16.27	151	106.24	15.86	149	20.92	92.76	48
ind.m22	104.6	15.8	151	103.39	15.45	149	19	92.92	47
ind.m32	95.5	14.42	151	94.49	14.14	149	21.5	93.41	43
ind.m36	100.55	15.18	151	99.17	14.82	149	18.95	93.26	45

Table S2. BUSCOs results for the genome assembly of dwarf willow *Salix herbacea* based on the eudicots_odb10 database.

	Haplotype 1		Haplotype 2	
	No.	Prop.	No.	Prop.
Complete BUSCOS	2267	97.5 %	2285	98.3 %
Complete and single-copy BUSCOS	1944	83.6 %	1965	84.5 %
Complete and duplicated BUSCOs	323	13.9 %	320	13.8 %
Fragmented BUSCOs	7	0.3 %	7	0.3 %
Missing BUSCOs	52	2.2 %	34	1.4 %
Total BUSCO searched	2326		2326	

Table S3. Repeat annotation in the *Salix herbacea* genome assembly.

	Haplotype 1	Haplotype 2
Retroelements	20.88 %	20.85 %
SINEs	1.00 %	0.97 %
LINEs	0.70 %	0.73 %
LTR elements	19.18 %	19.15 %
DNA transposons	3.84 %	3.60 %
Unclassified	16.31 %	16.42 %
Small RNA	2.68 %	3.18 %
Total bases masked	49.01 %	49.33 %

Table S4. Published genomic data used.

Species	Data type	Version /Accession	Download from	Reference
A. thaliana	Annotation	Araport11	TAIR	[99]
P. deltoides	Assembly and annotation	JGI WV94 v2.1	Phytozome	[102]
P. trichocarpa	Assembly and annotation	JGI v4.1, AARH04000000	Phytozome	[83]
P. qiongdaoensis	Assembly and annotation	PRJCA007862	BIG Data Center	[51]
P. alba	Assembly and annotation	PRJCA002485	BIG Data Center	[8]
S. arbutifolia	Assembly and annotation	PRJCA016000	NCBI	[6]
S. brachista	Assembly and annotation	PRJNA472210	NCBI	[100]
S. dunnii	Assembly and annotation	PRJNA1136512	NCBI	[101]
S. exigua	Assembly	SE967M, PRJNA1009227	NCBI	[131]
S. koriyanagi	Assembly and annotation	SH3, PRJNA892598	NCBI	[131]
S. purpurea	Assembly and annotation	JGI v5.1	Phytozome	[33]
S.suchowensis	Assembly and annotation	XY12, PRJNA668632	NCBI	[132]
S.suchowensis	Assembly and annotation	P295, PRJNA892596	NCBI	[131]
S.suchowensis	Assembly and annotation	P63, PRJNA892593	NCBI	[131]
S. udensis	Assembly	04-BN-051, PRJNA892602	NCBI	[131]
S. viminalis	Assembly and annotation	JORR, PRJNA892601	NCBI	[131]
S. viminalis	Assembly and annotation	PRJEB15049	ENA	[133]
S.suchowensis	Illumina sequencing	P63, SRX14893761	NCBI	[131]
S.suchowensis	Illumina sequencing	P295, SRX14893739	NCBI	[131]
S.suchowensis	Illumina sequencing	P294, SRX14893691	NCBI	[131]
S. purpurea	Illumina sequencing	94006, SRX14893669	NCBI	[131]
S. purpurea	Illumina sequencing	94001, SRX14893727	NCBI	[131]
S. purpurea	Illumina sequencing	FishCreek, SRX2139496	NCBI	[33]
S. viminalis	Illumina sequencing	PRJNA1199981	NCBI	Generated by co-author

Table S5. Gene numbers and proportions of annotated sequences of the dwarf willow *Salix herbacea*, both for the entire chromosome (h1_chr15, h2_chr15), for the sex-linked region (W-SLR, Z-SLR) and for the five regions of the W-chromosome including two pseudoautosomal regions (PAR1, PAR2), two-female specific regions (FS1, FS2), and the inversion region (INV). Regions on the W-chromosome are ordered according to their position. Genes are divided into Z-W homologs (>80% identity) and Z or W specific genes (<60% identity), as well as according to their origin on chromosome 15 as conserved (detected on chr. 15 in each of three clades – 1: *S. purpurea*, *S. suchowensis* with ZW sex determination, 2: *S. arbutifolia*, *S. dunnii* with XY sex determination and 3: *P. qiongdaoensis* and *P. trichocarpa*), ancestral (detected on chr. 15 in at least one of these species) and specific (present on chr. 15 only in *S. herbacea*). Gene loss was estimated at the proportion of genes present divided by the joint number of conserved or ancestral genes across both haplotypes.

Chr./Regions	Length (Mb)	No. gene	Prop. gene	Prop. Repeat	Prop. LTR	Prop. Pseudogene	No. Z-W homologs	No. Z/W Specific genes	No. conserved genes	Prop. conserved genes lost	No. ancestral genes	Prop. ancestral genes lost
h1_chr15	15.8	1288	0.235	0.626	0.361	0.00468	1075	192	925	0.14	1145	0.17
h2_chr15	16.6	1488	0.235	0.628	0.342	0.00469	1115	263	1026	0.06	1250	0.11
W-SLR	8.4	426	0.146	0.794	0.516	0.00589	300	109	209	0.21	350	0.23
Z-SLR	8	477	0.153	0.798	0.487	0.00626	336	120	251	0.07	387	0.15
PAR1	3.1	319	0.292	0.499	0.218	0.00387						
FS1	2.8	171	0.188	0.718	0.419	0.0111						
INV	5	228	0.121	0.831	0.569	0.00268						
FS2	0.6	27	0.149	0.852	0.547	0.00727						
PAR2	4.3	543	0.367	0.392	0.161	0.00293						

Table S6. Summary of all the 138 genes with sex-specific variation on the W-SLR of *Salix herbacea*. Gene ID, location on the sex-linked region (FS1, FS2, INV), female-specific pattern (female-specific-polymorphisms (F-polym.): genes with fixed variants between female and male individuals from the study population; W-specific (W-sp.): genes specific to the W-haplotype but missing from Z-haplotype; W-specific-pseudogenized (W-sp.-P.): genes only annotated on the W haplotype but pseudogenized on the Z haplotype), as well as according to their origin on chromosome 15 as conserved (detected on chr. 15 in each of three clades – 1: *S. purpurea*, *S. suchowensis* with ZW sex determination, 2: *S. arbutifolia*, *S. dunnii* with XY sex determination and 3: *P. qiongdaoensis* and *P. trichocarpa*), ancestral (detected on chr. 15 in at least one of these species) and specific (present on chr. 15 only in *S. herbacea*) are given together with gene name and description, gene model according to the TAIR database.

GeneID	Region	Pattern	Homologs	Gene name	Description	Gene model
h1_chr15-_g23451	FS1	F-polym.	conserved	VQ20	VQ motif-containing protein	AT3G18360.1
h1_chr15+_g23452	FS1	F-polym.	conserved	ATSYTF	C2 domain-containing protein	AT3G18370.1
h1_chr15-_g23453	FS1	F-polym.	conserved	NAC058	NAC domain containing protein 58	AT3G18400.1
h1_chr15-_g23454	FS1	F-polym.	conserved	NAC058	NAC domain containing protein 58	AT3G18400.1
h1_chr15+_g23455	FS1	F-polym.	conserved	SecA	translocase subunit seca	AT1G68490.1
h1_chr15-_g23459	FS1	W-sp.	specific	ER	Leucine-rich receptor-like protein kinase family protein	AT2G26330.1
h1_chr15-_g23463	FS1	W-sp.	ancestral	None	Calcium-binding EF hand family protein	AT1G54530.1
h1_chr15+_g23464	FS1	W-sp.	conserved	WNK1	with no lysine (K) kinase 1	AT3G04910.1
h1_chr15-_g23467	FS1	W-sp.	ancestral	VTC2	GDP-L-galactose phosphorylase 1	AT4G26850.1
h1_chr15-_g23468	FS1	W-sp.	ancestral	AHP6	histidine phosphotransfer protein 6	AT1G80100.3
h1_chr15-_g23471	FS1	W-sp.	ancestral	RR22	response regulator 22	AT3G04280.1
h1_chr15-_g23476	FS1	W-sp.-P	conserved	SCD1	stomatal cytokinesis defective / SCD1 protein (SCD1)	AT1G49040.1
h1_chr15+_g23477	FS1	W-sp., F-polym.	ancestral	SCD1	stomatal cytokinesis defective / SCD1 protein (SCD1)	AT1G49040.1
h1_chr15-_g23478	FS1	W-sp.-P, F-polym.	specific	SCD1	stomatal cytokinesis defective / SCD1 protein (SCD1)	AT1G49040.1
h1_chr15+_g23479	FS1	F-polym.	ancestral	SCD1	stomatal cytokinesis defective / SCD1 protein (SCD1)	AT1G49040.1
h1_chr15-_g23481	FS1	W-sp.	specific	MAG1	Calcineurin-like metallo-phosphoesterase superfamily protein	AT3G47810.1
h1_chr15+_g23483	FS1	W-sp.	ancestral	RR24	response regulator 24	AT5G26594.1
h1_chr15-_g23484	FS1	W-sp.-P, F-polym.	conserved	SCD1	stomatal cytokinesis defective / SCD1 protein (SCD1)	AT1G49040.1
h1_chr15+_g23485	FS1	W-sp.-P	specific	SCD1	stomatal cytokinesis defective / SCD1 protein (SCD1)	AT1G49040.1
h1_chr15-_g23487	FS1	W-sp.	specific	NOF1	U3 small nucleolar RNA-associated protein	AT1G17690.1
h1_chr15-_g23488	FS1	W-sp.	ancestral	RPK1	receptor-like protein kinase 1	AT1G69270.1
h1_chr15-_g23492	FS1	W-sp.	specific	ER	Leucine-rich receptor-like protein kinase family protein	AT2G26330.1
h1_chr15-_g23495	FS1	W-sp.	ancestral	None	Calcium-binding EF hand family protein	AT1G54530.1
h1_chr15+_g23496	FS1	W-sp.	conserved	WNK1	with no lysine (K) kinase 1	AT3G04910.1
h1_chr15-_g23499	FS1	W-sp.	ancestral	VTC2	GDP-L-galactose phosphorylase 1	AT4G26850.1
h1_chr15-_g23500	FS1	W-sp.	ancestral	AHP6	histidine phosphotransfer protein 6	AT1G80100.3
h1_chr15+_g23502	FS1	W-sp.	ancestral			
h1_chr15-_g23505	FS1	W-sp.	ancestral	RR22	response regulator 22	AT3G04280.1
h1_chr15-_g23510	FS1	W-sp.-P	specific	SCD1	stomatal cytokinesis defective / SCD1 protein (SCD1)	AT1G49040.1
h1_chr15+_g23514	FS1	W-sp.	ancestral	RR24	response regulator 24	AT5G26594.1
h1_chr15-_g23515	FS1	W-sp.-P, F-polym.	conserved	SCD1	stomatal cytokinesis defective / SCD1 protein (SCD1)	AT1G49040.1
h1_chr15+_g23516	FS1	W-sp.-P, F-polym.	specific	SCD1	stomatal cytokinesis defective / SCD1 protein (SCD1)	AT1G49040.1

h1_chr15-_g23518	FS1	W-sp.	specific	APUM5	Encodes a member of the Arabidopsis Pumilio (APUM) proteins containing PUF domain (eight repeats of approximately 36 amino acids each).	AT3G20250.2
h1_chr15-_g23519	FS1	W-sp.	specific	NOF1	U3 small nucleolar RNA-associated protein	AT1G17690.1
h1_chr15-_g23520	FS1	W-sp.	ancestral	RPK1	receptor-like protein kinase 1	AT1G69270.1
h1_chr15-_g23522	FS1	W-sp.	ancestral	MEE29	helicase domain-containing protein	AT2G35340.1
h1_chr15-_g23525	FS1	W-sp.	specific	ER	Leucine-rich receptor-like protein kinase family protein	AT2G26330.1
h1_chr15-_g23528	FS1	W-sp.	ancestral	None	Calcium-binding EF hand family protein	AT1G54530.1
h1_chr15+_g23529	FS1	W-sp.	conserved	WNK1	with no lysine (K) kinase 1	AT3G04910.1
h1_chr15+_g23532	FS1	W-sp.	specific			
h1_chr15+_g23538	FS1	W-sp.	specific			
h1_chr15+_g23571	FS1	F-polym.	conserved	HIK	Encodes a kinesin HINKEL. Required for cytokinesis in pollen. Mutant has cytokinesis defects; seedling lethal.	AT1G18370.1
h1_chr15-_g23580	FS1	F-polym.	conserved	GGR	geranylgeranyl reductase	AT4G38460.1
h1_chr15-_g23583	FS1	F-polym.	conserved	PAPS1	poly(A) polymerase 1	AT1G17980.1
h1_chr15-_g23584	FS1	F-polym.	conserved	CP2	2-oxoglutarate (2OG) and Fe(II)-dependent oxygenase superfamily protein	AT3G18210.1
h1_chr15+_g23587	FS1	W-sp., F-polym.	ancestral	ADT2	arogenate dehydratase 2	AT3G07630.1
h1_chr15+_g23588	FS1	F-polym.	conserved	GRDP	Glycine-rich domain-containing protein-like	AT2G22660.2
h1_chr15-_g23589	FS1	F-polym.	conserved	CCT4	TCP-1/cpn60 chaperonin family protein	AT3G18190.1
h1_chr15-_g23599	FS1	W-sp., F-polym.	ancestral	FATB	fatty acyl-ACP thioesterases B	AT1G08510.1
h1_chr15-_g23600	FS1	F-polym.	conserved	GT61	Glycosyltransferase family 61 protein	AT3G18170.2
h1_chr15-_g23601	FS1	F-polym.	ancestral	TPR	Tetratricopeptide repeat (TPR)-like superfamily protein	AT5G62370.1
h1_chr15+_g23602	FS1	F-polym.	conserved	DEAD/H	DEA(D/H)-box RNA helicase family protein	AT1G48650.2
h1_chr15-_g23603	FS1	W-sp.	ancestral			
h1_chr15+_g23604	FS1	W-sp., F-polym.	specific	Q1PEN6	Gag-Pol-related retrotransposon family protein	AT3G21000.1
h1_chr15+_g23605	FS1	F-polym.	ancestral	CRK8	cysteine-rich RLK (RECEPTOR-like protein kinase) 8	AT4G23160.2
h1_chr15-_g23606	FS1	F-polym.	specific	GT61	Glycosyltransferase family 61 protein	AT3G18170.2
h1_chr15-_g23607	FS1	F-polym.	specific	GT61	Glycosyltransferase family 61 protein	AT3G18180.1
h1_chr15+_g23650	INV	W-sp.	ancestral			
h1_chr15+_g23658	INV	W-sp.	specific			
h1_chr15-_g23671	INV	W-sp.	conserved			
h1_chr15-_g23673	INV	W-sp.-P	specific			
h1_chr15+_g23680	INV	W-sp.	conserved	None	Haloacid dehalogenase-like hydrolase (HAD) superfamily protein	AT4G26190.1
h1_chr15-_g23690	INV	W-sp.	ancestral			
h1_chr15-_g23691	INV	W-sp.	ancestral	SAUR72	SAUR-like auxin-responsive protein family	AT3G12830.1
h1_chr15-_g23692	INV	W-sp.	ancestral	HTD1	Transducin family protein / WD-40 repeat family protein	AT2G19540.1
h1_chr15-_g23693	INV	W-sp.	ancestral			
h1_chr15-_g23694	INV	W-sp.	specific	SAUR72	SAUR-like auxin-responsive protein family	AT3G12830.1
h1_chr15-_g23695	INV	W-sp.	specific	SOK1	UPSTREAM OF FLC protein (DUF966)	AT1G05577.1
h1_chr15-_g23696	INV	W-sp.	specific	SOK1	UPSTREAM OF FLC protein (DUF966)	AT1G05577.1
h1_chr15-_g23697	INV	W-sp.	ancestral			
h1_chr15-_g23698	INV	W-sp.	specific	SAUR72	SAUR-like auxin-responsive protein family	AT3G12830.1
h1_chr15-_g23699	INV	W-sp.	specific	SOK1	UPSTREAM OF FLC protein (DUF966)	AT1G05577.1
h1_chr15-_g23700	INV	W-sp.	specific	SOK1	UPSTREAM OF FLC protein (DUF966)	AT1G05577.1
h1_chr15-_g23701	INV	W-sp.	ancestral			
h1_chr15-_g23702	INV	W-sp.	specific	SAUR72	SAUR-like auxin-responsive protein family	AT3G12830.1
h1_chr15-_g23703	INV	W-sp.	ancestral	HTD1	Transducin family protein / WD-40 repeat family protein	AT2G19540.1
h1_chr15+_g23704	INV	W-sp.	ancestral	ATG4A	Peptidase family C54 protein	AT2G44140.1
h1_chr15-_g23705	INV	W-sp.	ancestral	SAP130b	Cleavage and polyadenylation specificity factor (CPSF) A subunit protein	AT3G55220.1
h1_chr15-_g23706	INV	W-sp.	ancestral	SAP130b	Cleavage and polyadenylation specificity factor (CPSF) A subunit protein	AT3G55220.1

h1_chr15-_g23707	INV	W-sp.	ancestral	SAP130b	Cleavage and polyadenylation specificity factor (CPSF) A subunit protein	AT3G55220.1
h1_chr15+_g23708	INV	W-sp.	ancestral	ATG4A	Peptidase family C54 protein	AT2G44140.1
h1_chr15-_g23709	INV	W-sp.	specific	None	NAD(P)-binding Rossmann-fold superfamily protein	AT2G29170.1
h1_chr15+_g23710	INV	W-sp.	ancestral	SAP130b	Cleavage and polyadenylation specificity factor (CPSF) A subunit protein	AT3G55220.1
h1_chr15+_g23711	INV	W-sp.	ancestral	SAP130b	Cleavage and polyadenylation specificity factor (CPSF) A subunit protein	AT3G55220.1
h1_chr15-_g23712	INV	W-sp.	ancestral	ATG4A	Peptidase family C54 protein	AT2G44140.1
h1_chr15+_g23713	INV	W-sp.	ancestral	SAP130b	Cleavage and polyadenylation specificity factor (CPSF) A subunit protein	AT3G55220.1
h1_chr15+_g23714	INV	W-sp.	ancestral	SAP130b	Cleavage and polyadenylation specificity factor (CPSF) A subunit protein	AT3G55220.1
h1_chr15+_g23715	INV	W-sp.	ancestral	SAP130b	Cleavage and polyadenylation specificity factor (CPSF) A subunit protein	AT3G55220.1
h1_chr15-_g23716	INV	W-sp.	ancestral	ATG4A	Peptidase family C54 protein	AT2G44140.1
h1_chr15+_g23717	INV	W-sp.	ancestral	ASHH3	histone-lysine N-methyltransferase ASHH3	AT2G44150.1
h1_chr15+_g23718	INV	W-sp.	ancestral	BRR2c	U5 small nuclear ribonucleoprotein helicase	AT5G61140.2
h1_chr15-_g23720	INV	W-sp.-P	specific			
h1_chr15+_g23729	INV	W-sp.	specific			
h1_chr15-_g23734	INV	W-sp.	ancestral	SAP130b	Cleavage and polyadenylation specificity factor (CPSF) A subunit protein	AT3G55220.1
h1_chr15-_g23735	INV	W-sp.	ancestral	SAP130b	Cleavage and polyadenylation specificity factor (CPSF) A subunit protein	AT3G55220.1
h1_chr15-_g23736	INV	W-sp.	ancestral	SAP130b	Cleavage and polyadenylation specificity factor (CPSF) A subunit protein	AT3G55220.1
h1_chr15+_g23740	INV	W-sp.	ancestral	None	hypothetical protein	AT1G21560.2
h1_chr15-_g23741	INV	W-sp.	ancestral	NdhM	subunit NDH-M of NAD(P)H:plastoquinone dehydrogenase complex	AT4G37925.1
h1_chr15+_g23742	INV	W-sp.	ancestral			
h1_chr15+_g23743	INV	W-sp.	specific	None	L-aminoadipate-semialdehyde dehydrogenase-phosphopantetheinyl transferase	AT2G02765.1
h1_chr15+_g23744	INV	W-sp.	ancestral	None	RNA-binding (RRM/RBD/RNP motifs) family protein	AT5G46840.1
h1_chr15-_g23745	INV	W-sp.	ancestral	None	SNARE associated Golgi protein family	AT4G17790.1
h1_chr15+_g23746	INV	W-sp.	ancestral	None	RNA-binding (RRM/RBD/RNP motifs) family protein	AT5G46840.1
h1_chr15-_g23747	INV	W-sp.	ancestral	None	SNARE associated Golgi protein family	AT4G17790.1
h1_chr15+_g23748	INV	W-sp.	ancestral	None	RNA-binding (RRM/RBD/RNP motifs) family protein	AT5G46840.1
h1_chr15-_g23749	INV	W-sp.	ancestral	None	SNARE associated Golgi protein family	AT4G17790.1
h1_chr15+_g23750	INV	W-sp.	ancestral	None	RNA-binding (RRM/RBD/RNP motifs) family protein	AT5G46840.1
h1_chr15+_g23751	INV	W-sp.	specific			
h1_chr15-_g23752	INV	W-sp.	ancestral	BRR2c	U5 small nuclear ribonucleoprotein helicase	AT5G61140.2
h1_chr15-_g23753	INV	W-sp.	ancestral	BRR2c	U5 small nuclear ribonucleoprotein helicase	AT5G61140.2
h1_chr15+_g23776	INV	W-sp.	specific			
h1_chr15+_g23781	INV	W-sp.	specific			
h1_chr15-_g23796	INV	W-sp.	specific	AtHIGD2	Hypoxia-responsive family protein	AT5G27760.1
h1_chr15+_g23816	INV	W-sp.	specific	TRA1a	Phosphatidylinositol 3- and 4-kinase family protein with FAT domain-containing protein	AT2G17930.1
h1_chr15+_g23818	INV	W-sp.	specific	TRA1b	phosphotransferases/inositol or phosphatidylinositol kinase	AT4G36080.1
h1_chr15+_g23821	INV	W-sp.-P	specific	None	DNA-binding storekeeper protein-related transcriptional regulator	AT1G61730.1
h1_chr15+_g23823	INV	W-sp.-P	ancestral			
h1_chr15-_g23827	INV	W-sp.-P	ancestral	ASPG1	Eukaryotic aspartyl protease family protein	AT3G18490.1
h1_chr15+_g23828	INV	W-sp.-P	specific			
h1_chr15+_g23836	INV	F-polym.	conserved	ENODL6	Early nodulin-like protein 6	AT1G48940.1
h1_chr15-_g23837	INV	W-sp.-P	specific			
h1_chr15+_g23838	INV	F-polym.	conserved	ARID	AT-rich interactive domain protein	AT1G73885.1
h1_chr15-_g23839	INV	F-polym.	conserved	LPTG10	Bifunctional inhibitor/lipid-transfer protein/seed storage 2S albumin superfamily protein	AT1G73890.1
h1_chr15+_g23840	INV	F-polym.	conserved	ALMT9	aluminum-activated malate transporter 9	AT3G18440.1
h1_chr15-_g23841	FS2	F-polym.	conserved	APCB1	Eukaryotic aspartyl protease family protein	AT1G49050.1
h1_chr15-_g23842	FS2	F-polym.	conserved	ARP4	actin-related protein 4	AT1G18450.1

h1_chr15-_g23843	FS2	F-polym.	specific	MEE29	maternal effect embryo arrest 29, DEAH RNA helicase homolog PRP2	AT2G35340.1
h1_chr15-_g23844	FS2	W-sp., F-polym.	specific	GSTT3	glutathione S-transferase THETA 3	AT5G41220.1
h1_chr15-_g23846	FS2	F-polym.	conserved			
h1_chr15-_g23847	FS2	W-sp.-P, F-polym.	conserved	CRF9	cytokinin response factor 9	AT1G49120.1
h1_chr15-_g23848	FS2	W-sp.-P, F-polym.	conserved	CRF9	cytokinin response factor 9	AT1G49120.1
h1_chr15+_g23849	FS2	W-sp., F-polym.	ancestral	NOF1	U3 small nucleolar RNA-associated protein	AT1G17690.1
h1_chr15-_g23850	FS2	W-sp., F-polym.	ancestral	SWEETIE	HEAT repeat-containing protein	AT1G67140.2
h1_chr15-_g23851	FS2	W-sp., F-polym.	specific	SWEETIE	HEAT repeat-containing protein	AT1G67140.2
h1_chr15-_g23852	FS2	W-sp., F-polym.	specific	MiP1	Leucine-zipper of ternary complex factor MIP1	PF14389
h1_chr15-_g23853	FS2	F-polym.	conserved	BBX15	B-box type zinc finger protein with CCT domain	AT1G25440.1
h1_chr15-_g23854	FS2	F-polym.	conserved	ARP4	actin-related protein 4	AT1G18450.1
h1_chr15+_g23855	FS2	F-polym.	conserved	RR5	response regulator 5	AT3G48100.1

Table S7. Functional summary of 44 genes with female-specific polymorphisms (F-polym.; Table S6) located on the W-SLR of *Salix herbacea*. Gene ID, location on the female-specific regions (FS1, FS2), information on female-specific pattern (INDEL: female-specific insertion or deletion; SNP: female-specific polymorphism; disruptive mutations are indicated with an asterisk), information on homologs on chromosome 15 in other *Salix* species (conserved: detected on chr. 15 in three clades – clade 1: *S. purpurea*, *S. suchowensis* with ZW sex determination, clade 2: *S. arbutifolia*, *S. dunnii* with XY sex determination and clade 3: *P. qiongdaoensis* and *P. trichocarpa*, ancestral: detected on chr. 15 in at least one of these six species; and specific: present on chr. 15 only in *S. herbacea*) and information on whether the sex-specific pattern are shared with three other *Salix* species (svim: only shared with *S. viminalis*, all: shared with *S. viminalis*, *S. purpurea* and *S. suchowensis*, none: not shared) are given together with gene name and description, gene model, putative function according to the TAIR gene model and a function category (female function, male function, or general flowering formation function (flower)) and references.

Gene ID	Region	Female-specific pattern	Homologs	Common alleles	Gene Name	Gene description	Gene model	Putative function	Category	Reference
h1_chr15-_g23451	FS1	SNP	conserved	none	VQ20	VQ motif-containing protein	AT3G18360.1	stress responses and reproductive development	stress	[138]
h1_chr15+_g23452	FS1	SNP	conserved	none	ATSYTF	C2 domain-containing protein	AT3G18370.1	abiotic stress response	stress	[139]
h1_chr15-_g23453	FS1	INDEL	conserved	none	NAC058	NAC domain containing protein 58	AT3G18400.1	control of flowering time and cold response	flower	[140]
h1_chr15-_g23454	FS1	SNP	conserved	svim	NAC058	NAC domain containing protein 58	AT3G18400.1	control of flowering time and cold response	flower	[140]
h1_chr15+_g23455	FS1	INDEL	conserved	svim	SecA	translocase subunit seca	AT1G68490.1	photosynthetic development	growth	[141]
h1_chr15+_g23477	FS1	INDEL	ancestral	none	SCD1	stomatal cytokinesis defective / SCD1 protein (SCD1)	AT1G49040.1	seedling growth, root elongation and flower morphogenesis	flower	[45]
h1_chr15-_g23478	FS1	INDEL	specific	none	SCD1	stomatal cytokinesis defective / SCD1 protein (SCD1)	AT1G49040.1	seedling growth, root elongation and flower morphogenesis	flower	[45]
h1_chr15+_g23479	FS1	INDEL	ancestral	none	SCD1	stomatal cytokinesis defective / SCD1 protein (SCD1)	AT1G49040.1	seedling growth, root elongation and flower morphogenesis	flower	[45]
h1_chr15-_g23484	FS1	INDEL	conserved	none	SCD1	stomatal cytokinesis defective / SCD1 protein (SCD1)	AT1G49040.1	seedling growth, root elongation and flower morphogenesis	flower	[45]
h1_chr15-_g23515	FS1	INDEL	conserved	none	SCD1	stomatal cytokinesis defective / SCD1 protein (SCD1)	AT1G49040.1	seedling growth, root elongation and flower morphogenesis	flower	[45]
h1_chr15+_g23516	FS1	INDEL	specific	none	SCD1	stomatal cytokinesis defective / SCD1 protein (SCD1)	AT1G49040.1	seedling growth, root elongation and flower morphogenesis	flower	[45]
h1_chr15+_g23571	FS1	SNP	conserved	svim	HIK	ARABIDOPSIS NPK1-ACTIVATING KINESIN 1	AT1G18370.1	required for cytokinesis in pollen	male	[39]
h1_chr15-_g23580	FS1	SNP	conserved	none	GGR	geranylgeranyl reductase	AT4G38460.1	reproduction and stress response	stress	[142]
h1_chr15-_g23583	FS1	SNP	conserved	none	PAPS1	poly(A) polymerase 1	AT1G17980.1	mediate pollen maturation by regulating sperm cell differentiation	male	[40]
h1_chr15-_g23584	FS1	SNP	conserved	none	CP2	2-oxoglutarate (2OG) and Fe(II)-dependent oxygenase superfamily protein	AT3G18210.1	epigenetic repression of flowering genes	flower	[143]

h1_chr15+_g23587	FS1	INDEL	ancestral	none	ADT2	arogenate dehydratase 2	AT3G07630.1	seed development	growth	[144]
h1_chr15+_g23588	FS1	SNP	conserved	none	GRDP	Glycine-rich domain-containing protein-like	AT2G22660.2	stress responses	stress	[145]
h1_chr15-_g23589	FS1	SNP	conserved	none	CCT4	TCP-1/cpn60 chaperonin family protein	AT3G18190.1	tubulin folding, essential for growth	growth	[146]
h1_chr15-_g23599	FS1	SNP	ancestral	none	FATB	fatty acyl-ACP thioesterases B	AT1G08510.1	a major determinant of saturated fatty acid synthesis	growth	[147]
h1_chr15-_g23600	FS1	INDEL, SNP	conserved	none	GT61	Glycosyltransferase family 61 protein	AT3G18170.2	arabinoxylan biosynthesis	growth	[148]
h1_chr15-_g23601	FS1	INDEL, SNP	ancestral	none	TPR	Tetratricopeptide repeat (TPR)-like superfamily protein	AT5G62370.1	hormone responses and regulation of flowering	flower	[149]
h1_chr15+_g23602	FS1	INDEL, SNP	conserved	none	DEAD/H	DEA(D/H)-box RNA helicase family protein	AT1G48650.2	stress responses and regulates flowering time	stress	[150]
h1_chr15+_g23604	FS1	INDEL, SNP	specific	svim	Q1PEN6	Gag-Pol-related retrotransposon family protein	AT3G21000.1	LTR retrotransposition	growth	[151]
h1_chr15+_g23605	FS1	INDEL	ancestral	svim	CRK8	cysteine-rich RLK (RECEPTOR-like protein kinase) 8	AT4G23160.2	stress responses	stress	[152]
h1_chr15-_g23606	FS1	INDEL, SNP	specific	none	GT61	Glycosyltransferase family 61 protein	AT3G18170.2	arabinoxylan biosynthesis	growth	[148]
h1_chr15-_g23607	FS1	SNP	specific	none	GT61	Glycosyltransferase family 61 protein	AT3G18180.1	arabinoxylan biosynthesis	growth	[148]
h1_chr15+_g23836	FS2	SNP	conserved	none	ENODL6	Early nodulin-like protein 6	AT1G48940.1	seedling growth, pollen tube elongation, embryogenesis and cell proliferation	male	[41]
h1_chr15+_g23838	FS2	SNP	conserved	none	ARID	AT-rich interactive domain protein	AT1G73885.1	pollen tube growth	male	[42]
h1_chr15-_g23839	FS2	SNP	conserved	none	LPTG10	Bifunctional inhibitor/lipid-transfer protein/seed storage 2S albumin superfamily protein	AT1G73890.1	resistance against abiotic stress	stress	[153]
h1_chr15+_g23840	FS2	SNP	conserved	none	ALMT9	aluminum-activated malate transporter 9	AT3G18440.1	a dicarboxylic acid transporter mediate malate transportation	growth	[154]
h1_chr15-_g23841	FS2	SNP	conserved	none	APCB1	Eukaryotic aspartyl protease family protein	AT1G49050.1	stress responses and reproductive development	stress	[155]
h1_chr15-_g23842	FS2	SNP	conserved	svim	ARP4	actin-related protein 4	AT1G18450.1	male sterility due to defects in pollen and anther	male	[38]
h1_chr15-_g23843	FS2	SNP*	specific	none	MEE29	maternal effect embryo arrest 29, DEAH RNA helicase homolog PRP2	AT2G35340.1	female gametophyte development and function	female	[156]
h1_chr15-_g23844	FS2	SNP*	specific	none	GSTT3	glutathione S-transferase THETA 3, No apical meristem-associated C-terminal domain	AT5G41220.1	control of flowering time and cold response	flower	[140]
h1_chr15-_g23846	FS2	SNP	conserved	none	-	-				
h1_chr15-_g23847	FS2	SNP	conserved	none	CRF9	cytokinin response factor 9	AT1G49120.1	a repressor of cytokinin ARR6 during reproductive development	growth	[37]
h1_chr15-_g23848	FS2	SNP*	conserved	svim	CRF9	cytokinin response factor 9	AT1G49120.1	a repressor of cytokinin ARR6 during reproductive development	growth	[37]

h1_chr15+_g23849	FS2	INDEL	ancestral	none	NOF1	U3 small nucleolar RNA-associated protein, DEAD-box RNA helicase-like	AT1G17690.1	female gametogenesis and embryo development	female	[44]
h1_chr15-_g23850	FS2	INDEL	ancestral	none	SWEETIE	HEAT repeat-containing protein	AT1G67140.2	growth and development phenotypes, such asdwarfism, lancet-shaped leaves, early senescence and flower sterility.	growth	[46]
h1_chr15-_g23851	FS2	INDEL	specific	none	SWEETIE	HEAT repeat-containing protein	AT1G67140.2	growth and development phenotypes, such asdwarfism, lancet-shaped leaves, early senescence and flower sterility.	growth	[46]
h1_chr15-_g23852	FS2	INDEL, SNP	specific	svim	MiP1	Leucine-zipper of ternary complex factor MIP1	PF14389	regulate flowering time, interacting with B-box zinc finger protein	flower	[47]
h1_chr15-_g23853	FS2	SNP	conserved	none	BBX15	B-box type zinc finger protein with CCT domain	AT1G25440.1	regulates flowering time and abiotic stress	flower	[36]
h1_chr15-_g23854	FS2	INDEL, SNP	conserved	all	ARP4	actin-related protein 4	AT1G18450.1	male sterility due to defects in pollen and anther	male	[38]
h1_chr15+_g23855	FS2	SNP	conserved	none	RR5	response regulator 5	AT3G48100.1	response to cytokinin	growth	[49]

Table S8. Summary of genes specific for the Z-SLR haplotype of *Salix herbacea*. Gene ID, location (Z-SLR), sex-specific pattern (Z-specific: genes specific to the Z haplotype but missing from W haplotype; Z-specific-pseudogenized (Z-sp.-P): genes only annotated on the Z haplotype but pseudogenized on the W haplotype), as well as according to their origin on chromosome 15 (conserved: detected on chr. 15 in three clades – clade 1: *S. purpurea*, *S. suchowensis* with ZW sex determination, clade 2: *S. arbutifolia*, *S. dunnii* with XY sex determination and clade 3: *P. qiongdaoensis* and *P. trichocarpa*, ancestral: detected on chr. 15 in at least one of these six species; and specific: present on chr. 15 only in *S. herbacea*) are given together with gene name and description, gene model according to the TAIR database.

GeneID	Region	Pattern	Homologs	Gene name	Description	Gene model
h2_chr15-_g23435	Z-SLR	Z-specific	ancestral	MEE21	PIN domain-like family protein	AT2G34570.1
h2_chr15+_g23439	Z-SLR	Z-sp.-P		SMA1	P-loop containing nucleoside triphosphate hydrolases superfamily protein	AT2G33730.1
h2_chr15+_g23445	Z-SLR	Z-specific		JASON	aspartyl/glutamyl-tRNA(Asn/Gln) amidotransferase subunit	AT1G06660.1
h2_chr15-_g23446	Z-SLR	Z-specific		JASON	aspartyl/glutamyl-tRNA(Asn/Gln) amidotransferase subunit	AT1G06660.1
h2_chr15+_g23454	Z-SLR	Z-sp.-P	ancestral	SMA1	P-loop containing nucleoside triphosphate hydrolases superfamily protein	AT2G33730.1
h2_chr15+_g23457	Z-SLR	Z-specific	ancestral	None	Gag-Pol-related retrotransposon family protein	AT3G21000.1
h2_chr15-_g23471	Z-SLR	Z-specific	ancestral	None	RNA-directed DNA polymerase (reverse transcriptase)-related family protein	AT4G10613.1
h2_chr15+_g23472	Z-SLR	Z-specific		CIP1	COP1-interactive protein 1	AT5G41790.1
h2_chr15-_g23473	Z-SLR	Z-specific	ancestral	SS3	strictosidine synthase 3	AT1G74000.1
h2_chr15+_g23480	Z-SLR	Z-sp.-P	ancestral	SMA1	P-loop containing nucleoside triphosphate hydrolases superfamily protein	AT2G33730.1
h2_chr15-_g23483	Z-SLR	Z-sp.-P				
h2_chr15-_g23487	Z-SLR	Z-specific	ancestral	None	Copia-like polyprotein/retrotransposon	AT5G48050.1
h2_chr15-_g23489	Z-SLR	Z-specific		MAP70-1	microtubule-associated proteins 70-1	AT1G68060.1
h2_chr15-_g23493	Z-SLR	Z-specific	ancestral	VPS26C	Vacuolar protein sorting-associated protein 26	AT1G48550.1
h2_chr15+_g23495	Z-SLR	Z-specific				
h2_chr15+_g23496	Z-SLR	Z-sp.-P	ancestral	None	zinc ion binding / nucleic acid binding protein	AT2G01050.1
h2_chr15+_g23497	Z-SLR	Z-specific	ancestral	None	DNAse I-like superfamily protein	AT1G40390.1
h2_chr15+_g23510	Z-SLR	Z-specific				
h2_chr15-_g23524	Z-SLR	Z-sp.-P	ancestral			
h2_chr15-_g23531	Z-SLR	Z-specific	ancestral	None	Copia-like polyprotein/retrotransposon	AT1G48720.1
h2_chr15+_g23536	Z-SLR	Z-specific		None	Ubiquitin-conjugating enzyme family protein	AT2G18600.1

h2_chr15+_g23537	Z-SLR	Z-specific	ancestral	TRS120	TRS120	AT5G11040.1
h2_chr15+_g23540	Z-SLR	Z-specific	ancestral	RABA6a	RAB GTPase homolog A6A	AT1G73640.1
h2_chr15+_g23541	Z-SLR	Z-specific	ancestral	GC2	golgin Putative 2	AT1G18190.1
h2_chr15-_g23542	Z-SLR	Z-specific	ancestral	None	3-oxo-5-alpha-steroid 4-dehydrogenase (DUF1295)	AT1G18180.1
h2_chr15-_g23543	Z-SLR	Z-specific	ancestral	SIS8	protein tyrosine kinase family protein	AT1G73660.1
h2_chr15-_g23545	Z-SLR	Z-specific	ancestral	ALDH10A8	aldehyde dehydrogenase 10A8	AT1G74920.1
h2_chr15-_g23565	Z-SLR	Z-specific	ancestral	HON5	high mobility group A5	AT1G48620.1
h2_chr15+_g23566	Z-SLR	Z-specific	ancestral	MPK15	MAP kinase 15	AT1G73670.1
h2_chr15+_g23567	Z-SLR	Z-specific	ancestral	LAC1	laccase 1	AT1G18140.1
h2_chr15+_g23568	Z-SLR	Z-specific	ancestral	None	GPI-anchored protein	AT3G18050.1
h2_chr15+_g23569	Z-SLR	Z-specific				
h2_chr15-_g23570	Z-SLR	Z-specific	ancestral	E12A11	PEBP (phosphatidylethanolamine-binding protein) family protein	AT1G18100.1
h2_chr15+_g23572	Z-SLR	Z-specific	ancestral	None	transducin family protein / WD-40 repeat family protein	AT3G18060.1
h2_chr15+_g23573	Z-SLR	Z-specific	ancestral	BGLU44	B-S glucosidase 44	AT3G18080.1
h2_chr15-_g23574	Z-SLR	Z-specific	ancestral	None	5-3 exonuclease family protein	AT1G18090.1
h2_chr15+_g23575	Z-SLR	Z-specific	ancestral	MYB4R1	myb domain protein 4r1	AT3G18100.1
h2_chr15-_g23576	Z-SLR	Z-specific	ancestral	ATARCA	Transducin/WD40 repeat-like superfamily protein	AT1G18080.1
h2_chr15+_g23577	Z-SLR	Z-specific	ancestral	PEX3	peroxin 3	AT1G48635.2
h2_chr15+_g23578	Z-SLR	Z-specific	ancestral	MOS4	modifier of snc1,4	AT3G18165.1
h2_chr15-_g23579	Z-SLR	Z-specific				
h2_chr15-_g23580	Z-SLR	Z-specific				
h2_chr15+_g23581	Z-SLR	Z-specific				
h2_chr15+_g23582	Z-SLR	Z-specific				
h2_chr15-_g23583	Z-SLR	Z-specific	ancestral	GSTU4	glutathione S-transferase tau 4	AT2G29460.1
h2_chr15+_g23595	Z-SLR	Z-specific		SDE5	silencing defective 5	AT3G15390.1
h2_chr15+_g23597	Z-SLR	Z-specific	ancestral	None	F-box/RNI-like/FBD-like domains-containing protein	AT5G44980.1
h2_chr15-_g23598	Z-SLR	Z-specific	ancestral	AtFDR1	F-box family protein	AT1G57790.1
h2_chr15-_g23599	Z-SLR	Z-sp.-P	ancestral	CYP81D11	Cytochrome P450 superfamily protein	AT3G28740.1

h2_chr15-_g23600	Z-SLR	Z-specific	ancestral	RAH1	F-box family protein	AT5G27920.1
h2_chr15+_g23605	Z-SLR	Z-specific		ORC2	origin recognition complex second largest subunit 2	AT2G37560.1
h2_chr15-_g23607	Z-SLR	Z-specific	ancestral			
h2_chr15+_g23611	Z-SLR	Z-specific	ancestral	None	cysteine-rich RECEPTOR-like kinase	AT4G23160.2
h2_chr15-_g23619	Z-SLR	Z-specific		None	DEA(D/H)-box RNA helicase family protein	AT1G48650.2
h2_chr15+_g23624	Z-SLR	Z-sp.-P	ancestral	None	F-box family protein	AT3G56470.1
h2_chr15+_g23625	Z-SLR	Z-sp.-P		RAE1	RNI-like superfamily protein	AT5G01720.1
h2_chr15-_g23628	Z-SLR	Z-sp.-P	ancestral	CYP81D11	Cytochrome P450 superfamily protein	AT3G28740.1
h2_chr15-_g23629	Z-SLR	Z-sp.-P	ancestral	RAE1	RNI-like superfamily protein	AT5G01720.1
h2_chr15+_g23635	Z-SLR	Z-sp.-P	ancestral	None	Glycosyltransferase family 61 protein	AT3G18180.1
h2_chr15-_g23636	Z-SLR	Z-specific	ancestral			
h2_chr15+_g23638	Z-SLR	Z-specific	ancestral	None	F-box/RNI-like/FBD-like domains-containing protein	AT5G44980.1
h2_chr15+_g23643	Z-SLR	Z-specific	ancestral	None	F-box/RNI-like superfamily protein	AT3G59200.1
h2_chr15+_g23646	Z-SLR	Z-specific	ancestral	None	Glycosyltransferase family 61 protein	AT3G18170.2
h2_chr15-_g23651	Z-SLR	Z-sp.-P	ancestral	None	F-box family protein	AT3G18720.1
h2_chr15-_g23652	Z-SLR	Z-sp.-P	ancestral	CYP81D8	cytochrome P450, family 81, subfamily D, polypeptide 8	AT4G37370.1
h2_chr15-_g23653	Z-SLR	Z-specific	ancestral	RAE1	RNI-like superfamily protein	AT5G01720.1
h2_chr15-_g23654	Z-SLR	Z-sp.-P	ancestral			
h2_chr15-_g23661	Z-SLR	Z-specific		None	DEA(D/H)-box RNA helicase family protein	AT1G48650.2
h2_chr15+_g23664	Z-SLR	Z-sp.-P	ancestral	PISTILLATA	K-box region and MADS-box transcription factor family protein	AT5G20240.1
h2_chr15+_g23666	Z-SLR	Z-sp.-P				
h2_chr15-_g23673	Z-SLR	Z-sp.-P				
h2_chr15+_g23682	Z-SLR	Z-sp.-P	ancestral	CYP77A5P	cytochrome P450, family 77, subfamily A, polypeptide 5 pseudogene	AT3G18270.1
h2_chr15+_g23689	Z-SLR	Z-sp.-P	ancestral			
h2_chr15+_g23700	Z-SLR	Z-specific				
h2_chr15+_g23704	Z-SLR	Z-specific				
h2_chr15+_g23708	Z-SLR	Z-specific				
h2_chr15+_g23712	Z-SLR	Z-specific				

h2_chr15+_g23714	Z-SLR	Z-specific				
h2_chr15-_g23725	Z-SLR	Z-sp.-P				
h2_chr15-_g23726	Z-SLR	Z-specific				
h2_chr15-_g23727	Z-SLR	Z-specific		NDHB.2	NADH-quinone oxidoreductase protein	ATCG01250.1
h2_chr15-_g23728	Z-SLR	Z-specific				
h2_chr15+_g23744	Z-SLR	Z-sp.-P		ASPG1	Eukaryotic aspartyl protease family protein	AT3G18490.1
h2_chr15+_g23746	Z-SLR	Z-sp.-P	ancestral	None	Eukaryotic aspartyl protease family protein	AT1G25510.1
h2_chr15-_g23751	Z-SLR	Z-specific	ancestral	None	Zinc knuckle (CCHC-type) family protein	AT2G15180.1
h2_chr15-_g23752	Z-SLR	Z-specific				
h2_chr15-_g23753	Z-SLR	Z-specific	ancestral			
h2_chr15+_g23758	Z-SLR	Z-specific	ancestral	None	Galactose mutarotase-like superfamily protein	AT5G57330.1
h2_chr15-_g23793	Z-SLR	Z-sp.-P	ancestral	None	DNAse I-like superfamily protein	AT1G40390.1
h2_chr15-_g23805	Z-SLR	Z-sp.-P	ancestral	ENO1	enolase 1	AT1G74030.1
h2_chr15+_g23812	Z-SLR	Z-specific				
h2_chr15+_g23813	Z-SLR	Z-specific				
h2_chr15-_g23820	Z-SLR	Z-specific	ancestral			
h2_chr15-_g23836	Z-SLR	Z-sp.-P	ancestral			
h2_chr15+_g23844	Z-SLR	Z-specific				
h2_chr15+_g23846	Z-SLR	Z-specific				
h2_chr15+_g23869	Z-SLR	Z-specific		CPSRP54	chloroplast signal recognition particle 54 kDa subunit	AT5G03940.1
h2_chr15+_g23870	Z-SLR	Z-sp.-P	ancestral	CPK30	calcium-dependent protein kinase 30	AT1G74740.1
h2_chr15+_g23871	Z-SLR	Z-specific	ancestral	None	DNAJ heat shock family protein	AT3G47940.1
h2_chr15-_g23872	Z-SLR	Z-specific	ancestral	TIG1	trigger factor type chaperone family protein	AT5G55220.1
h2_chr15-_g23873	Z-SLR	Z-specific	ancestral	AR192	Co-chaperone GrpE family protein	AT4G26780.1
h2_chr15+_g23874	Z-SLR	Z-specific	ancestral	AtNITR2	Integral membrane HPP family protein	AT5G62720.1
h2_chr15-_g23875	Z-SLR	Z-specific	ancestral	HB-1	homeobox 1	AT3G01470.1
h2_chr15+_g23876	Z-SLR	Z-specific	ancestral			
h2_chr15-_g23877	Z-SLR	Z-specific	ancestral	RIQ2	transmembrane protein, putative (DUF1118)	AT1G74730.1

h2_chr15-_g23878	Z-SLR	Z-specific	ancestral	None	hypothetical protein	AT5G62750.1
h2_chr15-_g23879	Z-SLR	Z-specific	ancestral	HSP17.8	HSP20-like chaperones superfamily protein	AT1G07400.1
h2_chr15+_g23880	Z-SLR	Z-specific		ATN	cyclin family	AT3G05330.1
h2_chr15-_g23881	Z-SLR	Z-specific	ancestral	None	P-loop containing nucleoside triphosphate hydrolases superfamily protein	AT5G62760.1
h2_chr15+_g23882	Z-SLR	Z-specific	ancestral	TRZ1	tRNAse Z1	AT1G74700.1
h2_chr15-_g23883	Z-SLR	Z-sp.-P	ancestral	PCP2	hypothetical protein	AT1G18850.1
h2_chr15+_g23885	Z-SLR	Z-specific		ABHD11	alpha/beta-Hydrolases superfamily protein	AT4G10030.1
h2_chr15-_g23901	Z-SLR	Z-specific		CPSRP54	chloroplast signal recognition particle 54 kDa subunit	AT5G03940.1
h2_chr15-_g23902	Z-SLR	Z-specific		CPSRP54	chloroplast signal recognition particle 54 kDa subunit	AT5G03940.1
h2_chr15-_g23903	Z-SLR	Z-specific		CPSRP54	chloroplast signal recognition particle 54 kDa subunit	AT5G03940.1
h2_chr15+_g23904	Z-SLR	Z-specific	ancestral	None	Pentatricopeptide repeat (PPR) superfamily protein	AT1G74750.1
h2_chr15+_g23905	Z-SLR	Z-specific	ancestral	None	Ubiquitin carboxyl-terminal hydrolase-related protein	AT3G47890.1
h2_chr15+_g23906	Z-SLR	Z-sp.-P	ancestral	LBD27	LOB domain-containing protein 27	AT3G47870.1
h2_chr15-_g23907	Z-SLR	Z-specific	ancestral	BTSL1	zinc ion binding protein	AT1G74770.1
h2_chr15+_g23908	Z-SLR	Z-specific	ancestral	None	Nodulin-like / Major Facilitator Superfamily protein	AT1G74780.1

Table S9. Copy number of intact and partial duplicates of *PI* and *ARR16/17* in 12 Salicaceae species with different sex determination systems (SD). W/Y chromosomes in female heterogamety (ZW) or male heterogamety (XY) samples are enclosed in brackets if the genome assembly is not haplotype-resolved. Assemblies without brackets indicate haplotype-resolved genomes or ZZ male samples.

Species	SD system	PI		ARR17		ARR16
		complete	partial	complete	partial	complete
S. herbacea	15ZW	2 on chr02, 3 on h1chr05 and 1 on h2chr05, 1 on chr15Z	16 on chr15W	2 on chr19	no	1 on chr19, 2 on chr15W
S. purpurea	15ZW	1 on chr02, 1 on chr05	2 inverted on chr15W	2 on chr19, 4 on chr15W	9 on chr15Z	1 on chr19
S. udensis (ZZ)	15ZW	1 on chr02, 1 on chr05	not on Z	no	4 on chr15Z	1 on chr19
S. suchowensis	15ZW	1 on chr02, 1 on chr05	1 on chr15 (W)	1 on chr19	5 on chr15 (W)	1 on chr19
S. koriyanagi	15ZW	1 on chr02, 1 on chr05, 1 on chr17	1 on chr15 (W)	no	3 on chr15 (W)	1 on chr19
S. brachista	15ZW	2 on chr02, 1 on chr05	2 inverted on chr15 (W)	2 on chr19, 2 on chr15	4 on chr15 (W)	1 on chr19
S. viminalis	15ZW	1 on chr02, 1 on chr05	no	1 on chr19	1 on chr19, 3 on chr15Z	1 on chr19, 1 on chr15 (W)
S. dunnii	7XY	1 on chr02, 1 on chr05	no	2 on chr19	no	1 on chr19
S. arbutifolia	15XY	1 on chr02, 1 on chr05	no	2 on chr19	9 on chr15Y	1 on chr19
S. exigua	15XY	1 on chr02, 1 on chr05	no	3 on chr19	3 on chr15 (Y)	1 on chr19
P. trichocarpa[1]	19XY	1 on chr02, 1 on chr05	no	1 on chr19	2 on chr19 (Y)	1 on chr19
P. qiongdaoensis[1]	19ZW	3 on chr05	no	2 on chr19 (W)	2 on chr19	1 on chr19

[1] Information obtained from [51].
